# ‘Super Rehab’: can we achieve coronary artery disease regression? A feasibility study protocol

**DOI:** 10.1136/bmjopen-2023-080735

**Published:** 2023-12-12

**Authors:** John Graby, Ali Khavandi, Fiona Gillison, Theresa Smith, David Murphy, Oliver Peacock, Hugh McLeod, Amardeep Dastidar, Charalambos Antoniades, Dylan Thompson, Jonathan Carl Luis Rodrigues

**Affiliations:** 1 Cardiology, Royal United Hospitals Bath NHS Foundation Trust, Bath, UK; 2 Health, University of Bath, Bath, UK; 3 Mathematics, University of Bath, Bath, UK; 4 University of Bristol, Bristol, Bristol, UK; 5 Cardiology, North Bristol NHS Trust, Westbury on Trym, Bristol, UK; 6 Acute Multidisciplinary Imaging & Interventional Centre, University of Oxford, John Radcliffe Hospital, Oxford, UK; 7 Radiology, Royal United Hospitals Bath NHS Foundation Trust, Bath, UK

**Keywords:** Coronary heart disease, Cardiovascular imaging, Adult cardiology, Rehabilitation medicine

## Abstract

**Introduction:**

Patients diagnosed with coronary artery disease (CAD) are currently treated with medications and lifestyle advice to reduce the likelihood of disease progression and risk of future major adverse cardiovascular events (MACE). Where obstructive disease is diagnosed, revascularisation may be considered to treat refractory symptoms. However, many patients with coexistent cardiovascular risk factors, particularly those with metabolic syndrome (MetS), remain at heightened risk of future MACE despite current management.

Cardiac rehabilitation is offered to patients post-revascularisation, however, there is no definitive evidence demonstrating its benefit in a primary prevention setting. We propose that an intensive lifestyle intervention (Super Rehab, SR) incorporating high-intensity exercise, diet and behavioural change techniques may improve symptoms, outcomes, and enable CAD regression.

This study aims to examine the feasibility of delivering a multicentre randomised controlled trial (RCT) testing SR for patients with CAD, in a primary prevention setting.

**Methods and analysis:**

This is a multicentre randomised controlled feasibility study of SR versus usual care in patients with CAD. The study aims to recruit 50 participants aged 18–75 across two centres. Feasibility will be assessed against rates of recruitment, retention and, in the intervention arm, attendance and adherence to SR. Qualitative interviews will explore trial experiences of study participants and practitioners. Variance of change in CAD across both arms of the study (assessed with serial CT coronary angiography) will inform the design and power of a future, multi-centre RCT.

**Ethics and dissemination:**

Ethics approval was granted by South West—Frenchay Research Ethics Committee (reference: 21/SW/0153, 18 January 2022). Study findings will be disseminated via presentations to relevant stakeholders, national and international conferences and open-access peer-reviewed research publications.

**Trial registration number:**

ISRCTN14603929.

**Strengths and limitations of this study**

- This is a multicentre randomised controlled feasibility study.
- This study will use a mixed-methods approach, collecting quantitative and qualitative data, assessing a novel rehab programme (Super Rehab).
- This study is not designed to determine efficacy or cost-effectiveness and is limited to informing the feasibility of a larger trial of Super Rehab.
- Contemporary CT measurements of coronary artery inflammation and plaque will be acquired.
- If it is determined that a larger, multi-centre trial is feasible, this study will provide key data to improve its design.

## Introduction

Coronary artery disease (CAD) remains the most common cause of premature death in the UK and its treatment consumes substantial healthcare resources. Overall plaque burden has recently been shown to be a more sensitive marker for risk of future adverse cardiovascular event than luminal stenosis, which has historically been the focus in CAD treatment.^1^ Plaque regression, not just stabilisation, is a novel end-point in the treatment of CAD and has been used in pharmaceutical trials.^2 3^ Disease regression is the target in several other disease modalities where previously it was not thought possible, for example, type II diabetes.^4^

The role of poor diet and physical inactivity is well recognised in the pathogenesis of CAD.^5^ Lifestyle intervention studies focusing on physical activity and diet improve cardiovascular fitness (which reduces cardiovascular risk), metabolic parameters, and psychological and self-perceived health.^6–8^ Exercise training increases angina-free activity threshold, attenuates disease progression and improves event-free survival.^9^ Lifestyle intervention reduces systemic inflammation.^10^

A limited number of small studies combining a lifestyle intervention with routine care have shown the potential for CAD regression,^11–13^ indicating both a preventative and therapeutic impact.^14^ Plaque regression has been observed at 12 months and complete plaque regression has been demonstrated in case reports of patients with significant disease without coronary intervention (eg, stents).^15^ However, the current evidence-base is of insufficient quality to justify a shift to healthcare delivered preventative programmes in a chronic coronary syndrome (CCS) population (ie, established, stable CAD). Such a shift in practice requires evidence from rigorous, adequately powered, randomised controlled trials (RCT) with suitable endpoints to persuade both commissioners and healthcare professionals that this clinical pathway is cost-effective.

A healthcare delivered lifestyle intervention, cardiac rehabilitation (CR), exists for patients further down the CAD pathway who have suffered an MI or required coronary revascularisation with stents or bypass surgery. A Cochrane Review reported that while exercise-based CR appears to improve cardiovascular mortality, further research is required in higher risk CCS groups in a primary prevention setting.^9 16^ This is despite >50% of MI resulting from non-obstructive plaque, highlighting the importance of robust treatments for this patient population.^17^

Super Rehab (SR) has been designed to reflect the evolution in lifestyle intervention practices since the advent of traditional CR, incorporating the balance of recent evidence, the likelihood of patient adherence and cost benefits. Specifically SR is a cardiology-led intervention incorporating high-intensity interval training (HIIT), updated best-practice dietary advice and current behavioural change techniques.

### Exercise

HIIT, which comprises alternating periods of high intensity aerobic exercise with light intensity recovery periods, has additional benefits over conventional moderate-intensity exercise incorporated into CR.^18–20^ Aerobic capacity (VO_2_ max), is an independent predictor of all-cause mortality and cardiovascular prognosis.^6^ HIIT achieves a greater improvement in VO_2_ max, improves outcomes in CAD patients^18^ and is supported by robust safety data.^21^ SR incorporates a modified version of the Norwegian 4×4 min HIIT model, which has been received well by CAD patients.^22^

### Diet

SR integrates best-practice dietary advice for atherosclerotic cardiovascular disease and CAD patients, which has evolved to incorporate reduced refined carbohydrate and sugar content, and healthier fats and proteins. Evidence has demonstrated additional improvements in cardiovascular risk, atherosclerotic burden, systemic inflammation, glucose and blood pressure control.^23–26^

### Delivery

Traditional CR pathways have well-documented low attendance rates, consistently <50%.^27^ To improve compliance and adherence, SR adopts evidence-based strategic behavioural intervention techniques to enhance the potential for patients to adopt and sustain changes promoted during SR.^28 29^ This includes techniques that promote motivation, self-regulation and support^30^ and an autonomy supportive (patient-centred) delivery style.^31^

CT coronary angiography (CTCA) is now the mainstay investigation in new onset stable chest pain of suspected cardiac origin. CTCA provides an excellent, non-invasive tool for tracking change in coronary plaque,^32 33^ while radiation doses are low and reducing.^34^ Imaging techniques now enable the assessment of additional measures that are highly predictive of future risk. This includes: (1) *pericoronary fat attenuation index* (FAI)—a sensitive CT-derived biomarker of coronary inflammation that out-performs all other cardiovascular disease risk-stratifications^35 36^; (2) *high-risk plaque features*—morphological markers of vulnerability based on the composition and location, which predict MI risk^37^ and (3) *fractional flow reserve* (*FFR_CT_*)—a computational fluid dynamics algorithm that non-invasively estimates flow change across a plaque to determine likelihood of obstructive disease, with adverse haemodynamics denoting risk of future MI.^38^

We hypothesise that SR will improve symptoms and cause CAD regression, reducing the risk of MI and death. To change current clinical practice and integrate SR into standard care, a suitably powered RCT is required to establish efficacy using CAD-specific prognostic clinical outcomes, tracked via changes on CTCA. The present study aims to examine the feasibility of delivering a subsequent multi-centre RCT.

## Methods and analysis

### Study design

**Figure 1:**
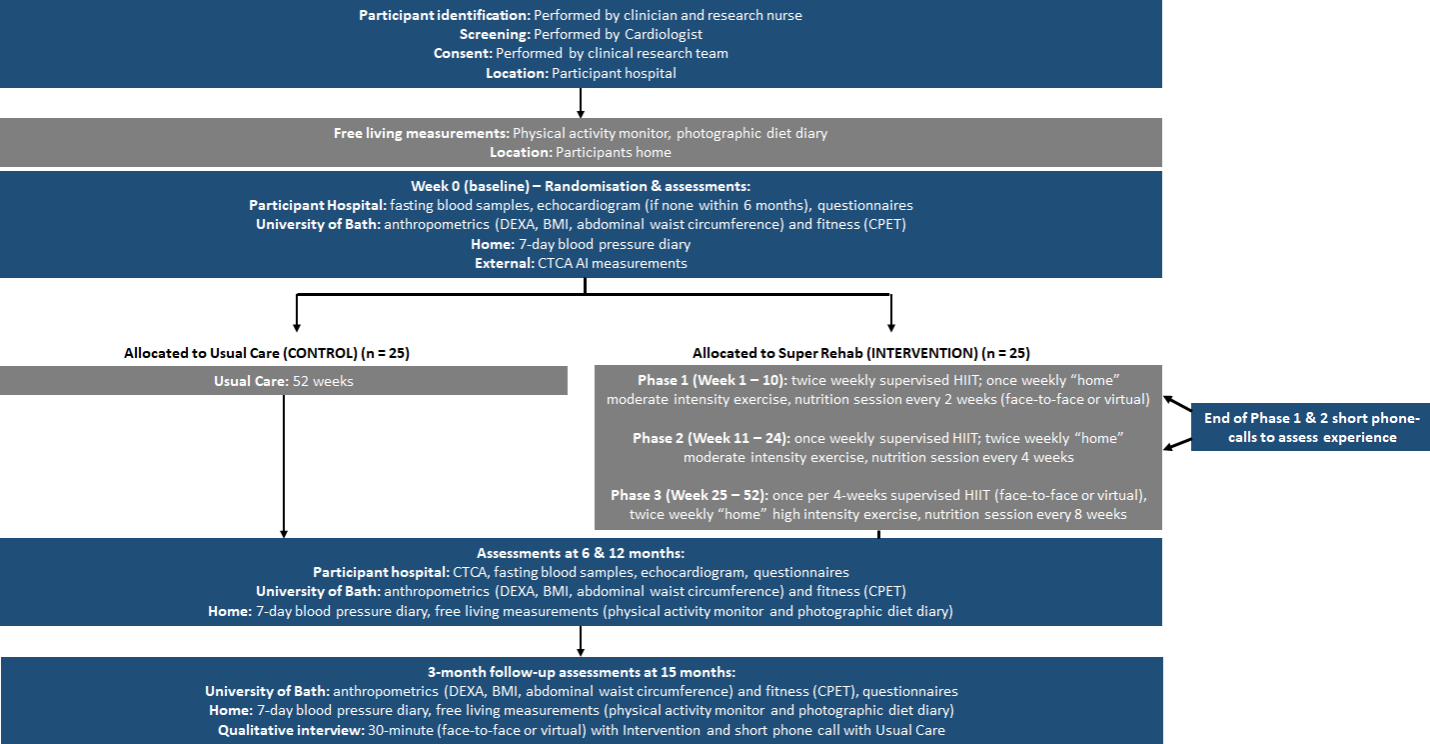
Study flow-chart.

A multicentre two parallel-arm feasibility study randomising 50 patients (1:1) to Usual Care or SR plus Usual Care to establish the feasibility of conducting a subsequent, larger and powered multicentre RCT. Figure 1 provides a study flow-chart and the protocol follows the Standard Protocol Items: Recommendations for Interventional Trials reporting guidelines.^39^

### Study aim

To establish the feasibility of delivering and evaluating a definitive RCT of SR with Usual Care vs Usual Care only in the target population.

### Study objectives

As a feasibility study, the primary objective is to assess the feasibility of delivering the proposed study design testing the efficacy of SR. Feasibility criteria assessed include:

1. Recruitment rates: the proportion of eligible patients who accept the invitation to participate.
2. Retention and adherence: the proportion of participants who complete the study and, for those in the intervention group, the proportion of offered sessions completed.
3. Acceptability of the intervention, study design and outcome measures as well as participants’ and practitioners’ experiences of SR.

Additional secondary objectives include:

1. Evaluate and refine data-collection procedures and outcomes measures.
2. Pilot the use of routine clinical data (body mass index (BMI) and HbA1c) in identifying CAD patients with MetS.
3. Obtain preliminary data for CAD regression to inform power calculations for a subsequent RCT.

### Study population and setting

Participants will be prospectively identified via routine clinically indicated CTCA lists at Royal United Hospitals Bath NHS Foundation Trust and North Bristol Trust. In line with the National Institute for Health and Care Excellence (NICE) guidelines on the assessment of patients with stable chest pain,^40^ patients with new-onset chest pain and no prior history of CAD are referred for a CTCA to assess for the presence and severity of CAD. This scan will be incorporated into screening and outcome assessments.

Patients with CAD who also have metabolic syndrome (MetS) are a particularly high-risk CAD group, representing up to half of all CAD patients^41^ and have a threefold increased risk of death.^42 43^ MetS is a cluster of conditions including abdominal obesity, raised triglycerides, high-density lipoprotein (HDL-cholesterol) or fasting glucose and hypertension.^44^ MetS not only increases the risk of CAD, but predicts more severe CAD and worse prognosis.^45 46^ Inflammation is a potent driver of atherosclerosis,^47^ risk of myocardial infarction (MI)^48 49^ as well as disease progression in MetS.^50^ Thus, CAD-MetS patients with evidence of inflammation are an important cohort with notable residual risk despite current treatments. A targeted rehabilitation programme may offer a significant benefit to this sizeable patient group.

### Inclusion criteria

1. Aged 18–75 years at consent.
2. CTCA demonstrates CAD with plaque causing a stenosis of ≥25% in ≥1 coronary artery.
3. Evidence of coronary inflammation (FAI>−70.1 HU or FAI-score (relative to age-matched and sex-matched patients) ≥75th percentile in the left anterior descending or right coronary artery or ≥90th in the circumflex^35^).
4. MetS, defined as ≥3 of the following within 6 months of CTCA^44^
  - High abdominal waist circumference (≥94 cm males, ≥80 cm females).
  - Hypertension (≥130/85 mm Hg or on treatment).
  - Diabetes mellitus or raised fasting glucose or HbA1c (glucose≥5.6 mmol/L, HbA1c≥42 mmol/L).
  - Reduced high-density lipoprotein (≤1 mmol/L males, <1.3 mmol/L females).
  - Raised triglycerides (>1.7 mmol/L).

### Exclusion criteria

Participants will not be eligible if they have conditions that preclude high-intensity exercise, including:

1. CAD requiring revascularisation.
2. Unstable angina.
3. New York Heart Association class III/IV heart failure or severe left ventricular impairment.
4. Severe valve disease.
5. Significant cardiomyopathy (as assessed by a cardiologist).
6. Severe hypertension (defined as blood pressure>180/120 mm Hg).
7. Uncontrolled cardiac arrhythmia.
8. Prior aortic dissection.
9. Recent acute pulmonary embolus, deep vein thrombosis, stroke or transient ischaemic attack.
10. Severe autonomic or peripheral neuropathy.
11. Acute systemic illness or fever.
12. Significant acute or chronic renal failure.
13. Pulmonary fibrosis or interstitial lung disease.
14. Physically unable to participate in exercise.
15. Clinically significant ECG abnormality at the screening visit, which, in the opinion of a Cardiologist, exposes the subject to risk by enrolling in the trial.

Additionally, patients with a history of prior MI or coronary revascularisation, severe coronary calcification that precludes assessment of the coronary lumen on CTCA, current participation in another intervention-based study, and pregnant or breastfeeding individuals will be excluded.

### Amendments to eligibility criteria

During the initial recruitment period, it became clear that the original eligibility criteria (high BMI (>28 kg/m^2^) and abnormal glucose control (HbA1c>42 mmol/mol or >6%)) was incompletely capturing patients with MetS. Thus, a substantial amendment was submitted to the ethics committee to allow recruitment of participants who met the international consensus criteria for MetS (outlined in the inclusion criteria above).^44^ The FAI criterion was also adjusted to ensure younger patients with high inflammation relative to age-matched and sex-matched controls (FAI-score) were captured, as well as patients recruited based on their absolute FAI value (approved 9 May 2022).

A further substantial amendment was submitted to the ethics committee to ensure participants with a raised HbA1c could participate, as fasting glucose levels were incompletely capturing patients with poor longer term glucose control (approved 12 July 2022). A non-substantial amendment clarified that participants needed to have met eligibility criteria within 6 months of their CTCA (approved 20 September 2022).

### Sample size

As a feasibility study, there is no primary outcome measure to inform a power calculation. However, a sample size of 25 in each arm is sufficient to estimate feasibility outcome proportions for acceptability rates of 64% (with 90% CI 46% to 80%) and adherence rates of 84% (90% CI 67 to 93%). The corresponding intervals for recruitment rate will be more precise as the number of potential participants will be>50.

### Screening and consent

Initial prescreening identifies potential participants who meet CAD inclusion criteria on their clinically indicated CTCA. Potential participants will be informed of the experimental protocol in writing and invited for a clinic assessment where they will complete screening with a clinician. Eligible and willing participants will be asked for written informed consent obtained according to Good Clinical Practice guidelines, and Usual Care will be instituted.

### Randomisation

Following completion of all baseline assessments, participants will be block randomised (1:1) to SR and Usual Care or Usual Care only. Randomisation will be performed using a web-based platform (Sealed Envelope: https://www.sealedenvelope.com/) in permuted blocks of four with stratification by sex using an allocation sequence generated at random.

### Assessments

Participant self-reported data will be collected at baseline, 6 months, 12 months and 15 months (post-randomisation):

1. Demographics (baseline only).
2. The EQ-5D-5L (EuroQol-5 dimensions-5 levels) questionnaire, a validated generic patient-reported outcome measure.^51^
3. Symptom assessment with the Seattle Angina Questionnaire.
4. Psychological well-being assessment with the Hospital Anxiety and Depression Scale (HADS).
5. Holistic capability assessment with the ICEpop CAPability measure for Adults (ICECAP-A).^52^
6. NHS resource use via a study specific questionnaire.

The following clinical data will be collected baseline, 6, 12 and 15 month time-points:

1. Anthropometrics, including height, weight, BMI, abdominal waist circumference.
2. Body composition, assessed with dual-energy X-ray absorptiometry (DEXA).
3. 7 day blood pressure diary.
4. Cardiopulmonary fitness assessed by cardiopulmonary exercise testing (CPET).

The following clinical data will be collected at 0, 6 and 12 months only:

1. CTCA coronary angiography, enabling analysis of luminal stenosis, plaque volume (subdivided by composition), FAI and coronary flow change (assessed by CT-derived fractional flow reserve (FFR_CT_)).
2. Fasting blood tests measuring full lipid profile, glucose, glycated haemoglobin (HbA1c) and inflammatory markers (eg, high-sensitivity C-reactive protein).
3. Cardiac structural and functional assessment with echocardiography.

The following free-living data will be collected at baseline, 6, 12 and 15 month time points:

1. 7 day photographic and/or written diet diary, to track change in dietary patterns over time.
2. 7 day physical activity monitor (GENEActiv) to characterise free-living physical activity.

**Figure 2:**
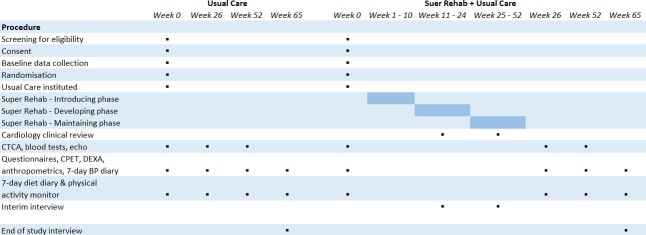
Study assessment and intervention timeline. BP, blood pressure; CPET, cardiopulmonary exercise test; CTCA, CT coronary angiohgraphy; DEXA, Dual-energy X-ray absorptiometry.

In line with the objectives of a feasibility study, we will also gather information on:

1. Recruitment and dropout rates.
2. Adherence to SR, assessed via attendance, maximum rating of perceived exertion (RPE) during HIIT, and proportion of participants opting for virtual dietary sessions.
3. A qualitative assessment of trial procedures and SR acceptability via interim and end-of study interviews with participants and practitioners. (figure 2)

### Cardiopulmonary exercise testing (CPET)

Participants in both groups will undergo a symptom-limited CPET to determine their VO_2_peak and maximum heart-rate, which will guide exercise intensity parameters for SR HIIT sessions. The CPET will be conducted on a cycle ergometer, beginning with a 5 min warm-up against zero resistance. A subsequent ramp progression is used with resistance increasing by 10–20 watts-per-minute (individualised to participant) aiming for a total duration of 8–12 min.^53^ The test discontinues at volitional exhaustion targeting a Borg rating of perceived exertion (RPE) of ≥17. A subsequent recovery phase lasts≥3 min against zero resistance. Achievement of VO_2_peak will be assumed if the following criteria are met:^53^

1. Respiratory exchange ratio≥1.10.
  or
2. Increment in VO_2_≤5 mL/kg^−1^.min^−1^ in response to increasing gradient.
  and
3. RPE ≥17.

Participants unable to complete the CPET due to a health problem raised during the exercise test or concerning discomfort exercising will discontinue involvement in the study on safety grounds. Participants who achieve an RPE<19 and a maximum heart-rate<80% of their age-predicted will be invited for a repeat test to confirm the accuracy of results.

### Control arm: usual care

All patients will be offered guideline-based recommended doses of aspirin and statin, unless contraindicated.^54 55^ Participants’ general practitioners will be provided with a study information sheet and encouraged to adjust medications as needed according to national guidelines (eg, for hypertension) if conditions change while participating in the study. Participants will also be provided routine, verbal one-off lifestyle advice in line with NICE guidance, recommending 150 min of moderate intensity exercise per week, encouraging a healthy, balanced diet, weight loss, reducing alcohol consumption and smoking cessation.

### Intervention arm: Super Rehab (plus usual care)

A detailed description of the SR protocol is provided in the online supplemental material. In short, SR is designed to enable and support successful behavioural change with a predominant focus on exercise and diet, with all sessions delivered 1-to-1. The programme will be explicitly introduced by a cardiologist, ensuring the programme is presented as a meaningful intervention to improve health outcomes. The cardiologist will introduce the concepts involved in the nutritional advice, provide advice and oversight to both the dietitian and exercise trainers, and conduct a further clinical review at the end of each intervention phase.

10.1136/bmjopen-2023-080735.supp1 Supplementary data

**Figure 3:**
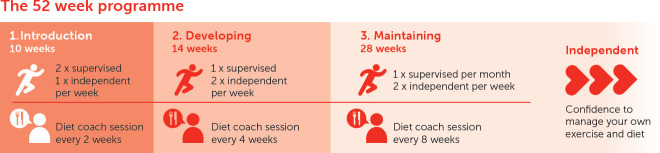
The Super Rehab programme.

SR has three phases: (1) *introduction*, (2) *developing* and (3) *maintaining* (outlined in figure 3). Practitioners will use autonomy-supportive behavioural techniques, including action planning, goal setting and self-monitoring.^28 30^ This will include frequent measurement of body metrics (blood pressure, heart-rate, BMI) to enable goal-setting and biofeedback to encourage adherence. The exercise component will be delivered in local community-based exercise facilities, with the last phase offered virtually if preferred. The dietary component will either be face-to-face or virtually according to individual participant preference.

Supervised exercise session will be split between HIIT, using the Norwegian 4×4 model for CAD patients,^22^ and resistance training. Participants will be set weekly homework exercise to do by their exercise trainer. This will comprise moderate-intensity aerobic exercise for 45 min in *introduction* and *developing* phases, and increase to higher-intensity exercise in the *maintaining* phase. A once weekly resistance exercise session using workouts learnt in the supervised session during *introduction* and *developing* will be added in the *maintaining* phase.

Dietary support will consist of 1:1 sessions with a dietitian who will work with patients using pre-session combined photographic/written diet diaries to identify incremental areas of improvement in a straightforward manner. A SR introductory educational booklet incorporating diet structure will be provided. Recognising that there is no ‘one-size-fits-all’ pattern, the dietitian will work with the patient to identify residual barriers to dietary change and potential solutions and, where indicated, continue to highlight the importance of smoking cessation. SR manuals will be provided to exercise trainers and dietitians to support the delivery of a consistent programme across centres and practitioners.

### Data analysis plan

#### Data collection

##### Qualitative

SR participants will be contacted by phone for short interim acceptability assessments at the end of ‘introduction’ and ‘developing’ phases. On completion of the study, a full interview will capture all ranges of experiences. This will seek feedback on trial participation, including barriers and facilitators to trial procedures, adherence and explore any unanticipated effects of taking part. Usual care participants will be invited for a short phone call to discuss their experience of trial participation. Encrypted interviews will be recorded, transcribed verbatim (by an external transcription company) and analysed using both narrative and thematic analysis.^56^ Anonymised patients who decline the interview will be asked a very brief set of questions on reasons for refusal.

In-depth interviews will be conducted with all practitioners involved in delivering SR to identify areas that need to be adapted or changed to implement the intervention in a future trial. Specifically, this will include their experience of delivering and supporting SR, the quality of the training resources and how participation affected their other work and relationships with patients.

##### Health economics assessment

This study will consider the feasibility of collecting the data needed for a full health economic analysis in a subsequent RCT. The primary perspective will be the NHS and personal social services,^57^ but data will also be collected on costs incurred by patients. Training-related costs for participating staff and intervention activity data will be recorded by staff delivering SR using a study-specific form. At each assessment, patients will be asked to supply data on resource use via a questionnaire based on the Client Service Receipt Inventory.^58^ Resource use data will be costed using national sources (eg, National Tariffs and Formulary). The EQ-5D-5L data provides the basis for calculating quality-adjusted life-years (QALYs) specified by NICE for use in the subsequent cost utility analysis.^51 57^ The ICECAP-A capability measure provides a complementary assessment of quality-of-life that could provide additional insight into the impact of SR on patients’ outcomes in subsequent economic evaluation.^52^

#### Data management

Once collated, all data will be stored and analysed pseudonymously using a unique identifier code. An electronic cross-referencing list will be stored on an NHS password-protected computer database at each study site. Only pseudonymised data using the unique identifier code will be used on data sets shared with external collaborators. All research records will be destroyed after 10 years.

CTCA artificial intelligence imaging techniques used in the study require image transfer off-site for analysis. For FAI analysis, images will be transferred to the Oxford Translational Cardiovascular Research Group core laboratory at the University of Oxford. We will use the existing pathway for pseudonymised image transfer via the study sponsor’s collaboration with the University of Oxford on The Oxford Risk Factors And Non Invasive Imaging Study (ORFAN), which has separately been approved by the South Central—Oxford C—Research Ethics Committee (Reference 15/SC/0545). For participants recruited at North Bristol NHS Trust, all acquired CT imaging will also be pseudononymised and transferred via PACS to the sponsor for analysis, and to Caristo (company providing FAI analysis for non-ORFAN sites). For FFR_CT_ analysis, images will be sent to HeartFlow, who’s core laboratory (California, USA) provide this analysis for NHS hospitals in standard clinical care. Existing secure information sharing protocols for standard clinically indicated scans will be used for image transfer.

#### Blinding

The nature of the intervention (SR) prevents blinding of the participants and clinicians to their trial arm. However, statistical, health economic and clinical outcome analyses (including CTCA (FAI, FFR_CT_, stenosis) and blood results) will be completed blinded to study allocation.

#### Statistical analysis

Feasibility measures will be reported with 90% CIs calculated using the Exact Binomial Method; 90% intervals are chosen (rather than 95%) because the upper limits of these measures are not of direct interest for evaluating feasibility.

For the SR group, the proportion of participants adequately completing the intervention and the average number of supervised exercise and nutritional sessions completed will be reported (by intervention phase). The number and proportion of virtual sessions opted for will also be reported. A quantitative analysis of exercise intensity and nutritional change achieved with SR will be undertaken.

The distributional properties of the continuous variables will be examined by plots. Participant characteristics and outcomes will be summarised using descriptive measures: mean (SD) or median (IQR) for symmetric or skewed continuous variables, respectively; number (percent) for categorical variables; and mean (SD) absolute and percent change for longitudinal data will be reported separately by treatment allocation. Linear regression analysis of the preliminary data for CAD regression will support power calculations for subsequent RCT. Summary statistics, including missing data, for baseline and follow-up cost data will be reported.

#### Success criteria

Feasibility will be assessed against the following success criteria:

1. Recruitment rate: ≥40% of invited eligible participants consent to participate.
2. Retention: ≥70% of participants complete the study.
3. Intervention adherence: ≥70% of SR participants complete≥75% of sessions.
4. Intervention acceptability: ≥70% of participants consider SR acceptable.
5. Streamlined criteria correctly captures >90% of patients with MetS. If this is <90%, we will examine whether any other combination of measures would be more predictive.
6. Quantitative data collection methods are feasible and acceptable to participants and no substantive issues identified within patient-reported/qualitative data that contraindicate progression to RCT.
7. Successful testing of the proposed cost-effectiveness framework and data collection facilities.
8. Preliminary data for CAD regression to guide power calculations for subsequent RCT.

Criteria 1–5 will be based on the lower 90% confidence limit (CL). For example, success criteria 1 will be met if the lower limit of the 90% exact binomial CI for the recruitment rate is ≥ 40%. Progression to a full trial will be considered if these criteria are met. If any of the progression targets are not met but fall between 60% and 100% of the target (eg, having a lower CL for recruitment rate between 24% and 40%) then reasons for this would be considered by the trial management and advisory groups, which may result in changes being made to the trial design for the full trial. If any of the criteria fell below 60% of the target (eg, a lower CL for recruitment rate of less than 24%), then progression would not occur without significant and evidence-based changes to design.

### Trial management

#### Study governance

The trial management group (TMG) will be led by the cochief investigators and include the full research team. The TMG will be responsible for overseeing the day-to-day running and management of the trial.

One independent trial advisory group (TAG) will fulfil the roles traditionally undertaken by the trial steering committee and data monitoring committee. It will comprise of an independent cardiologist, health economist, CR nurse and a patient representative. TAG will be consulted for advice (eg, regarding decisions for specific study procedures and processes, including adverse events), and will meet at the beginning, mid-point and end of the study, helping to prepare for a subsequent multicentre RCT.

Adverse events (AEs) and serious AEs (SAEs) will be recorded throughout the study, up to and including final follow-up. Review of AEs will take place during TMG meetings, discussed with the TAG and reported to the sponsor and research ethics committee in line with their guidelines.

### Patient and public involvement

The trial will incorporate a Patient Advisory Group (PAG), who aided in development of and adaptations to SR in advance of study initiation. They will hold meetings with the research team during the study to advise on study processes, and will support activities to encourage participant engagement and connectedness to the study. PAG will contribute to study dissemination events, including the lay summary of study findings, and contribute to the development of the grant application twice per day for the definitive trial.

### Ethics and dissemination

Ethics approval was granted by South West—Frenchay Research Ethics Committee on 18 January 2022 (reference: 21/SW/0153) and participants will provide written informed consent prior to any study-related activities.

Study findings will be discussed with participants and other relevant stakeholders (commissioners, charities, clinicians and members of the public), including at a planned dissemination event that will be supported by our PAG. Results will be presented at national and international conferences as well as submitted for publication in peer-reviewed journals.

## Supplementary Material

Reviewer comments

Author's manuscript
