# Supplementary material for: ‘Super Rehab’: can we achieve coronary artery disease regression? A feasibility study protocol
Source: BMJ Open. 2023 Dec 12;13(12):e080735. doi: 10.1136/bmjopen-2023-080735 (PMC10729239; doi:10.1136/bmjopen-2023-080735)
Supplement: Supplementary data [file bmjopen-2023-080735supp001.pdf]

"Super Rehab": can we achieve coronary artery disease regression? (a feasibility study) – a study protocol

Supplementary material

Super Rehab – Intervention Protocol:

Contents

1. Overview 1
2. Exercise Component - supervised Sessions: 2
3. Exercise component – homework Sessions: 3
4. Dietary component: 3
5. Behavioural Support: 4
6. References: 6

1. Overview

Super Rehab has been designed to enable and support successful behavioural change in patients with cardiovascular disease. The predominant focus is on exercise and diet, and all sessions are delivered 1-to-1. The programme will be explicitly introduced by a Cardiologist, ensuring the programme is presented as a meaningful intervention to improve health outcomes as clinician-led services have been shown to enhance participant engagement[1]. The Cardiologist will introduce the concepts involved in the nutritional advice along with the Super Rehab Booklet, provide advice and oversight to both the dietitian and exercise trainers, and conduct further patient clinical reviews at the end of each intervention phase to review cardiovascular risk factors and medications.

Super Rehab has three phases: (1) *introduction*, (2) *developing* and (3) *maintaining* (see Figure 1). *Introduction* lasts 10-weeks, targeting rapid physiological and behavioural change as a key predictor of long-term success[2]. *Developing* and *maintaining* phases constitute a tapering of direct support to engender longer-term behavioural adherence alongside residual physiological gains. This draws on the principle of enabling participant learning of lifestyle modifications in differentiated blocks[3], and studies demonstrate a 'dose-response' with frequent and sustained interventions achieving more clinically meaningful results[4,5]. Practitioners will use autonomy-supportive behavioural techniques, including action planning, goal setting and self-monitoring[6,7].

The exercise component will be delivered in local community-based exercise facilities, with the last phase offered virtually. The dietary component will either be face-to-face or virtually, depending on individual participant preference.

Phase 1 – Introduction (10 weeks); Exercise: Twice-weekly 1-hour supervised exercise sessions (described below), with once-weekly sessions of prescribed moderate-intensity 'homework'. Weekly body metrics (blood pressure, heart-rate, body mass index [BMI]) will enable goal-setting and biofeedback to encourage adherence. Diet: 30-minute educational sessions every 2-weeks. These will be delivered virtually or built into exercise sessions, minimising patient travel.

Phase 2 – Developing (14 weeks); Exercise: Reduced supervised session frequency to once-weekly, with two sessions of prescribed moderate-intensity homework per week. Body metrics measured

Graby J, *et al. BMJ Open* 2023; 13:e080735. doi: 10.1136/bmjopen-2023-080735

and fed back 2-weekly. Diet: A 30-minute "touch-base" session once/month, providing ongoing behavioural support of self-monitoring and goals.

Phase 3 - Maintaining (28 Weeks); Exercise: Supervised sessions reduced to one 4-weekly, with twice weekly prescribed sessions of homework (now higher-intensity aerobic exercise) and once weekly resistance exercise session. Body metrics assessed monthly. Participants will be offered the option of having the supervised sessions in this phase delivered virtually or continuing face-to-face. Diet: A 30-minute session 2-monthly, responding to individual barriers and changes in behaviour, where necessary, to support ongoing engagement with nutritional strategy.

Figure 1. The Super Rehab programme.

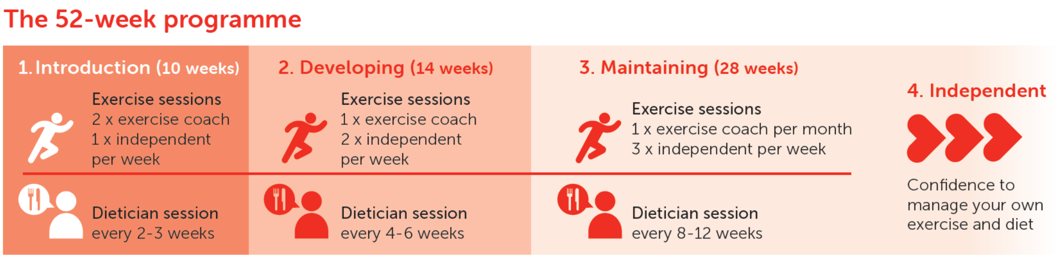

2. Exercise Component - supervised Sessions:

HIIT: Experienced trainers will lead supervised 1:1 exercise sessions incorporating high-intensity interval training (HIIT) based on the Norwegian 4x4 model[8]. This has been repeatedly used in patients with both coronary artery disease (CAD) and metabolic syndrome (MetS), and the evidence-base supports that HIIT is safe in appropriately screened patients[8–10]. At the start of each session, participants will be asked about any change in symptoms or medications and have blood pressure and heart rate measured.

The initial session will consist of a graduated, lead-in of moderate-intensity exercise prior to commencement of HIIT in subsequent sessions. Participants will be taught the Borg Rating of Perceived Exertion[11] (RPE; 6–20) to guide exercise intensity. HIIT will be performed on a static bike (or alternative cardiovascular equipment if unable to cycle) and will comprise a 3-minute warm-up; four 4-minute high-intensity intervals at RPE 15 (hard), finishing at RPE 17-18 (very hard); 3 minutes of active recovery at RPE 11-13 (somewhat hard) between each interval; and conclude with 3–5 minute recovery. Heart rate targets for HIIT (85 – 95% of heart-rate max) will be estimated with a baseline cardiopulmonary exercise test (CPET) to help gauge exercise intensity alongside RPE.

Trainers will help maintain target intensity and monitor participants throughout for concerning symptoms. Heart rate data, final RPE and duration for each high-intensity interval will be recorded for subsequent analysis. The exercise trainer will feedback data (e.g. total virtual distance cycled or power used) to participants as they work through the programme to highlight visible progress being achieved.

The goal as participants progress through the programme will be to gradually increase their overall workload (via watts or speed) on a weekly basis as they become fitter and stronger. To achieve this,

Graby J, *et al. BMJ Open* 2023; 13:e080735. doi: 10.1136/bmjopen-2023-080735

trainers will gradually increase the bike's resistance or participant's cycle speed, using heart rate response to ensure they remain in their target zone as workload increases.

Resistance Exercises: This has additional benefits, especially for blood pressure[12–14]. Sessions will therefore conclude with twenty minutes of resistance training.

This will be based on a short series of circuit training and will rotate muscle groups between sessions to allow recovery and maximise benefit. During the *introduction* phase trainers will utilise gym resources (free weights and weight machines) to build strength and confidence. In the subsequent developing phase this will switch to resistance against own body weight and bands to ensure patients learn routines that can then be undertaken in their own home in the maintaining phase and beyond.

The specific exercises are not directly prescribed to allow trainers to identify and respond to the individual needs of each participant. They will though be asked to follow this guiding framework and to record the exercises performed:

> *Introducing:* Many patients will be deconditioned, therefore initial sessions will begin with 10 – 15 repetitions at 40 – 50% of the 1 rep-max using free weights or weight machines. When ready (typically after 4 – 5 weeks), progress to 60 – 80% of the individual's 1 repetition maximum.
>
> *Developing & Maintaining:* Move to exercises against gravity / own body weight and use of exercise bands so patients should learn exercises they can start to use on their own. Circuits of 10 – 15 reps at 60 – 80% of the individual's 1 repetition maximum.

3. Exercise component – homework Sessions:

To enhance the weekly workload achieved and support long-term behaviour change, participants will be prescribed 'homework' exercise sessions. This will comprise moderate-intensity aerobic exercise (60 – 75% of heart-rate max) for 45-minutes in induction and consolidation phases (e.g., brisk walking), and increase to higher-intensity exercise (e.g., hill walking or jogging) in the maintenance phase. A once weekly resistance exercise session using workouts learnt in the supervised session during phases 1 and 2 will be added in phase 3. Participants will be provided with written guidance on effort levels, how to monitor their symptoms, and a heart-rate monitor (MyZone) to support self-directed exercise intensity at the appropriate level. These data will be reviewed at supervised exercise sessions to evaluate progress and provide support and kept for subsequent analysis.

4. Dietary component:

Nutrition therapy, as part of a comprehensive lifestyle intervention, improves and can reverse the components of MetS. The dietary intervention component comprises dietitian-led 1:1 consultations using evidence-based dietary advice for CAD-MetS patients[15–17]. This approach has been shown to improve blood pressure, insulin resistance and glycaemic control, lipid profile, systemic vascular inflammation and BMI[15,16,18–23].

Graby J, *et al. BMJ Open* 2023; 13:e080735. doi: 10.1136/bmjopen-2023-080735

Patients will complete pre-session combined photographic/written diet diaries and will be provided with an educational booklet incorporating the key dietary messages and structure. Recognising that there is no 'one-size-fits-all' pattern, the dietitian will work with participants to identify residual barriers to dietary change and potential solutions. The photographic diet diary will help highlight incremental areas for improvement in a straightforward manner. The dietitian will take a patient-centred approach to setting relevant short-term goals specific to the individual they are working with, which will be reviewed and built upon in each session.

The principles and guiding framework for the dietary advice will be:

- A focus on dietary patterns, including eating at regular mealtimes with portion control rather than "calorie counting", snack reduction and identifying healthy alternatives;
- Reduced refined carbohydrate intake, avoiding starch-based vegetables and refined grains;
- Reducing added sugars, making use of diet diaries to highlight 'hidden sugars' as well encouraging low glycaemic index foods in diabetic participants;
- Sensible drinking choices, recommending water over smoothies, diet drinks and juices, unsweetened tea/coffee and alcohol in moderation;
- Avoidance of ultra-processed foods, such as highly-processed meat and take-away meals;
- Choosing healthy sources of protein, encouraging plant-based foods such as pulses and nuts, regular oily fish, low-fat dairy products, eggs and lean unprocessed cuts of meat (if desired);
- Avoidance of 'low-fat' products, in favour of natural, whole foods with unsaturated and/or mono-saturated fats;
- Educate patients on the importance of swapping saturated fats for mono- and poly-unsaturated fats. Examples include identifying approaches to increasing natural, whole foods containing unsaturated fats, encouraging nuts, seeds, oily fish, avocado and extra-virgin olive oil;
- Increased dietary fibre, focusing on achieving this via diversifying vegetable intake, which encourages satiety, is good for gut and cardio-metabolic health and is good for blood pressure;
- Salt restriction.

Where indicated, dietitians will also continue to highlight the importance of smoking cessation.

5. Behavioural Support:

All practitioners will be required to use the following behavioural support tools in the delivery of Super Rehab.

➢ *Education*: Practitioners will reinforce the positive health outcomes that can be achieved with the suggested exercise and dietary changes the programme aims to deliver (i.e., helping patients to understand what to do, and why they are being asked to do it). This will build on their Super Rehab Booklet. Prescribed 'homework' exercises will be personalised to each individual participant, establishing how best they can incorporate this into their lifestyle.

➢ *Problem-solving:* Using the principles of a motivational interviewing, or person-centred approach, practitioners will support lifestyle changes by helping participants identify their own barriers or logistical issues to achieving their goals (e.g. money, family or work life, intolerances, time), and how they could overcome these. Example questions and techniques will be provided to trainers in their Super Rehab Manual.

- *Social Support*: Sustaining long term change is more likely when people have support from their own networks. Participants will be asked to identify their key social support (e.g. partner, friend, child), and how they could help as part of problem solving. Dietitians will be encouraged to recommend participants bring their key social support with them to dietary review sessions, and it will also be suggested that participants perform their homework exercise with them. This will help encourage and support participants, increasing the potential to maintain this lifestyle change in the longer term.

- *Goal-setting & Biofeedback:* All participants will also be set a series of longer-term goals for their involvement in the programme, typically:

  a. ≥10% weight loss with a target BMI of 18.5 – 24.9 kg/m^2
  b. A normal abdominal waist circumference (<80cm women / <94cm men)
  c. Improvement in cardiovascular fitness.

  However, during the in-programme review sessions practitioners will focus on the shorter-term, achievable, relevant goals related to the actions they can take and focus on between sessions that will help them achieve these longer-term goals. E.g., a specific change in food type in their diet rather than the broader outcome of weight loss. There will be follow-up on whether these goals have been met in subsequent sessions. Super Rehab practitioners will follow the *SMART* goal-setting principles:

  Specific – set a clear target/change for them to make (what, when & where)

  Measurable – ensure it can be assessed at future visits

  Achievable – ensure the goal is something you (and they) believe they can do

  Relevant – select goals that ensure a step-wise improvement

  Time-bound – set a realistic time-frame for them to achieve the goal(s) by

  Changes in body metrics such as weight and abdominal waist circumference will be used within sessions to feedback on performance – both for positive re-enforcement and to supplement goal-setting. The 1:1 nature of the sessions will be used to develop rapport, engender a working relationship and a level of accountability on the participant to deliver the changes asked of them.

6. References:

1. Chauhan BF, Jeyaraman M, Mann AS, Lys J, Skidmore B, Sibley KM, Abou-Setta A, Zarychanksi R. Behavior change interventions and policies influencing primary healthcare professionals' practice-an overview of reviews. *Implementation Science*. 2017;12(1).

2. Garber AJ, Abrahamson MJ, Barzilay JI, Blonde L, Bloomgarden ZT, Bush MA, Dagogo-Jack S, DeFronzo RA, Einhorn D, Fonseca VA, Garber JR, Garvey WT, Grunberger G, Handelsman Y, Hirsch IB, et al. Consensus statement by the American association of clinical endocrinologists and American college of endocrinology on the comprehensive type 2 diabetes management algorithm - 2018 executive summary. *Endocrine Practice*. 2018;24(1):91–120.

3. Pérez EA, González MP, Martínez-Espinosa RM, Vila MDM, García-Galbis MR. Practical guidance for interventions in adults with metabolic syndrome: Diet and exercise vs. changes in body composition. *International Journal of Environmental Research and Public Health*. 2019;16(18).

4. Singh N, Stewart RAH, Benatar JR. Intensity and duration of lifestyle interventions for long-term weight loss and association with mortality: A meta-analysis of randomised trials. *BMJ Open*. 2019;9(8):1–10.

5. Dutton, GR, Laitner, MH, Perri M. Lifestyle Interventions for Cardiovascular Disease Risk Reduction: A Systematic Review of the Effects of Diet Composition, Food Provision, and Treatment Modality on Weight Loss. *Curr Atheroscler Rep*. 2014;16(10):442.

6. Samdal GB, Eide GE, Barth T, Williams G, Meland E. Effective behaviour change techniques for physical activity and healthy eating in overweight and obese adults; systematic review and meta-regression analyses. *International Journal of Behavioral Nutrition and Physical Activity*. 2017;14(1):1–14.

7. Greaves CJ, Sheppard KE, Abraham C, Hardeman W, Roden M, Evans PH, Schwarz P. Systematic review of reviews of intervention components associated with increased effectiveness in dietary and physical activity interventions. *BMC Public Health*. 2011;11(1):119.

8. Taylor JL, Holland DJ, Spathis JG, Beetham KS, Wisløff U, Keating SE, Coombes JS. Guidelines for the delivery and monitoring of high intensity interval training in clinical populations. *Progress in Cardiovascular Diseases*. 2019;62(2):140–146.

9. Wewege MA, Ahn D, Yu J, Liou K, Keech A. High-intensity interval training for patients with cardiovascular disease-is it safe? A systematic review. *Journal of the American Heart Association*. 2018;7(21):1–19.

10. Swain D. Vigorous intensity exercise is essentially safe for coronary heart disease patients. *Evidence based medicine*. 2013;18(4):159–160.

11. Borg GA. Psychophysical bases of perceived exertion. *Medicine and science in sports and exercise*. 1982;14(5):377–81.

12. Ihalainen JK, Inglis A, Mäkinen T, Newton RU, Kainulainen H, Kyröläinen H, Walker S. Strength training improves metabolic health markers in older individual regardless of training frequency. *Frontiers in Physiology*. 2019;10(FEB):1–12.

13. Braith RW, Stewart KJ. Resistance exercise training: Its role in the prevention of cardiovascular disease. *Circulation*. 2006;113(22):2642–2650.

14. Schroeder EC, Franke WD, Sharp RL, Lee D chul. Comparative effectiveness of aerobic, resistance, and combined training on cardiovascular disease risk factors: A randomized controlled trial. *PLoS ONE*. 2019;14(1):1–14.

15. Unwin DJ, Tobin SD, Murray SW, Delon C, Brady AJ. Substantial and sustained improvements in blood pressure, weight and lipid profiles from a carbohydrate restricted diet: An observational study of insulin resistant patients in primary care. *International Journal of Environmental Research and Public Health*. 2019;16(15).

16. Hoyas I, Leon-Sanz M. Nutritional Challenges in Metabolic Syndrome. *Journal of Clinical Medicine*. 2019;8(9):1301.

17. Mach F, Baigent C, Catapano AL, Koskinas KC, Casula M, Badimon L, Chapman MJ, De Backer GG, Delgado V, Ference BA, Graham IM, Halliday A, Landmesser U, Mihaylova B, Pedersen TR, et al. 2019 ESC/EAS Guidelines for the management of dyslipidaemias: Lipid modification to reduce cardiovascular risk. *European Heart Journal*. 2020;41(1):111–188.

18. Dong T, Guo M, Zhang P, Sun G, Chen B. The effects of low-carbohydrate diets on cardiovascular risk factors: A meta-analysis. *PLoS ONE*. 2020;15(1):1–16.

19. DiNicolantonio JJ, OKeefe JH. Added sugars drive coronary heart disease via insulin resistance and hyperinsulinaemia: a new paradigm. *Open Heart*. 2017;4(2):e000729.

20. Threapleton DE, Greenwood DC, Evans CEL, Cleghorn CL, Nykjaer C, Woodhead C, Cade JE, Gale CP, Burley VJ. Dietary fibre intake and risk of cardiovascular disease: Systematic review and meta-analysis. *BMJ (Online)*. 2013;347(December):1–12.

21. Forouhi NG, Krauss RM, Taubes G, Willett W. Dietary fat and cardiometabolic health: Evidence, controversies, and consensus for guidance. *BMJ (Online)*. 2018;361(June):1–8.

22. Brandhorst S, Longo VD. Dietary Restrictions and Nutrition in the Prevention and Treatment of Cardiovascular Disease. *Circulation Research*. 2019;124(6):952–965.

23. Korakas E, Dimitriadis G, Raptis A, Lambadiari V. Dietary composition and cardiovascular risk: A mediator or a Bystander? *Nutrients*. 2018;10(12).

Graby J, *et al. BMJ Open* 2023; 13:e080735. doi: 10.1136/bmjopen-2023-080735
