# Supplementary material for: ‘Super Rehab’: can we achieve coronary artery disease regression? A feasibility study protocol
Source: BMJ Open. 2023 Dec 12;13(12):e080735. doi: 10.1136/bmjopen-2023-080735 (PMC10729239; doi:10.1136/bmjopen-2023-080735)
Supplement: Author's manuscript [file bmjopen-2023-080735.draft_revisions.pdf]

BMJ Open

BMJ Open is committed to open peer review. As part of this commitment we make the peer review history of every article we publish publicly available.

When an article is published we post the peer reviewers’ comments and the authors’ responses online. We also post the versions of the paper that were used during peer review. These are the versions that the peer review comments apply to.

The versions of the paper that follow are the versions that were submitted during the peer review process. They are not the versions of record or the final published versions. They should not be cited or distributed as the published version of this manuscript.

BMJ Open is an open access journal and the full, final, typeset and author-corrected version of record of the manuscript is available on our site with no access controls, subscription charges or pay-per-view fees (http://bmjopen.bmj.com).

If you have any questions on BMJ Open’s open peer review process please email info.bmjopen@bmj.com

BMJ Open

“Super Rehab”: can we achieve coronary artery disease regression? (a feasibility study) – a study protocol

Journal:	*BMJ Open*
Manuscript ID	bmjopen-2023-080735
Article Type:	Protocol
Date Submitted by the Author:	10-Oct-2023
Complete List of Authors:	Graby, John; Royal United Hospitals Bath NHS Foundation Trust, Cardiology; University of Bath, Health<br>Khavandi, Ali; Royal United Hospitals Bath NHS Foundation Trust, Cardiology<br>Gillison, Fiona; University of Bath, Health<br>Smith, Theresa; University of Bath, Mathematics<br>Murphy, David; Royal United Hospitals Bath NHS Foundation Trust, Cardiology; University of Bath, Health<br>Peacock, Oliver; University of Bath, Department for Health<br>McLeod, Hugh; University of Bristol<br>Dastidar, Amardeep; North Bristol NHS Trust, Cardiology<br>Antoniades, Charalambos; University of Oxford Radcliffe Department of Medicine,<br>Thompson, Dylan; University of Bath, Health<br>Rodrigues, Jonathan; Royal United Hospitals Bath NHS Foundation Trust, Radiology; University of Bath, Health
Keywords:	Coronary heart disease < CARDIOLOGY, Cardiovascular imaging < RADIOLOGY & IMAGING, Adult cardiology < CARDIOLOGY, Rehabilitation medicine < INTERNAL MEDICINE

SCHOLARONE™
Manuscripts

Title: "Super Rehab": can we achieve coronary artery disease regression? (a feasibility study) – a study protocol

Authors:

John Graby*[1,2], Ali Khavandi*[1], Fiona Gillison[3], Theresa Smith[4], David Murphy[1,2], Oliver Peacock[2], Hugh McLeod[5,6], Amardeep Dastidar[7], Charalambos Antoniades[8], Dylan Thompson[+2], Jonathan CL Rodrigues[+2,9]

*Denotes joint first authors, [+]denotes joint last authors.

1. Department of Cardiology, Royal United Hospitals Bath NHS Foundation Trust, Combe Park, Bath, Avon, BA1 3NG, UK.
2. Centre for Nutrition, Exercise and Metabolism (CNEM), Department for Health, University of Bath, Claverton Down, Bath, BA2 7AY, UK.
3. Centre for Motivation and Health Behaviour Change, Department for Health, University of Bath, Claverton Down, Bath, BA2 7AY, UK.
4. Department of Mathematical Sciences, University of Bath, Claverton Down, Bath, BA2 7AY, UK.
5. Population Health Sciences, Bristol Medical School, University of Bristol, BS8 1UD, UK.
6. The National Institute for Health Research Applied Research Collaboration West (NIHR ARC West) at University Hospitals Bristol and Weston NHS Foundation Trust, Bristol BS1 2NT, UK.
7. Department of Cardiology, North Bristol NHS Trust, Southmead Road, Bristol, BS10 5NB, UK.
8. Acute Multidisciplinary Imaging & Interventional Centre, University of Oxford, John Radcliffe Hospital, Oxford, OX3 9DU, UK.
9. Department of Radiology, Royal United Hospitals Bath NHS Foundation Trust, Combe Park, Bath, Avon, BA1 3NG, UK.

For peer review only

Corresponding authors:

Dr John Graby

Email: john.graby@nhs.net

Address: Department for Health, University of Bath, Claverton Down, Bath, BA2 7AY, UK.

ORCID iDs

John Graby: 0000-0002-8753-1343

Ali Khavandi: 0000-0001-6374-3782

Fiona Gillison: 0000-0002-6461-7638

Theresa Smith: 0000-0002-7085-3864

David Murphy: 0000-0003-0670-2567

Oliver Peacock: 0000-0002-2435-2903

Hugh McLeod: 0000-0002-2266-7303

Amardeep Dastidar: 0000-0002-8688-5326

Charalambos Antoniades: 0000-0002-6983-5423

Dylan Thompson: 0000-0002-6312-1518

Jonathan CL Rodrigues: 0000-0003-2744-3509

For peer review only

Abstract:

Introduction:

Patients diagnosed with coronary artery disease (CAD) are currently treated with medications and lifestyle advice to reduce the likelihood of disease progression and risk of future major adverse cardiovascular events (MACE). Where obstructive disease is diagnosed, revascularisation may be considered to treat refractory symptoms. However, many patients with co-existent cardiovascular risk factors, particularly those with metabolic syndrome (MetS), remain at heightened risk of future MACE despite current management.

Cardiac Rehabilitation is offered to patients post-revascularisation, however, there is no definitive evidence demonstrating its benefit in a primary prevention setting. We propose that an intensive lifestyle intervention (Super Rehab, SR) incorporating high-intensity exercise, diet and behavioural change techniques may improve symptoms, outcomes, and enable CAD regression.

This study aims to examine the feasibility of delivering a multi-centre randomised controlled trial (RCT) testing SR for patients with CAD, in a primary prevention setting.

Methods and analysis:

This is a multi-centre randomised controlled feasibility study of SR vs usual care in patients with CAD. The study aims to recruit 50 participants aged 18-75 across two centres. Feasibility will be assessed against rates of recruitment, retention and, in the intervention arm, attendance and adherence to SR. Qualitative interviews will explore trial experiences of study participants and practitioners. Variance of change in CAD across both arms of the study (assessed with serial CT coronary angiography) will inform the design and power of a future, multi-centre RCT.

Ethics and dissemination:

Ethics approval was granted by South West – Frenchay Research Ethics Committee (reference: 21/SW/0153, 18/01/2022). Study findings will be disseminated via presentations to relevant

For peer review only - http://bmjopen.bmj.com/site/about/guidelines.xhtml

stakeholders, national and international conferences and open-access peer-reviewed research publications.

Trial registration number ISRCTN14603929

Strengths and limitations of the study:

1. Super Rehab is a phased cardiology-led intensive programme delivered 1:1 by a multi-disciplinary team combining high-intensity interval training (HIIT), resistance exercises and dietary review sessions over 12-months.
2. This is the first study to examine the impact of an intensive lifestyle intervention (Super Rehab) on a contemporary CT measurement of coronary artery inflammation and plaque.
3. This is a multi-centre randomised controlled feasibility study using mixed methods approach (quantitative and qualitative) to provide a comprehensive assessment of Super Rehab.
4. This study will assess the feasibility to inform a subsequent larger trial and, as a feasibility study, it is not designed to determine efficacy or cost-effectiveness of Super Rehab.
5. If it is determined that a larger, multi-centre trial is feasible, this study will generate valuable qualitative and quantitative data to improve its design.

For peer review only

Manuscript

Introduction:

Coronary artery disease (CAD) remains the most common cause of premature death in the UK and its treatment consumes substantial health-care resources. Overall plaque burden has recently been shown to be a more sensitive marker for risk of future adverse cardiovascular event than luminal stenosis, which has historically been the focus in CAD treatment[1]. Plaque regression, not just stabilisation, is a novel end-point in the treatment of CAD and has been used in pharmaceutical trials[2,3]. Disease regression is the target in several other disease modalities where previously it was not thought possible, e.g. type II diabetes[4].

The role of poor diet and physical inactivity is well recognised in the pathogenesis of CAD[5]. Lifestyle intervention studies focusing on physical activity and diet improve cardiovascular fitness (which reduces cardiovascular risk), metabolic parameters, and psychological and self-perceived health[6–8]. Exercise training increases angina-free activity threshold, attenuates disease progression and improves event-free survival[9]. Lifestyle intervention reduces systemic inflammation[10].

A limited number of small studies combining a lifestyle intervention with routine care have shown the potential for CAD regression[11–13], indicating both a preventative and therapeutic impact[14]. Plaque regression has been observed at 12-months and complete plaque regression has been demonstrated in case reports of patients with significant disease without coronary intervention (e.g. stents)[15]. However, the current evidence-base is of insufficient quality to justify a shift to healthcare delivered preventative programmes in a chronic coronary syndrome (CCS) population (i.e. established, stable CAD). Such a shift in practice requires evidence from rigorous, adequately powered, randomised controlled trials (RCT) with suitable endpoints to persuade both commissioners and healthcare professionals that this clinical pathway is cost-effective.

For peer review only - http://bmjopen.bmj.com/site/about/guidelines.xhtml

A healthcare delivered lifestyle intervention, cardiac rehabilitation (CR), exists for patients further down the CAD pathway who have suffered a MI or required coronary revascularisation with stents or bypass surgery. A Cochrane Review reported that whilst exercise-based CR appears to improve cardiovascular mortality, further research is required in higher-risk CCS groups in a primary prevention setting[9,16]. This is despite >50% of MI resulting from non-obstructive plaque, highlighting the importance of robust treatments for this patient population[17].

Super Rehab (SR) has been designed to reflect the evolution in lifestyle intervention practices since the advent of traditional CR, incorporating the balance of recent evidence, the likelihood of patient adherence and cost benefits. Specifically SR is a cardiology-led intervention incorporating high-intensity interval training (HIIT), updated best-practice dietary advice and current behavioural change techniques.

Exercise: HIIT, which comprises alternating periods of high intensity aerobic exercise with light intensity recovery periods, has additional benefits over conventional moderate-intensity exercise incorporated into CR[18–20]. Aerobic capacity (VO_2 max), is an independent predictor of all-cause mortality and cardiovascular prognosis[6]. HIIT achieves a greater improvement in VO_2 max, improves outcomes in CAD patients[18] and is supported by robust safety data[21]. SR incorporates a modified version of the Norwegian 4x4 minute HIIT model, which has been received well by CAD patients[22].

Diet: SR integrates best-practice dietary advice for atherosclerotic cardiovascular disease and CAD patients, which has evolved to incorporate reduced refined carbohydrate and sugar content, and healthier fats and proteins. Evidence has demonstrated additional improvements in cardiovascular risk, atherosclerotic burden, systemic inflammation, glucose and blood pressure control[23–26].

For peer review only - http://bmjopen.bmj.com/site/about/guidelines.xhtml

Delivery: Traditional CR pathways have well-documented low attendance rates, consistently <50%[27]. To improve compliance and adherence, SR adopts evidence-based strategic behavioural intervention techniques to enhance the potential for patients to adopt and sustain changes promoted during SR[28,29]. This includes techniques that promote motivation, self-regulation and support[30] and an autonomy supportive (patient-centred) delivery style[31].

CT coronary angiography (CTCA) is now the mainstay investigation in new onset stable chest pain of suspected cardiac origin. CTCA provides an excellent, non-invasive tool for tracking change in coronary plaque[32,33], whilst radiation doses are low and reducing[34]. Imaging techniques now enable the assessment of additional measures that are highly predictive of future risk. This includes: (1) *Pericoronary fat attenuation index* (FAI) – a sensitive CT-derived biomarker of coronary inflammation that out-performs all other cardiovascular disease risk-stratifications[35,36]; (2) *High-risk plaque features* – morphological markers of vulnerability based on the composition and location, which predict MI risk[37]; and (3) *Fractional flow reserve* (FFR_{CT}) – a computational fluid dynamics algorithm that non-invasively estimates flow change across a plaque to determine likelihood of obstructive disease, with adverse haemodynamics denoting risk of future MI[38].

We hypothesise that SR will improve symptoms and cause CAD regression, reducing the risk of MI and death. To change current clinical practice and integrate SR into standard care, a suitably-powered RCT is required to establish efficacy using CAD-specific prognostic clinical outcomes, tracked via changes on CTCA. The present study aims to examine the feasibility of delivering a subsequent multi-centre RCT.

For peer review only

Methods and analysis:

Study design

A multi-centre two parallel-arm feasibility study randomising 50 patients (1:1) to Usual Care or SR plus Usual Care to establish the feasibility of conducting a subsequent, larger and powered multi-centre RCT. Figure 1 provides a study flow-chart and the protocol follows the Standard Protocol Items: Recommendations for Interventional Trials reporting guidelines[39].

Study aim

To establish the feasibility of delivering and evaluating a definitive RCT of SR with Usual Care vs Usual Care only in the target population.

Study objectives

As a feasibility study, the primary objective is to assess the feasibility of delivering the proposed study design testing the efficacy of SR. Feasibility criteria assessed include:

1. Recruitment rates: the proportion of eligible patients who accept the invitation to participate.
2. Retention and adherence: the proportion of participants who complete the study and, for those in the intervention group, the proportion of offered sessions completed.
3. Acceptability of the intervention, study design and outcome measures as well as participants' and practitioners' experiences of SR.

Additional secondary objectives include:

4. Evaluate and refine data-collection procedures and outcomes measures.

For peer review only

5. Pilot the use of routine clinical data (body mass index [BMI] and HbA1c) in identifying CAD patients with MetS.

6. Obtain preliminary data for CAD regression to inform power calculations for a subsequent RCT.

For peer review only

For peer review only - http://bmjopen.bmj.com/site/about/guidelines.xhtml

Figure 1: Study flow-chart.

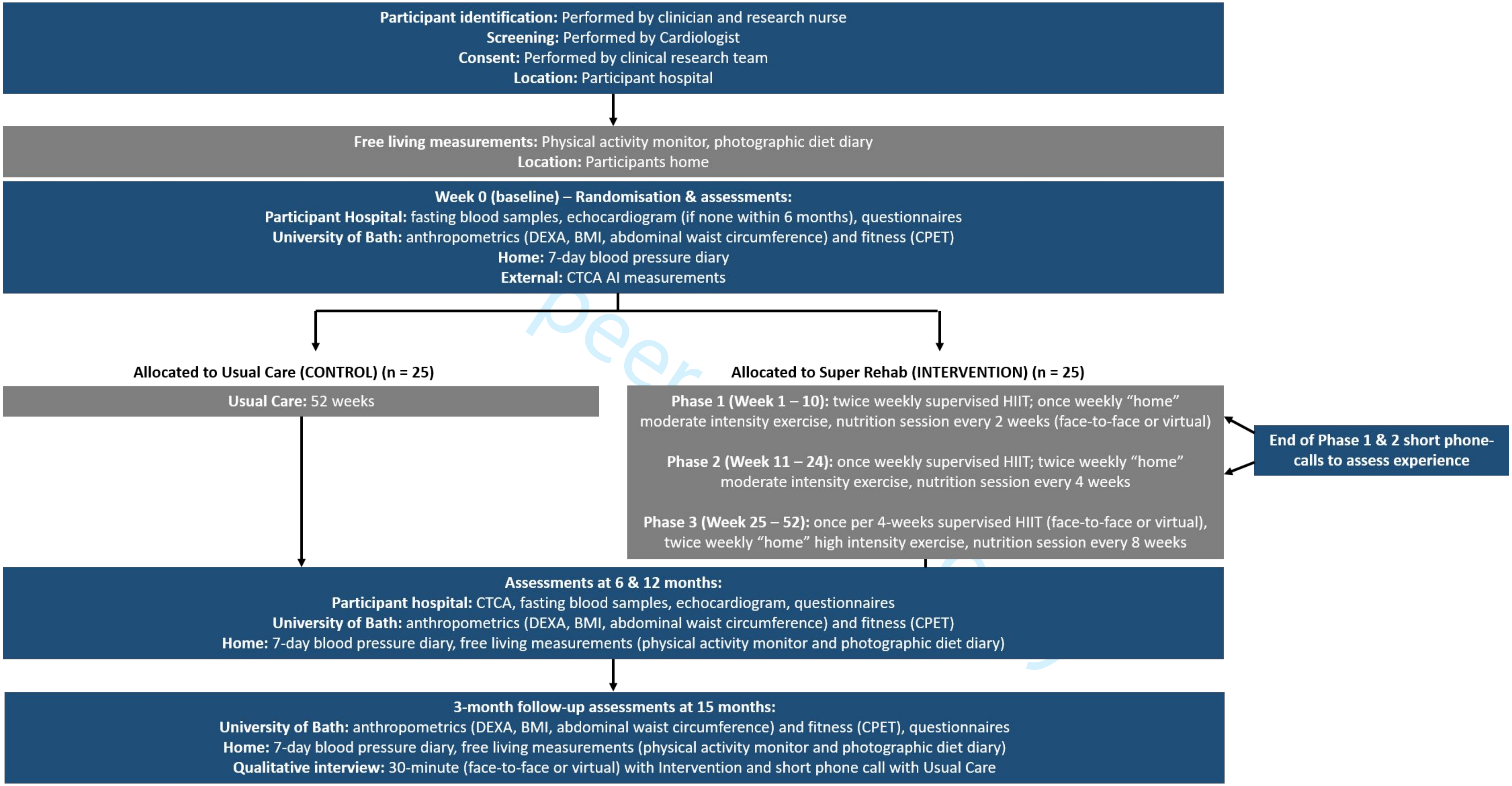

For peer review only - http://bmjopen.bmj.com/site/about/guidelines.xhtml

Study population and setting

Participants will be prospectively identified via routine clinically indicated CTCA lists at Royal United Hospitals Bath NHS Foundation Trust and North Bristol Trust. In line with the National Institute for Health and Care Excellence (NICE) guidelines on the assessment of patients with stable chest pain[40], patients with new-onset chest pain and no prior history of CAD are referred for a CTCA to assess for the presence and severity of CAD. This scan will be incorporated into screening and outcome assessments.

Patients with CAD who also have metabolic syndrome (MetS) are a particularly high-risk CAD group, representing up to half of all CAD patients[41] and have a 3-fold increased risk of death[42,43]. MetS is a cluster of conditions including abdominal obesity, raised triglycerides, high-density lipoprotein (HDL-cholesterol) or fasting glucose, and hypertension[44]. MetS not only increases the risk of CAD, but predicts more severe CAD and worse prognosis[45,46]. Inflammation is a potent driver of atherosclerosis[47], risk of myocardial infarction (MI)[48,49] as well as disease progression in MetS[50]. Thus, CAD-MetS patients with evidence of inflammation are an important cohort with notable residual risk despite current treatments. A targeted rehabilitation programme may offer a significant benefit to this sizeable patient group.

Inclusion criteria

1. Aged 18–75 years at consent;
2. CTCA demonstrates CAD with plaque causing a stenosis of ≥25% in ≥1 coronary artery;
3. Evidence of coronary inflammation (FAI > -70.1HU or FAI-score [relative to age- and sex-matched patients] ≥75th percentile in the left anterior descending or right coronary artery or ≥90th in the circumflex[35]);
4. MetS, defined as ≥3 of the following within 6 months of CTCA[44]:

For peer review only - http://bmjopen.bmj.com/site/about/guidelines.xhtml

- High abdominal waist circumference (≥94cm males, ≥80cm females);
- Hypertension (≥130/85mmHg or on treatment);
- Diabetes mellitus or raised fasting glucose or HbA1c (glucose ≥5.6mmol/L, HbA1c ≥42mmol/L);
- Reduced high-density lipoprotein (≤1mmol/L males, <1.3mmol/L females);
- Raised triglycerides (>1.7mmol/L).

Exclusion criteria

Participants will not be eligible if they have conditions that preclude high-intensity exercise, including:

1. CAD requiring revascularisation;
2. Unstable angina;
3. New York Heart Association class III/IV heart failure or severe left ventricular impairment;
4. Severe valve disease;
5. Significant cardiomyopathy (as assessed by a Cardiologist);
6. Severe hypertension (defined as blood pressure >180/120mmHg);
7. Uncontrolled cardiac arrhythmia;
8. Prior aortic dissection;
9. Recent acute pulmonary embolus, deep vein thrombosis, stroke or transient ischaemic attack;
10. Severe autonomic or peripheral neuropathy;
11. Acute systemic illness or fever;
12. Significant acute or chronic renal failure;
13. Pulmonary fibrosis or interstitial lung disease;
14. Physically unable to participate in exercise;

For peer review only - http://bmjopen.bmj.com/site/about/guidelines.xhtml

15. Clinically significant ECG abnormality at the screening visit, which, in the opinion of a Cardiologist, exposes the subject to risk by enrolling in the trial.

Additionally, patients with a history of prior MI or coronary re-vascularisation, severe coronary calcification that precludes assessment of the coronary lumen on CTCA, current participation in another intervention-based study, and pregnant or breastfeeding individuals will be excluded.

Amendments to eligibility criteria

During the initial recruitment period, it became clear that the original eligibility criteria (high BMI [>28kg/m^2] and abnormal glucose control [HbA1c >42mmol/mol or >6%]) was incompletely capturing patients with MetS. Thus, a substantial amendment was submitted to the ethics committee to allow recruitment of participants who met the international consensus criteria for MetS (outlined in the inclusion criteria above)[44]. The FAI criterion was also adjusted to ensure younger patients with high inflammation relative to age- and sex-matched controls (FAI-score) were captured, as well as patients recruited based on their absolute FAI value (approved 9/5/2022).

A further substantial amendment was submitted to the ethics committee to ensure participants with a raised HbA1c could participate, as fasting glucose levels were incompletely capturing patients with poor longer-term glucose control (approved 12/7/2022). A non-substantial amendment clarified that participants needed to have met eligibility criteria within 6-months of their CTCA (approved 20/9/2022).

Sample size

As a feasibility study there is no primary outcome measure to inform a power calculation. However, a sample size of 25 in each arm is sufficient to estimate feasibility outcome proportions for acceptability rates of 64% (with 90% confidence interval [CI] 46-80%) and adherence rates of 84%

For peer review only - http://bmjopen.bmj.com/site/about/guidelines.xhtml

(90% CI 67-93%). The corresponding intervals for recruitment rate will be more precise as the number of potential participants will be >50.

Screening and consent

Initial pre-screening identifies potential participants who meet CAD inclusion criteria on their clinically indicated CTCA. Potential participants will be informed of the experimental protocol in writing and invited for a clinic assessment where they will complete screening with a clinician. Eligible and willing participants will be asked for written informed consent obtained according to Good Clinical Practice guidelines, and Usual Care will be instituted.

Randomisation

Following completion of all baseline assessments, participants will be block randomised (1:1) to SR and Usual Care or Usual Care only. Randomisation will be performed using a web-based platform (Sealed Envelope: https://www.sealedenvelope.com/) in permuted blocks of four with stratification by sex using an allocation sequence generated at random.

Assessments (see Figure 2)

Participant self-reported data will be collected at baseline, 6-months, 12-months and 15-months (post-randomisation):

1. Demographics (baseline only).

2. The EQ-5D-5L (EuroQol-5 dimensions-5 levels) questionnaire, a validated generic patient-reported outcome measure[51].

For peer review only

3. Symptom assessment with the Seattle Angina Questionnaire.

4. Psychological well-being assessment with the Hospital Anxiety and Depression Scale (HADS).

5. Holistic capability assessment with the ICEpop CAPability measure for Adults (ICECAP-A)[52].

6. NHS resource use via a study specific questionnaire.

The following clinical data will be collected baseline, 6, 12 and 15-month time-points:

7. Anthropometrics, including height, weight, BMI, abdominal waist circumference.

8. Body composition, assessed with dual-energy X-ray absorptiometry (DEXA).

9. 7-day blood pressure diary.

10. Cardiopulmonary fitness assessed by cardiopulmonary exercise testing (CPET).

The following clinical data will be collected at 0, 6 and 12-months only:

11. CT coronary angiography, enabling analysis of luminal stenosis, plaque volume (sub-divided by composition), FAI and coronary flow change (assessed by CT-derived fractional flow reserve [FFR_{CT}]).

12. Fasting blood tests measuring full lipid profile, glucose, glycated hemoglobin (HbA1c) and inflammatory markers (e.g. high-sensitivity C-reactive protein).

13. Cardiac structural and functional assessment with echocardiography.

The following free-living data will be collected at baseline, 6, 12 and 15-month time points:

14. 7-day photographic and/or written diet diary, to track change in dietary patterns over time.

15. 7-day physical activity monitor (GENEActiv) to characterise free-living physical activity.

In line with the objectives of a feasibility study, we will also gather information on:

16. Recruitment and dropout rates.

For peer review only - http://bmjopen.bmj.com/site/about/guidelines.xhtml

17. Adherence to SR, assessed via attendance, maximum rating of perceived exertion (RPE) during HIIT, and proportion of participants opting for virtual dietary sessions.

18. A qualitative assessment of trial procedures and SR acceptability via interim and end-of study interviews with participants and practitioners.

For peer review only

For peer review only - http://bmjopen.bmj.com/site/about/guidelines.xhtml

Figure 2. Study assessment and intervention timeline. (CTCA = computed tomography coronary angiography, CPET = cardiopulmonary exercise test, DEXA = Dual-energy X-ray absorptiometry, BP = blood pressure)

	**Usual Care**				**Suer Rehab + Usual Care**						
	Week 0	*Week 26*	*Week 52*	*Week 65*	*Week 0*	*Week 1 - 10*	*Week 11 - 24*	*Week 25 - 52*	*Week 26*	*Week 52*	*Week 65*
**Procedure**											
Screening for eligibility	▪				▪						
Consent	▪				▪						
Baseline data collection	▪				▪						
Randomisation	▪				▪						
Usual Care instituted	▪				▪						
Super Rehab - Introducing phase											
Super Rehab - Developing phase											
Super Rehab - Maintaining phase											
Cardiology clinical review							▪	▪			
CTCA, blood tests, echo	▪	▪	▪		▪				▪	▪	
Questionnaires, CPET, DEXA, anthropometrics, 7-day BP diary	▪	▪	▪	▪	▪				▪	▪	▪
7-day diet diary & physical activity monitor	▪	▪	▪	▪	▪				▪	▪	▪
Interim interview							▪	▪			
End of study interview				▪							▪

Cardiopulmonary exercise testing (CPET)

Participants in both groups will undergo a symptom-limited CPET to determine their VO_2peak and maximum heart-rate, which will guide exercise intensity parameters for SR HIIT sessions. The CPET will be conducted on a cycle ergometer, beginning with a 5-minute warm-up against zero resistance. A subsequent ramp progression is used with resistance increasing by 10-20 watts-per-minute (individualised to participant) aiming for a total duration of 8-12 minutes[53]. The test discontinues at volitional exhaustion targeting a Borg rating of perceived exertion (RPE) of ≥17. A subsequent recovery phase lasts ≥3 minutes against zero resistance. Achievement of VO_2peak will be assumed if the following criteria are met[53]:

1. Respiratory exchange ratio ≥1.10;

   or

2. Increment in $VO_2 \leq 5$ mL/kg^{-1}.min^{-1} in response to increasing gradient.

   and

3. RPE ≥17;

Participants unable to complete the CPET due to a health problem raised during the exercise test or concerning discomfort exercising will discontinue involvement in the study on safety grounds. Participants who achieve an RPE <19 and a maximum heart-rate <80% of their age-predicted will be invited for a repeat test to confirm the accuracy of results.

Control Arm: Usual Care

All patients will be offered guideline-based recommended doses of aspirin and statin, unless contra-indicated[54,55]. Participants' General Practitioners will be provided with a study information sheet and encouraged to adjust medications as needed according to national guidelines (e.g. for hypertension) if conditions change whilst participating in the study. Participants will also be provided

For peer review only - http://bmjopen.bmj.com/site/about/guidelines.xhtml

routine, verbal one-off lifestyle advice in line with NICE guidance, recommending 150 minutes of moderate intensity exercise per week, encouraging a healthy, balanced diet, weight loss, reducing alcohol consumption and smoking cessation.

Intervention Arm: Super Rehab (plus Usual Care)

A detailed description of the SR protocol is provided in the supplementary material. In short, SR is designed to enable and support successful behavioural change with a predominant focus on exercise and diet, with all sessions delivered 1-to-1. The programme will be explicitly introduced by a Cardiologist, ensuring the programme is presented as a meaningful intervention to improve health outcomes. The Cardiologist will introduce the concepts involved in the nutritional advice, provide advice and oversight to both the dietitian and exercise trainers, and conduct a further clinical review at the end of each intervention phase.

SR has three phases: (1) *introduction*, (2) *developing* and (3) *maintaining* (outlined in Figure 3). Practitioners will use autonomy-supportive behavioural techniques, including action planning, goal setting and self-monitoring[28,30]. This will include frequent measurement of body metrics (blood pressure, heart-rate, BMI) to enable goal-setting and bio-feedback to encourage adherence. The exercise component will be delivered in local community-based exercise facilities, with the last phase offered virtually if preferred. The dietary component will either be face-to-face or virtually according to individual participant preference.

Supervised exercise session will be split between HIIT, using the Norwegian 4x4 model for CAD patients[22], and resistance training. Participants will be set weekly homework exercise to do by their exercise trainer. This will comprise moderate-intensity aerobic exercise for 45-minutes in *introduction* and *developing* phases, and increase to higher-intensity exercise in the *maintaining*

For peer review only - http://bmjopen.bmj.com/site/about/guidelines.xhtml

phase. A once weekly resistance exercise session using workouts learnt in the supervised session during *introduction* and *developing* will be added in the *maintaining* phase.

Dietary support will consist of 1:1 sessions with a dietitian who will work with patients using pre-session combined photographic/written diet diaries to identify incremental areas of improvement in a straightforward manner. A SR introductory educational booklet incorporating diet structure will be provided. Recognising that there is no “one-size-fits-all” pattern, the dietitian will work with the patient to identify residual barriers to dietary change and potential solutions and, where indicated, continue to highlight the importance of smoking cessation. Super Rehab manuals will be provided to exercise trainers and dietitians to support the delivery of a consistent programme across centres and practitioners.

Figure 3. The Super Rehab programme.

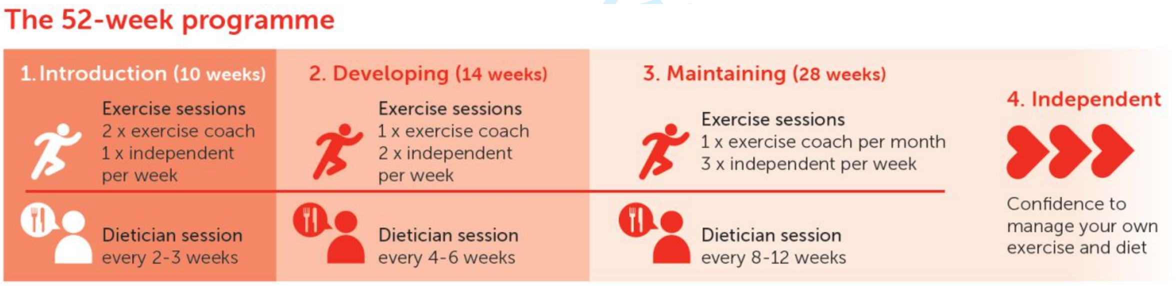

Data analysis plan

Data collection

Qualitative:

SR participants will be contacted by phone for short interim acceptability assessments at the end of “introduction” and “developing” phases. Upon completion of the study, a full interview will capture all ranges of experiences. This will seek feedback on trial participation, including barriers and

facilitators to trial procedures, adherence, and explore any unanticipated effects of taking part. Usual Care participants will be invited for a short phone call to discuss their experience of trial participation. Encrypted interviews will be recorded, transcribed verbatim (by an external transcription company) and analysed using both narrative and thematic analysis[56]. Anonymised patients who decline the interview will be asked a very brief set of questions on reasons for refusal.

In-depth interviews will be conducted with all practitioners involved in delivering SR to identify areas that need to be adapted or changed to implement the intervention in a future trial. Specifically, this will include their experience of delivering and supporting SR, the quality of the training resources, and how participation affected their other work and relationships with patients.

Health Economics Assessment:

This study will consider the feasibility of collecting the data needed for a full health economic analysis in a subsequent RCT. The primary perspective will be the NHS and personal social services[57], but data will also be collected on costs incurred by patients. Training-related costs for participating staff and intervention activity data will be recorded by staff delivering SR using a study-specific form. At each assessment, patients will be asked to supply data on resource use via a questionnaire based on the Client Service Receipt Inventory[58]. Resource use data will be costed using national sources (e.g. National Tariffs and Formulary). The EQ-5D-5L data provides the basis for calculating quality-adjusted life-years (QALYs) specified by NICE for use in the subsequent cost utility analysis[51,57]. The ICECAP-A capability measure provides a complementary assessment of quality-of-life that could provide additional insight into the impact of SR on patients' outcomes in subsequent economic evaluation[52].

For peer review only - http://bmjopen.bmj.com/site/about/guidelines.xhtml

Data Management

Once collated, all data will be stored and analysed pseudonymously using a unique identifier code. An electronic cross-referencing list will be stored on an NHS password-protected computer database at each study site. Only pseudonymised data using the unique identifier code will be used on datasets shared with external collaborators. All research records will be destroyed after 10 years.

CTCA artificial intelligence imaging techniques used in the study require image transfer off-site for analysis. For FAI analysis, images will be transferred to the Oxford Translational Cardiovascular Research Group core lab at the University of Oxford. We will use the existing pathway for pseudonymised image transfer via the study sponsor's collaboration with the University of Oxford on The Oxford Risk Factors And Non Invasive Imaging Study (ORFAN), which has separately been approved by the South Central - Oxford C - Research Ethics Committee (Reference 15/SC/0545). For participants recruited at North Bristol NHS Trust, all acquired CT imaging will also be pseudononymised and transferred via PACS to the sponsor for analysis, and to Caristo (company providing FAI analysis for non-ORFAN sites). For FFR_{CT} analysis, images will be sent to HeartFlow, who's core lab (California, US) provide this analysis for NHS hospitals in standard clinical care. Existing secure information sharing protocols for standard clinically indicated scans will be used for image transfer.

Blinding

The nature of the intervention (SR) prevents blinding of the participants and clinicians to their trial arm. However, statistical, health economic and clinical outcome analyses (including CTCA [FAI, FFR_{CT}, stenosis] and blood results) will be completed blinded to study allocation.

For peer review only - http://bmjopen.bmj.com/site/about/guidelines.xhtml

Statistical Analysis

Feasibility measures will be reported with 90% confidence intervals calculated using the Exact Binomial Method. 90% intervals are chosen (rather than 95%) because the upper limits of these measures are not of direct interest for evaluating feasibility.

For the SR group, the proportion of participants adequately completing the intervention and the average number of supervised exercise and nutritional sessions completed will be reported (by intervention phase). The number and proportion of virtual sessions opted for will also be reported. A quantitative analysis of exercise intensity and nutritional change achieved with SR will be undertaken.

The distributional properties of the continuous variables will be examined by plots. Participant characteristics and outcomes will be summarised using descriptive measures: mean (standard deviation) or median (interquartile range) for symmetric or skewed continuous variables, respectively; number (percent) for categorical variables; and mean (standard deviation) absolute and percent change for longitudinal data will be reported separately by treatment allocation. Linear regression analysis of the preliminary data for CAD regression will support power calculations for subsequent RCT. Summary statistics, including missing data, for baseline and follow-up cost data will be reported.

For peer review only

Success criteria

Feasibility will be assessed against the following success criteria:

1. Recruitment rate: ≥40% of invited eligible participants consent to participate;

2. Retention: ≥70% of participants complete the study;

3. Intervention adherence: ≥70% of SR participants complete ≥75% of sessions;

For peer review only - http://bmjopen.bmj.com/site/about/guidelines.xhtml

4. Intervention acceptability: ≥70% of participants consider SR acceptable;

5. Streamlined criteria correctly captures >90% of patients with MetS. If this is <90%, we will examine whether any other combination of measures would be more predictive;

6. Quantitative data collection methods are feasible and acceptable to participants and no substantive issues identified within patient-reported/qualitative data that contraindicate progression to RCT;

7. Successful testing of the proposed cost-effectiveness framework and data collection facilities;

8. Preliminary data for CAD regression to guide power calculations for subsequent RCT.

Criteria 1-5 will be based on the lower 90% confidence limit (CL). For example, success criteria 1 will be met if the lower limit of the 90% exact binomial confidence interval for the recruitment rate is ≥40%. Progression to a full trial will be considered if these criteria are met. If any of the progression targets are not met but fall between 60% and 100% of the target (e.g., having a lower CL for recruitment rate between 24% and 40%) then reasons for this would be considered by the trial management and advisory groups, which may result in changes being made to the trial design for the full trial. If any of the criteria fell below 60% of the target (for example a lower CL for recruitment rate of less than 24%), then progression would not occur without significant and evidence-based changes to design.

Trial management

Study Governance

The trial management group (TMG) will be led by the co-chief investigators and include the full research team. The TMG will be responsible for overseeing the day-to-day running and management of the trial.

For peer review only - http://bmjopen.bmj.com/site/about/guidelines.xhtml

One independent trial advisory group (TAG) will fulfil the roles traditionally undertaken by the trial steering committee and data monitoring committee. It will comprise of an independent cardiologist, health economist, cardiac rehabilitation nurse and a patient representative. TAG will be consulted for advice (e.g., regarding decisions for specific study procedures and processes, including adverse events), and will meet at the beginning, mid-point and end of the study, helping prepare for a subsequent multi-centre RCT.

Adverse events (AEs) and serious AEs (SAEs) will be recorded throughout the study, up to and including final follow-up. Review of AEs will take place during TMG meetings, discussed with the TAG and reported to the sponsor and research ethics committee in line with their guidelines.

Patient and public involvement

The trial will incorporate a Patient Advisory Group (PAG), who aided in development of and adaptations to SR in advance of study initiation. They will hold meetings with the research team during the study to advise on study processes, and will support activities to encourage participant engagement and connectedness to the study. PAG will contribute to study dissemination events, including the lay summary of study findings, and contribute to the development of the grant application bid for the definitive trial.

Ethics and dissemination:

Ethics approval was granted by South West – Frenchay Research Ethics Committee on 18th January 2022 (reference: 21/SW/0153) and participants will provide written informed consent prior to any study-related activities.

Study findings will be discussed with participants and other relevant stakeholders (commissioners, charities, clinicians and members of the public), including at a planned

For peer review only - http://bmjopen.bmj.com/site/about/guidelines.xhtml

dissemination event that will be supported by our PAG. Results will be presented at national and international conferences as well as submitted for publication in peer-reviewed journals.

For peer review only

For peer review only - http://bmjopen.bmj.com/site/about/guidelines.xhtml

Acknowledgements:

The authors would like to thank Jane Carter, Sarah Stratton and Jamie Ford, key members of the sponsor's Research & Development department who were instrumental in study set-up; all the personal trainers and dietitians at the participating sites; all members of the cardiac imaging and physiology teams at the participating sites, in particular James Willis at the Royal United Hospitals Bath; our lead PPI lead Dr Lisa Austin and Lead Representative Andy Shield, as well as all the members of the PAG and TAG.

Contributors:

JG, AK, DT, JCLR, and FG led on the conception, design and writing of the study. JCLR and DT led the grant application and, as co-chief investigators, take overall responsibility for the trial. JG led on the writing of the study protocol with substantial contributions to the design, writing, critical review of intellectual content and final manuscript approval from DT, JCLR, AK, FG, TS, HM, DM, AD, OP and CA. TS and DT provided statistical expertise in the study design and development stages of the project and the protocol. AK, JG and DT led on Super Rehab conception and design. JG, DT, AK, FG, CA and JCLR made substantial contributions to the trial design and management. FG led on the qualitative study design. HM was responsible for the health economic aspects of the study design and AK, AD, JG and DM were responsible for providing expert clinical support. All authors agree to be accountable for their work.

Funding:

Royal United Hospitals Bath NHS Foundation Trust are the sponsors on behalf of the funder, who is the NIHR as stated below. This paper presents independent research funded by the National Institute for Health Research (NIHR) under its Research for Patient Benefit (RfPB) Programme (Grant

For peer review only - http://bmjopen.bmj.com/site/about/guidelines.xhtml

Reference Number NIHR202811). Further funding was provided by the sponsor's hospital charity, RUHX.

Disclaimer:

The views expressed are those of the author(s) and not necessarily those of the NHS, the NIHR, or the Department of Health and Social Care.

Competing interests:

JCLR reports consulting fees from NHSX, speakers fees from Sanofi, physician services from HeartFlow and is co-founder and share-holder of Heart & Lung Imaging LTD, all outside the submitted work. JG reports speaker fees from Sanofi outside the submitted work. CA is a founder, shareholder, and director of Caristo Diagnostics, a CT image analysis company that provides the perivascular fat attenuation index (FAI) analysis used in the study. FAI is subject to patent US10,695,023B2 and patent applications PCT/GB2017/053262, GB2018/1818049.7, GR20180100490, and GR20180100510, licensed through exclusive license to Caristo Diagnostics.

Patient consent for publication:

Not required.

Ethics approval:

Ethics approval was granted by South West – Frenchay Research Ethics Committee on 18th January 2022 (reference: 21/SW/0153).

For peer review only - http://bmjopen.bmj.com/site/about/guidelines.xhtml

Provenance and peer review:

Not commissioned; peer reviewed for ethical and funding approval prior to submission.

Word Count:

4,103

References:

1. Mortensen MB, Dzaye O, Steffensen FH, Bøtker HE, Jensen JM, Rønnow Sand NP, Kragholm KH, Sørensen HT, Leipsic J, Mæng M, Blaha MJ NB. Impact of Plaque Burden Versus Stenosis on Ischemic Events in Patients With Coronary Atherosclerosis. *J Am Coll Cardiol*. 2020;76(24):2803-2813. doi:10.1016/j.jacc.2020.10.021

2. Bhindi R, Guan M, Zhao Y, Humphries KH, Mancini GBJ. Coronary atheroma regression and adverse cardiac events: A systematic review and meta-regression analysis. *Atherosclerosis*. 2019;284(March):194-201. doi:10.1016/j.atherosclerosis.2019.03.005

3. Budoff MJ, Bhatt DL, Kinninger A, et al. Effect of icosapent ethyl on progression of coronary atherosclerosis in patients with elevated triglycerides on statin therapy: final results of the EVAPORATE trial. *Eur Heart J*. Published online 2020:1-8. doi:10.1093/eurheartj/ehaa652

4. Hallberg SJ, Gershuni VM, Athinarayanan SJ. Reversing type 2 diabetes: A narrative review of the evidence. *Nutrients*. 2019;11(4):1-16. doi:10.3390/nu11040766

5. Spring B, Moller AC, Colangelo LA, et al. Healthy Lifestyle Change and Subclinical Atherosclerosis in Young Adults. *Circulation*. 2014;130(1):10-17. doi:10.1161/circulationaha.113.005445

6. Jonathan Myers, Paul McAuley, Carl J Lavie, Jean-Pierre Despres, Ross Arena PK. Physical activity and cardiorespiratory fitness as major markers of cardiovascular risk: their independent and interwoven importance to health status. *Prog Cardiovasc Dis* . 2015;57(4):306-314.

7. Dale, H, Brassington, L and King K. The impact of healthy lifestyle interventions on mental health and wellbeing: a systematic review. *Mental Health Review Journal*. 2014;19(1):1-26.

8. Rauch B, Davos CH, Doherty P, et al. The prognostic effect of cardiac rehabilitation in the era of acute revascularisation and statin therapy: A systematic review and meta-analysis of randomized and non-randomized studies - The Cardiac Rehabilitation Outcome Study (CROS). *Eur J Prev Cardiol*. 2016;23(18):1914-1939. doi:10.1177/2047487316671181

For peer review only - http://bmjopen.bmj.com/site/about/guidelines.xhtml

9. Anderson L, Oldridge N, Thompson DR, et al. Exercise-Based Cardiac Rehabilitation for Coronary Heart Disease Cochrane Systematic Review and Meta-Analysis. *J Am Coll Cardiol*. 2016;67(1):1-12. doi:10.1016/j.jacc.2015.10.044

10. Michael G. Flynn, Brian K. McFarlin and MMM. The Anti-Inflammatory Actions of Exercise Training. *Am J Lifestyle Med*. 2007;1(3):220–235.

11. Ornish D, Scherwitz LW, Billings JH, et al. Intensive lifestyle changes for reversal of coronary heart disease. *J Am Med Assoc*. 1998;280(23):2001-2007. doi:10.1001/jama.280.23.2001

12. Tani S, Nagao K, Anazawa T, et al. Coronary plaque regression and lifestyle modification in patients treated with pravastatin - Assessment mainly by daily aerobic exercise and an increase in the serum level of high-density lipoprotein cholesterol. *Circulation Journal*. 2010;74(5):954-961. doi:10.1253/circj.CJ-09-0705

13. Henzel J, Kępka C, Kruk M, et al. High-Risk Coronary Plaque Regression After Intensive Lifestyle Intervention in Nonbstructive Coronary Disease: A Randomized Study. *JACC Cardiovasc Imaging*. Published online 2021. doi:10.1016/j.jcmg.2020.10.019

14. Winzer EB, Woitek F, Linke A. Physical activity in the prevention and treatment of coronary artery disease. *J Am Heart Assoc*. 2018;7(4):1-15. doi:10.1161/JAHA.117.007725

15. Lee HS, Chang M, Piccinelli M, Koo BK. Complete regression of coronary atherosclerosis. *Eur Heart J*. 2020;41(2):230. doi:10.1093/eurheartj/ehz792

16. Long L, Anderson L, Am D, et al. Exercise-based cardiac rehabilitation for adults with stable angina. 2018;(2). doi:10.1002/14651858.CD012786.pub2.

17. Fishbein MC, Siegel RJ. How Big Are Coronary Atherosclerotic Plaques That Rupture? *Circulation*. 1996;94(10):2662-2666. doi:10.1161/01.CIR.94.10.2662

18. Rognmo Ø, Hetland E, Helgerud J, Hoff J SSA. High intensity aerobic interval exercise is superior to moderate intensity exercise for increasing aerobic capacity in patients with coronary artery disease. *Eur J Cardiovasc Prev Rehabil*. 2004;11(3):216-222.

19. Kraal JJ, Vromen T, Spee R, Kemps HMC, Peek N. The influence of training characteristics on the effect of exercise training in patients with coronary artery disease : Systematic review and meta-regression analysis. *Int J Cardiol*. 2017;245:52-58. doi:10.1016/j.ijcard.2017.07.051

20. Tjønna AE, Lee SJ, Rognmo Ø, et al. Aerobic interval training versus continuous moderate exercise as a treatment for the metabolic syndrome: A pilot study. *Circulation*. 2008;118(4):346-354. doi:10.1161/CIRCULATIONAHA.108.772822

21. Wewege MA, Ahn D, Yu J, Liou K, Keech A. High-intensity interval training for patients with cardiovascular disease-is it safe? A systematic review. *J Am Heart Assoc*. 2018;7(21):1-19. doi:10.1161/JAHA.118.009305

22. Taylor JL, Holland DJ, Spathis JG, et al. Guidelines for the delivery and monitoring of high intensity interval training in clinical populations. *Prog Cardiovasc Dis*. 2019;62(2):140-146. doi:10.1016/j.pcad.2019.01.004

23. Brandhorst S, Longo VD. Dietary Restrictions and Nutrition in the Prevention and Treatment of Cardiovascular Disease. *Circ Res*. 2019;124(6):952-965. doi:10.1161/CIRCRESAHA.118.313352

For peer review only - http://bmjopen.bmj.com/site/about/guidelines.xhtml

24. Malinowski B, Zalewska K, Węsierska A, et al. Intermittent fasting in cardiovascular disorders—an overview. *Nutrients*. 2019;11(3):1-18. doi:10.3390/nu11030673

25. Unwin DJ, Tobin SD, Murray SW, Delon C, Brady AJ. Substantial and sustained improvements in blood pressure, weight and lipid profiles from a carbohydrate restricted diet: An observational study of insulin resistant patients in primary care. *Int J Environ Res Public Health*. 2019;16(15). doi:10.3390/ijerph16152680

26. Dong T, Guo M, Zhang P, Sun G, Chen B. The effects of low-carbohydrate diets on cardiovascular risk factors: A meta-analysis. *PLoS One*. 2020;15(1):1-16. doi:10.1371/journal.pone.0225348

27. NACR. The national audit of cardiac rehabilitation: Annual statistical report. *National Audit of Cardiac Rehabilitation*. Published online 2019. https://www.bhf.org.uk/informationsupport/publications/statistics/national-audit-of-cardiac-rehabilitation-quality-and-outcomes-report-2019 [Acessed 20 Dec 2019]

28. Samdal GB, Eide GE, Barth T, Williams G, Meland E. Effective behaviour change techniques for physical activity and healthy eating in overweight and obese adults; systematic review and meta-regression analyses. *International Journal of Behavioral Nutrition and Physical Activity*. 2017;14(1):1-14. doi:10.1186/s12966-017-0494-y

29. Jerry Suls, Jazmin N. Mogavero, Louise Falzon, Linda S. Pescatello, Emily A. Hennessy & KWD. Health behaviour change in cardiovascular disease prevention and management: meta-review of behaviour change techniques to affect self-regulation. *Health Psychol Rev*. 2020;14(1):43-65.

30. Greaves CJ, Sheppard KE, Abraham C, et al. Systematic review of reviews of intervention components associated with increased effectiveness in dietary and physical activity interventions. *BMC Public Health*. 2011;11(1):119. doi:10.1186/1471-2458-11-119

31. Michie, S, Ashford, S, Sniehotta, FF, Dombrowski, SU, Bishop, A, French D. A refined taxonomy of behaviour change techniques to help people change their physical activity and healthy eating behaviours: the CALO-RE taxonomy. *Psychol Health*. 2011;26(11):1479-1498.

32. Tarkin JM, Dweck MR, Rudd JHF. Imaging as a surrogate marker of drug efficacy in cardiovascular disease. *Heart*. 2019;105(7):567-578. doi:10.1136/heartjnl-2017-311213

33. Abdelrahman KM, Chen MY, Dey AK, et al. Coronary Computed Tomography Angiography From Clinical Uses to Emerging Technologies: JACC State-of-the-Art Review. *J Am Coll Cardiol*. 2020;76(10):1226-1243. doi:10.1016/j.jacc.2020.06.076

34. Stocker TJ, Deseive S, Leipsic J, et al. Reduction in radiation exposure in cardiovascular computed tomography imaging: Results from the PROspective multicenter registry on radiaTion dose Estimates of cardiac CT angIOgraphy in daily practice in 2017 (PROTECTION VI). *Eur Heart J*. 2018;39(41):3715-3723. doi:10.1093/eurheartj/ehy546

35. Oikonomou EK, Marwan M, Desai MY, et al. Non-invasive detection of coronary inflammation using computed tomography and prediction of residual cardiovascular risk (the CRISP CT study): a post-hoc analysis of prospective outcome data. *The Lancet*. 2018;392(10151):929-939. doi:10.1016/S0140-6736(18)31114-0

For peer review only - http://bmjopen.bmj.com/site/about/guidelines.xhtml

36. Antoniades C, Antonopoulos AS, Deanfield J. Imaging residual inflammatory cardiovascular risk. *Eur Heart J*. 2020;41(6):748-758. doi:10.1093/eurheartj/ehz474

37. Hoffmann U, Moselewski F, Nieman K, et al. Noninvasive Assessment of Plaque Morphology and Composition in Culprit and Stable Lesions in Acute Coronary Syndrome and Stable Lesions in Stable Angina by Multidetector Computed Tomography. *J Am Coll Cardiol*. 2006;47(8):1655-1662. doi:10.1016/j.jacc.2006.01.041

38. Lee JM, Choi G, Koo BK, et al. Identification of High-Risk Plaques Destined to Cause Acute Coronary Syndrome Using Coronary Computed Tomographic Angiography and Computational Fluid Dynamics. *JACC Cardiovasc Imaging*. 2019;12(6):1032-1043. doi:10.1016/j.jcmg.2018.01.023

39. Chan A wen, Tetzlaff JM, Altman DG. 2013 Statement : Defining Standard Protocol Items for Clinical Trials. *Ann Intern Med*. 2016;158(3):200-207. doi:10.7326/0003-4819-158-3-201302050-00583.Requests

40. NICE. Recent-onset chest pain of suspected cardiac origin: assessment and diagnosis. *Clinical guideline [CG95]*. 2016;(November 2016).

41. Montazerifar F, Bolouri A, Mozaffar MM, Karajibani M. The prevalence of metabolic syndrome in coronary artery disease patients. *Cardiol Res*. 2016;7(6):202-208. doi:10.14740/cr507w

42. Espinola-Klein C, Rupprecht HJ, Bickel C, et al. Impact of Metabolic Syndrome on Atherosclerotic Burden and Cardiovascular Prognosis. *American Journal of Cardiology*. 2007;99(12):1623-1628. doi:10.1016/j.amjcard.2007.01.049

43. Lakka HM, Laaksonen DE, Lakka TA, et al. The metabolic syndrome and total and cardiovascular disease mortality in middle-aged men. *J Am Med Assoc*. 2002;288(21):2709-2716. doi:10.1001/jama.288.21.2709

44. Alberti KGMM, Eckel RH, Grundy SM, et al. Harmonizing the metabolic syndrome: A joint interim statement of the international diabetes federation task force on epidemiology and prevention; National heart, lung, and blood institute; American heart association; World heart federation; International . *Circulation*. 2009;120(16):1640-1645. doi:10.1161/CIRCULATIONAHA.109.192644

45. Mahalle N, Garg MK, Naik SS, Kulkarni M V. Association of metabolic syndrome with severity of coronary artery disease. *Indian J Endocrinol Metab*. 2014;18(5):708-70814. doi:10.4103/2230-8210.139238

46. Hulten EA, Bittencourt MS, Preston R, et al. Obesity, metabolic syndrome and cardiovascular prognosis: From the Partners coronary computed tomography angiography registry. *Cardiovasc Diabetol*. 2017;16(1):1-11. doi:10.1186/s12933-017-0496-8

47. Peter Libby. Inflammation in atherosclerosis. *Nature*. 2002;420(6917):868-874.

48. Libby P. Mechanisms of Acute Coronary Syndromes and Their Implications for Therapy. *New England Journal of Medicine*. 2013;368(21):2004-2013. doi:10.1056/nejmra1216063

49. Silva D, Pais de Lacerda A. High-sensitivity C-reactive protein as a biomarker of risk in coronary artery disease. *Revista Portuguesa de Cardiologia (English Edition)*. 2012;31(11):733-745. doi:10.1016/j.repce.2012.09.006

For peer review only - http://bmjopen.bmj.com/site/about/guidelines.xhtml

50. Hotamisligil GS. Inflammation and metabolic disorders. *Nature*. 2006;14(444 (7121)):860-867.

51. Lloyd MHCGA. Development and preliminary testing of the new five-level version of EQ-5D (EQ-5D-5L). Published online 2011:1727-1736. doi:10.1007/s11136-011-9903-x

52. Flynn, T, Huynh, E, Peters, T, Al-Janabi, H, Clemens, S, Moody, A, Coast J. Scoring the ICECAP-A capability instrument: estimation of a UK general population tariff. *Health Econ*. 2015;24:258-269.

53. Thompson D, Markovitch D, Betts JA, Mazzatti D, Turner J, Tyrrell RM. Time course of changes in inflammatory markers during a 6-mo exercise intervention in sedentary middle-aged men: A randomized-controlled trial. *J Appl Physiol*. 2010;108(4):769-779. doi:10.1152/japplphysiol.00822.2009

54. Knuuti J, Wijns W, Achenbach S, et al. 2019 ESC guidelines for the diagnosis and management of chronic coronary syndromes. *Eur Heart J*. 2020;41(3):407-477. doi:10.1093/eurheartj/ehz425

55. NICE. Surveillance report 2016 – Stable angina: management (2011) NICE guideline CG126. 2016;(April).

56. Braun V, Clarke V. What can "thematic analysis" offer health and wellbeing researchers? *Int J Qual Stud Health Well-being*. 2014;9(1):26152. doi:10.3402/qhw.v9.26152

57. The National Institute for Health and Care. Guide to the methods of technology appraisal. *Nice*. 2018;(April 2013):1-93. http://doi.org/10.1183/13993003.01815-2018

58. PSSRU. Personal Social Services Research Unit Client Service Receipt Inventory (CSRI). https://www.pssru.ac.uk/csri/client-service-receipt-inventory/

Figure legends

Figure 1: Study flow-chart.

Figure 2. Study assessment and intervention timeline. (CTCA = computed tomography coronary angiohgraphy, CPET = cardiopulmonary exercise test, DEXA = Dual-energy X-ray absorptiometry, BP = blood pressure)

Figure 3. The Super Rehab programme.

For peer review only - http://bmjopen.bmj.com/site/about/guidelines.xhtml

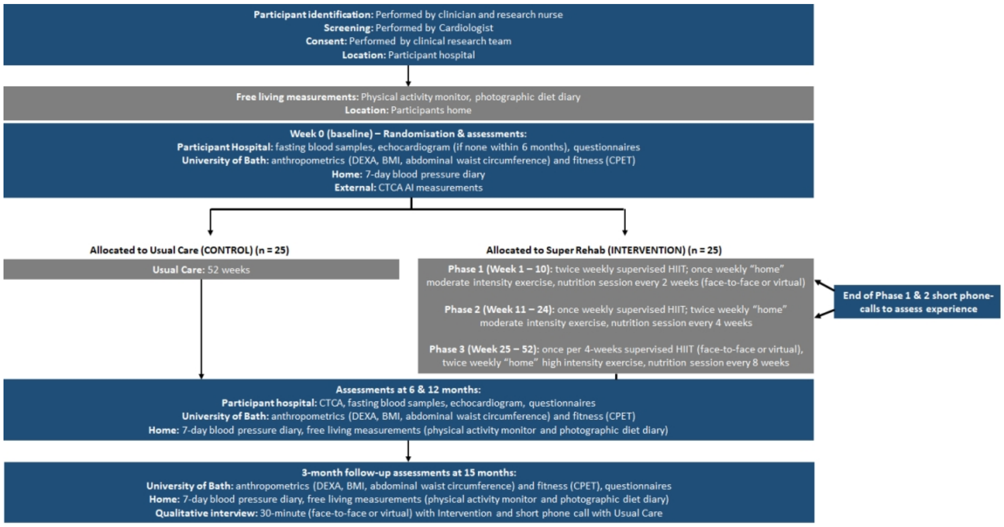

Figure 1: Study flow-chart.

303x159mm (120 x 120 DPI)

	Usual Care				Suer Rehab + Usual Care						
	Week 0	*Week 26*	*Week 52*	*Week 65*	*Week 0*	*Week 1 - 10*	*Week 11 - 24*	*Week 25 - 52*	*Week 26*	*Week 52*	*Week 65*
**Procedure**											
Screening for eligibility	▪				▪						
Consent	▪				▪						
Baseline data collection	▪				▪						
Randomisation	▪				▪						
Usual Care instituted	▪				▪						
Super Rehab - Introducing phase						▬					
Super Rehab - Developing phase							▬				
Super Rehab - Maintaining phase								▬			
Cardiology clinical review							▪	▪			
CTCA, blood tests, echo	▪	▪	▪		▪				▪	▪	
Questionnaires, CPET, DEXA, anthropometrics, 7-day BP diary	▪	▪	▪	▪	▪				▪	▪	▪
7-day diet diary & physical activity monitor	▪	▪	▪	▪	▪				▪	▪	▪
Interim interview							▪	▪			
End of study interview				▪							▪

Figure 2. Study assessment and intervention timeline. (CTCA = computed tomography coronary angiohgraphy, CPET = cardiopulmonary exercise test, DEXA = Dual-energy X-ray absorptiometry, BP = blood pressure)

273x101mm (120 x 120 DPI)

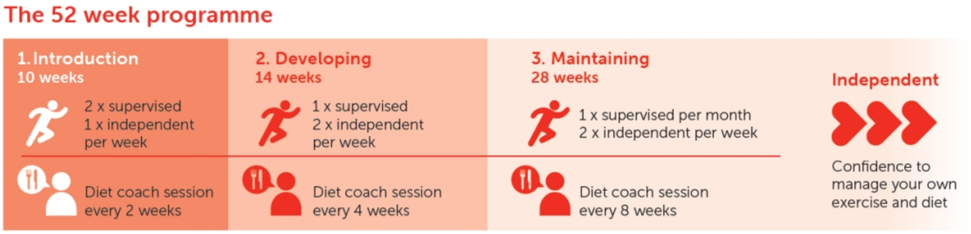

Figure 3. The Super Rehab programme.

115x28mm (300 x 300 DPI)

"Super Rehab": can we achieve coronary artery disease regression? (a feasibility study) – a study protocol

Supplementary material

Super Rehab – Intervention Protocol:

Contents

1. Overview 1
2. Exercise Component - supervised Sessions: 2
3. Exercise component – homework Sessions: 3
4. Dietary component: 3
5. Behavioural Support: 4
6. References: 7

1. Overview

Super Rehab has been designed to enable and support successful behavioural change in patients with cardiovascular disease. The predominant focus is on exercise and diet, and all sessions are delivered 1-to-1. The programme will be explicitly introduced by a Cardiologist, ensuring the programme is presented as a meaningful intervention to improve health outcomes as clinician-led services have been shown to enhance participant engagement[1]. The Cardiologist will introduce the concepts involved in the nutritional advice along with the Super Rehab Booklet, provide advice and oversight to both the dietitian and exercise trainers, and conduct further patient clinical reviews at the end of each intervention phase to review cardiovascular risk factors and medications.

Super Rehab has three phases: (1) *introduction*, (2) *developing* and (3) *maintaining* (see Figure 1). *Introduction* lasts 10-weeks, targeting rapid physiological and behavioural change as a key predictor of long-term success[2]. *Developing* and *maintaining* phases constitute a tapering of direct support to engender longer-term behavioural adherence alongside residual physiological gains. This draws on the principle of enabling participant learning of lifestyle modifications in differentiated blocks[3], and studies demonstrate a 'dose-response' with frequent and sustained interventions achieving more clinically meaningful results[4,5]. Practitioners will use autonomy-supportive behavioural techniques, including action planning, goal setting and self-monitoring[6,7].

The exercise component will be delivered in local community-based exercise facilities, with the last phase offered virtually. The dietary component will either be face-to-face or virtually, depending on individual participant preference.

Phase 1 – Introduction (10 weeks); Exercise: Twice-weekly 1-hour supervised exercise sessions (described below), with once-weekly sessions of prescribed moderate-intensity 'homework'. Weekly body metrics (blood pressure, heart-rate, body mass index [BMI]) will enable goal-setting and biofeedback to encourage adherence. Diet: 30-minute educational sessions every 2-weeks. These will be delivered virtually or built into exercise sessions, minimising patient travel.

Phase 2 – Developing (14 weeks); Exercise: Reduced supervised session frequency to once-weekly, with two sessions of prescribed moderate-intensity homework per week. Body metrics measured

For peer review only - http://bmjopen.bmj.com/site/about/guidelines.xhtml

and fed back 2-weekly. Diet: A 30-minute “touch-base” session once/month, providing ongoing behavioural support of self-monitoring and goals.

Phase 3 - Maintaining (28 Weeks); Exercise: Supervised sessions reduced to one 4-weekly, with twice weekly prescribed sessions of homework (now higher-intensity aerobic exercise) and once weekly resistance exercise session. Body metrics assessed monthly. Participants will be offered the option of having the supervised sessions in this phase delivered virtually or continuing face-to-face. Diet: A 30-minute session 2-monthly, responding to individual barriers and changes in behaviour, where necessary, to support ongoing engagement with nutritional strategy.

Figure 1. The Super Rehab programme.

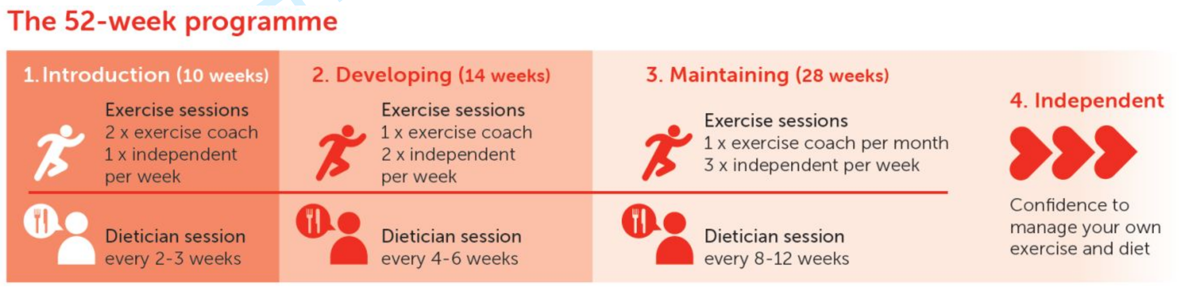

2. Exercise Component - supervised Sessions:

HIIT: Experienced trainers will lead supervised 1:1 exercise sessions incorporating high-intensity interval training (HIIT) based on the Norwegian 4x4 model[8]. This has been repeatedly used in patients with both coronary artery disease (CAD) and metabolic syndrome (MetS), and the evidence-base supports that HIIT is safe in appropriately screened patients[8–10]. At the start of each session, participants will be asked about any change in symptoms or medications and have blood pressure and heart rate measured.

The initial session will consist of a graduated, lead-in of moderate-intensity exercise prior to commencement of HIIT in subsequent sessions. Participants will be taught the Borg Rating of Perceived Exertion[11] (RPE; 6–20) to guide exercise intensity. HIIT will be performed on a static bike (or alternative cardiovascular equipment if unable to cycle) and will comprise a 3-minute warm-up; four 4-minute high-intensity intervals at RPE 15 (hard), finishing at RPE 17-18 (very hard); 3 minutes of active recovery at RPE 11-13 (somewhat hard) between each interval; and conclude with 3–5 minute recovery. Heart rate targets for HIIT (85 – 95% of heart-rate max) will be estimated with a baseline cardiopulmonary exercise test (CPET) to help gauge exercise intensity alongside RPE.

Trainers will help maintain target intensity and monitor participants throughout for concerning symptoms. Heart rate data, final RPE and duration for each high-intensity interval will be recorded for subsequent analysis. The exercise trainer will feedback data (e.g. total virtual distance cycled or power used) to participants as they work through the programme to highlight visible progress being achieved.

The goal as participants progress through the programme will be to gradually increase their overall workload (via watts or speed) on a weekly basis as they become fitter and stronger. To achieve this,

For peer review only - http://bmjopen.bmj.com/site/about/guidelines.xhtml

trainers will gradually increase the bike's resistance or participant's cycle speed, using heart rate response to ensure they remain in their target zone as workload increases.

Resistance Exercises: This has additional benefits, especially for blood pressure[12–14]. Sessions will therefore conclude with twenty minutes of resistance training.

This will be based on a short series of circuit training and will rotate muscle groups between sessions to allow recovery and maximise benefit. During the *introduction* phase trainers will utilise gym resources (free weights and weight machines) to build strength and confidence. In the subsequent developing phase this will switch to resistance against own body weight and bands to ensure patients learn routines that can then be undertaken in their own home in the maintaining phase and beyond.

The specific exercises are not directly prescribed to allow trainers to identify and respond to the individual needs of each participant. They will though be asked to follow this guiding framework and to record the exercises performed:

> *Introducing:* Many patients will be deconditioned, therefore initial sessions will begin with 10 – 15 repetitions at 40 – 50% of the 1 rep-max using free weights or weight machines. When ready (typically after 4 – 5 weeks), progress to 60 – 80% of the individual's 1 repetition maximum.
>
> *Developing & Maintaining:* Move to exercises against gravity / own body weight and use of exercise bands so patients should learn exercises they can start to use on their own. Circuits of 10 – 15 reps at 60 – 80% of the individual's 1 repetition maximum.

3. Exercise component – homework Sessions:

To enhance the weekly workload achieved and support long-term behaviour change, participants will be prescribed 'homework' exercise sessions. This will comprise moderate-intensity aerobic exercise (60 – 75% of heart-rate max) for 45-minutes in induction and consolidation phases (e.g., brisk walking), and increase to higher-intensity exercise (e.g., hill walking or jogging) in the maintenance phase. A once weekly resistance exercise session using workouts learnt in the supervised session during phases 1 and 2 will be added in phase 3. Participants will be provided with written guidance on effort levels, how to monitor their symptoms, and a heart-rate monitor (MyZone) to support self-directed exercise intensity at the appropriate level. These data will be reviewed at supervised exercise sessions to evaluate progress and provide support and kept for subsequent analysis.

4. Dietary component:

Nutrition therapy, as part of a comprehensive lifestyle intervention, improves and can reverse the components of MetS. The dietary intervention component comprises dietitian-led 1:1 consultations using evidence-based dietary advice for CAD-MetS patients[15–17]. This approach has been shown to improve blood pressure, insulin resistance and glycaemic control, lipid profile, systemic vascular inflammation and BMI[15,16,18–23].

For peer review only - http://bmjopen.bmj.com/site/about/guidelines.xhtml

Patients will complete pre-session combined photographic/written diet diaries and will be provided with an educational booklet incorporating the key dietary messages and structure. Recognising that there is no 'one-size-fits-all' pattern, the dietitian will work with participants to identify residual barriers to dietary change and potential solutions. The photographic diet diary will help highlight incremental areas for improvement in a straightforward manner. The dietitian will take a patient-centred approach to setting relevant short-term goals specific to the individual they are working with, which will be reviewed and built upon in each session.

The principles and guiding framework for the dietary advice will be:

- A focus on dietary patterns, including eating at regular mealtimes with portion control rather than "calorie counting", snack reduction and identifying healthy alternatives;
- Reduced refined carbohydrate intake, avoiding starch-based vegetables and refined grains;
- Reducing added sugars, making use of diet diaries to highlight 'hidden sugars' as well encouraging low glycaemic index foods in diabetic participants;
- Sensible drinking choices, recommending water over smoothies, diet drinks and juices, unsweetened tea/coffee and alcohol in moderation;
- Avoidance of ultra-processed foods, such as highly-processed meat and take-away meals;
- Choosing healthy sources of protein, encouraging plant-based foods such as pulses and nuts, regular oily fish, low-fat dairy products, eggs and lean unprocessed cuts of meat (if desired);
- Avoidance of 'low-fat' products, in favour of natural, whole foods with unsaturated and/or mono-saturated fats;
- Educate patients on the importance of swapping saturated fats for mono- and poly-unsaturated fats. Examples include identifying approaches to increasing natural, whole foods containing unsaturated fats, encouraging nuts, seeds, oily fish, avocado and extra-virgin olive oil;
- Increased dietary fibre, focusing on achieving this via diversifying vegetable intake, which encourages satiety, is good for gut and cardio-metabolic health and is good for blood pressure;
- Salt restriction.

Where indicated, dietitians will also continue to highlight the importance of smoking cessation.

5. Behavioural Support:

All practitioners will be required to use the following behavioural support tools in the delivery of Super Rehab.

➢ *Education*: Practitioners will reinforce the positive health outcomes that can be achieved with the suggested exercise and dietary changes the programme aims to deliver (i.e., helping patients to understand what to do, and why they are being asked to do it). This will build on their Super Rehab Booklet. Prescribed 'homework' exercises will be personalised to each individual participant, establishing how best they can incorporate this into their lifestyle.

➢ *Problem-solving:* Using the principles of a motivational interviewing, or person-centred approach, practitioners will support lifestyle changes by helping participants identify their own barriers or logistical issues to achieving their goals (e.g. money, family or work life, intolerances, time), and how they could overcome these. Example questions and techniques will be provided to trainers in their Super Rehab Manual.

For peer review only - http://bmjopen.bmj.com/site/about/guidelines.xhtml

- *Social Support*: Sustaining long term change is more likely when people have support from their own networks. Participants will be asked to identify their key social support (e.g. partner, friend, child), and how they could help as part of problem solving. Dietitians will be encouraged to recommend participants bring their key social support with them to dietary review sessions, and it will also be suggested that participants perform their homework exercise with them. This will help encourage and support participants, increasing the potential to maintain this lifestyle change in the longer term.

- *Goal-setting & Biofeedback:* All participants will also be set a series of longer-term goals for their involvement in the programme, typically:

a. ≥10% weight loss with a target BMI of 18.5 – 24.9 kg/m^2
b. A normal abdominal waist circumference (<80cm women / <94cm men)
c. Improvement in cardiovascular fitness.

However, during the in-programme review sessions practitioners will focus on the shorter-term, achievable, relevant goals related to the actions they can take and focus on between sessions that will help them achieve these longer-term goals. E.g., a specific change in food type in their diet rather than the broader outcome of weight loss. There will be follow-up on whether these goals have been met in subsequent sessions. Super Rehab practitioners will follow the *SMART* goal-setting principles:

Specific – set a clear target/change for them to make (what, when & where)

Measurable – ensure it can be assessed at future visits

Achievable – ensure the goal is something you (and they) believe they can do

Relevant – select goals that ensure a step-wise improvement

Time-bound – set a realistic time-frame for them to achieve the goal(s) by

Changes in body metrics such as weight and abdominal waist circumference will be used within sessions to feedback on performance – both for positive re-enforcement and to supplement goal-setting. The 1:1 nature of the sessions will be used to develop rapport, engender a working relationship and a level of accountability on the participant to deliver the changes asked of them.

For peer review only - http://bmjopen.bmj.com/site/about/guidelines.xhtml

6. References:

1. Chauhan BF, Jeyaraman M, Mann AS, Lys J, Skidmore B, Sibley KM, Abou-Setta A, Zarychanksi R. Behavior change interventions and policies influencing primary healthcare professionals' practice-an overview of reviews. *Implementation Science*. 2017;12(1).

2. Garber AJ, Abrahamson MJ, Barzilay JI, Blonde L, Bloomgarden ZT, Bush MA, Dagogo-Jack S, DeFronzo RA, Einhorn D, Fonseca VA, Garber JR, Garvey WT, Grunberger G, Handelsman Y, Hirsch IB, et al. Consensus statement by the American association of clinical endocrinologists and American college of endocrinology on the comprehensive type 2 diabetes management algorithm - 2018 executive summary. *Endocrine Practice*. 2018;24(1):91–120.

3. Pérez EA, González MP, Martínez-Espinosa RM, Vila MDM, García-Galbis MR. Practical guidance for interventions in adults with metabolic syndrome: Diet and exercise vs. changes in body composition. *International Journal of Environmental Research and Public Health*. 2019;16(18).

4. Singh N, Stewart RAH, Benatar JR. Intensity and duration of lifestyle interventions for long-term weight loss and association with mortality: A meta-analysis of randomised trials. *BMJ Open*. 2019;9(8):1–10.

5. Dutton, GR, Laitner, MH, Perri M. Lifestyle Interventions for Cardiovascular Disease Risk Reduction: A Systematic Review of the Effects of Diet Composition, Food Provision, and Treatment Modality on Weight Loss. *Curr Atheroscler Rep*. 2014;16(10):442.

6. Samdal GB, Eide GE, Barth T, Williams G, Meland E. Effective behaviour change techniques for physical activity and healthy eating in overweight and obese adults; systematic review and meta-regression analyses. *International Journal of Behavioral Nutrition and Physical Activity*. 2017;14(1):1–14.

7. Greaves CJ, Sheppard KE, Abraham C, Hardeman W, Roden M, Evans PH, Schwarz P. Systematic review of reviews of intervention components associated with increased effectiveness in dietary and physical activity interventions. *BMC Public Health*. 2011;11(1):119.

8. Taylor JL, Holland DJ, Spathis JG, Beetham KS, Wisløff U, Keating SE, Coombes JS. Guidelines for the delivery and monitoring of high intensity interval training in clinical populations. *Progress in Cardiovascular Diseases*. 2019;62(2):140–146.

9. Wewege MA, Ahn D, Yu J, Liou K, Keech A. High-intensity interval training for patients with cardiovascular disease-is it safe? A systematic review. *Journal of the American Heart Association*. 2018;7(21):1–19.

10. Swain D. Vigorous intensity exercise is essentially safe for coronary heart disease patients. *Evidence based medicine*. 2013;18(4):159–160.

11. Borg GA. Psychophysical bases of perceived exertion. *Medicine and science in sports and exercise*. 1982;14(5):377–81.

12. Ihalainen JK, Inglis A, Mäkinen T, Newton RU, Kainulainen H, Kyröläinen H, Walker S. Strength training improves metabolic health markers in older individual regardless of training frequency. *Frontiers in Physiology*. 2019;10(FEB):1–12.

13. Braith RW, Stewart KJ. Resistance exercise training: Its role in the prevention of cardiovascular disease. *Circulation*. 2006;113(22):2642–2650.

For peer review only - http://bmjopen.bmj.com/site/about/guidelines.xhtml

14. Schroeder EC, Franke WD, Sharp RL, Lee D chul. Comparative effectiveness of aerobic, resistance, and combined training on cardiovascular disease risk factors: A randomized controlled trial. *PLoS ONE*. 2019;14(1):1–14.

15. Unwin DJ, Tobin SD, Murray SW, Delon C, Brady AJ. Substantial and sustained improvements in blood pressure, weight and lipid profiles from a carbohydrate restricted diet: An observational study of insulin resistant patients in primary care. *International Journal of Environmental Research and Public Health*. 2019;16(15).

16. Hoyas I, Leon-Sanz M. Nutritional Challenges in Metabolic Syndrome. *Journal of Clinical Medicine*. 2019;8(9):1301.

17. Mach F, Baigent C, Catapano AL, Koskinas KC, Casula M, Badimon L, Chapman MJ, De Backer GG, Delgado V, Ference BA, Graham IM, Halliday A, Landmesser U, Mihaylova B, Pedersen TR, et al. 2019 ESC/EAS Guidelines for the management of dyslipidaemias: Lipid modification to reduce cardiovascular risk. *European Heart Journal*. 2020;41(1):111–188.

18. Dong T, Guo M, Zhang P, Sun G, Chen B. The effects of low-carbohydrate diets on cardiovascular risk factors: A meta-analysis. *PLoS ONE*. 2020;15(1):1–16.

19. DiNicolantonio JJ, OKeefe JH. Added sugars drive coronary heart disease via insulin resistance and hyperinsulinaemia: a new paradigm. *Open Heart*. 2017;4(2):e000729.

20. Threapleton DE, Greenwood DC, Evans CEL, Cleghorn CL, Nykjaer C, Woodhead C, Cade JE, Gale CP, Burley VJ. Dietary fibre intake and risk of cardiovascular disease: Systematic review and meta-analysis. *BMJ (Online)*. 2013;347(December):1–12.

21. Forouhi NG, Krauss RM, Taubes G, Willett W. Dietary fat and cardiometabolic health: Evidence, controversies, and consensus for guidance. *BMJ (Online)*. 2018;361(June):1–8.

22. Brandhorst S, Longo VD. Dietary Restrictions and Nutrition in the Prevention and Treatment of Cardiovascular Disease. *Circulation Research*. 2019;124(6):952–965.

23. Korakas E, Dimitriadis G, Raptis A, Lambadiari V. Dietary composition and cardiovascular risk: A mediator or a Bystander? *Nutrients*. 2018;10(12).

For peer review only

Reporting checklist for protocol of a clinical trial.

Based on the SPIRIT guidelines.

		Reporting Item	Page Number
**Administrative information**			
Title	#1	Descriptive title identifying the study design, population, interventions, and, if applicable, trial acronym	1
Trial registration	#2a	Trial identifier and registry name. If not yet registered, name of intended registry	4
Trial registration: data set	#2b	All items from the World Health Organization Trial Registration Data Set	N/a
Protocol version	#3	Date and version identifier	N/a
Funding	#4	Sources and types of financial, material, and other support	27-28
Roles and responsibilities: contributorship	#5a	Names, affiliations, and roles of protocol contributors	1, 27
Roles and responsibilities:	#5b	Name and contact information for the trial sponsor	27

For peer review only

sponsor contact information			
Roles and responsibilities: sponsor and funder	#5c	Role of study sponsor and funders, if any, in study design; collection, management, analysis, and interpretation of data; writing of the report; and the decision to submit the report for publication, including whether they will have ultimate authority over any of these activities	N/a
Roles and responsibilities: committees	#5d	Composition, roles, and responsibilities of the coordinating centre, steering committee, endpoint adjudication committee, data management team, and other individuals or groups overseeing the trial, if applicable (see Item 21a for data monitoring committee)	24-25
**Introduction**			
Background and rationale	#6a	Description of research question and justification for undertaking the trial, including summary of relevant studies (published and unpublished) examining benefits and harms for each intervention	5-7
Background and rationale: choice of comparators	#6b	Explanation for choice of comparators	7
Objectives	#7	Specific objectives or hypotheses	8-9

For peer review only

Trial design	#8	Description of trial design including type of trial (eg, parallel group, crossover, factorial, single group), allocation ratio, and framework (eg, superiority, equivalence, non-inferiority, exploratory)	8
**Methods: Participants, interventions, and outcomes**			
Study setting	#9	Description of study settings (eg, community clinic, academic hospital) and list of countries where data will be collected. Reference to where list of study sites can be obtained	8 & 11
Eligibility criteria	#10	Inclusion and exclusion criteria for participants. If applicable, eligibility criteria for study centres and individuals who will perform the interventions (eg, surgeons, psychotherapists)	11-13
Interventions: description	#11a	Interventions for each group with sufficient detail to allow replication, including how and when they will be administered	18-20
Interventions: modifications	#11b	Criteria for discontinuing or modifying allocated interventions for a given trial participant (eg, drug dose change in response to harms, participant request, or improving / worsening disease)	N/a

For peer review only

Interventions: adherance	#11c	Strategies to improve adherence to intervention protocols, and any procedures for monitoring adherence (eg, drug tablet return; laboratory tests)	N/a
Interventions: concomitant care	#11d	Relevant concomitant care and interventions that are permitted or prohibited during the trial	N/a
Outcomes	#12	Primary, secondary, and other outcomes, including the specific measurement variable (eg, systolic blood pressure), analysis metric (eg, change from baseline, final value, time to event), method of aggregation (eg, median, proportion), and time point for each outcome. Explanation of the clinical relevance of chosen efficacy and harm outcomes is strongly recommended	8-9 & 14-16
Participant timeline	#13	Time schedule of enrolment, interventions (including any run-ins and washouts), assessments, and visits for participants. A schematic diagram is highly recommended (see Figure)	10
Sample size	#14	Estimated number of participants needed to achieve study objectives and how it was determined, including clinical and statistical assumptions supporting any sample size calculations	13-14
Recruitment	#15	Strategies for achieving adequate participant enrolment to reach target sample size	13-14

For peer review only

Methods: Assignment of interventions (for controlled trials)

Allocation: sequence generation	#16a	Method of generating the allocation sequence (eg, computer-generated random numbers), and list of any factors for stratification. To reduce predictability of a random sequence, details of any planned restriction (eg, blocking) should be provided in a separate document that is unavailable to those who enrol participants or assign interventions	14
Allocation concealment mechanism	#16b	Mechanism of implementing the allocation sequence (eg, central telephone; sequentially numbered, opaque, sealed envelopes), describing any steps to conceal the sequence until interventions are assigned	14
Allocation: implementation	#16c	Who will generate the allocation sequence, who will enrol participants, and who will assign participants to interventions	N/a
Blinding (masking)	#17a	Who will be blinded after assignment to interventions (eg, trial participants, care providers, outcome assessors, data analysts), and how	22
Blinding (masking): emergency unblinding	#17b	If blinded, circumstances under which unblinding is permissible, and procedure for revealing a participant's allocated intervention during the trial	22

For peer review only

**Methods: Data collection, management, and analysis**			
Data collection plan	#18a	Plans for assessment and collection of outcome, baseline, and other trial data, including any related processes to promote data quality (eg, duplicate measurements, training of assessors) and a description of study instruments (eg, questionnaires, laboratory tests) along with their reliability and validity, if known. Reference to where data collection forms can be found, if not in the protocol	14-16, 18, & 20-22
Data collection plan: retention	#18b	Plans to promote participant retention and complete follow-up, including list of any outcome data to be collected for participants who discontinue or deviate from intervention protocols	21, 25
Data management	#19	Plans for data entry, coding, security, and storage, including any related processes to promote data quality (eg, double data entry; range checks for data values). Reference to where details of data management procedures can be found, if not in the protocol	21-22
Statistics: outcomes	#20a	Statistical methods for analysing primary and secondary outcomes. Reference to where other details of the	23

For peer review only

		statistical analysis plan can be found, if not in the protocol	
Statistics: additional analyses	#20b	Methods for any additional analyses (eg, subgroup and adjusted analyses)	N/a
Statistics: analysis population and missing data	#20c	Definition of analysis population relating to protocol non-adherence (eg, as randomised analysis), and any statistical methods to handle missing data (eg, multiple imputation)	N/a

Methods: Monitoring

Data monitoring: formal committee	#21a	Composition of data monitoring committee (DMC); summary of its role and reporting structure; statement of whether it is independent from the sponsor and competing interests; and reference to where further details about its charter can be found, if not in the protocol. Alternatively, an explanation of why a DMC is not needed	24-25
Data monitoring: interim analysis	#21b	Description of any interim analyses and stopping guidelines, including who will have access to these interim results and make the final decision to terminate the trial	N/a
Harms	#22	Plans for collecting, assessing, reporting, and managing solicited and spontaneously reported adverse events and other unintended effects of trial interventions or trial conduct	25

For peer review only

Auditing	#23	Frequency and procedures for auditing trial conduct, if any, and whether the process will be independent from investigators and the sponsor	N/a
**Ethics and dissemination**			
Research ethics approval	#24	Plans for seeking research ethics committee / institutional review board (REC / IRB) approval	3 & 25-26
Protocol amendments	#25	Plans for communicating important protocol modifications (eg, changes to eligibility criteria, outcomes, analyses) to relevant parties (eg, investigators, REC / IRBs, trial participants, trial registries, journals, regulators)	13
Consent or assent	#26a	Who will obtain informed consent or assent from potential trial participants or authorised surrogates, and how (see Item 32)	14
Consent or assent: ancillary studies	#26b	Additional consent provisions for collection and use of participant data and biological specimens in ancillary studies, if applicable	N/a
Confidentiality	#27	How personal information about potential and enrolled participants will be collected, shared, and maintained in order to protect confidentiality before, during, and after the trial	21-22

For peer review only

Declaration of interests	#28	Financial and other competing interests for principal investigators for the overall trial and each study site	27
Data access	#29	Statement of who will have access to the final trial dataset, and disclosure of contractual agreements that limit such access for investigators	N/a
Ancillary and post trial care	#30	Provisions, if any, for ancillary and post-trial care, and for compensation to those who suffer harm from trial participation	N/a
Dissemination policy: trial results	#31a	Plans for investigators and sponsor to communicate trial results to participants, healthcare professionals, the public, and other relevant groups (eg, via publication, reporting in results databases, or other data sharing arrangements), including any publication restrictions	25
Dissemination policy: authorship	#31b	Authorship eligibility guidelines and any intended use of professional writers	N/a
Dissemination policy: reproducible research	#31c	Plans, if any, for granting public access to the full protocol, participant-level dataset, and statistical code	N/a

Appendices

Informed consent materials	#32	Model consent form and other related documentation given to participants and authorised surrogates	N/a
Biological specimens	#33	Plans for collection, laboratory evaluation, and storage of biological specimens for genetic or molecular analysis in	N/a

For peer review only

the current trial and for future use in ancillary studies, if applicable

None The SPIRIT Explanation and Elaboration paper is distributed under the terms of the Creative Commons Attribution License CC-BY-NC. This checklist can be completed online using https://www.goodreports.org/, a tool made by the EQUATOR Network in collaboration with Penelope.ai

For peer review only

BMJ Open

“Super Rehab”: can we achieve coronary artery disease regression? (a feasibility study) – a study protocol

Journal:	*BMJ Open*
Manuscript ID	bmjopen-2023-080735.R1
Article Type:	Protocol
Date Submitted by the Author:	24-Oct-2023
Complete List of Authors:	Graby, John; Royal United Hospitals Bath NHS Foundation Trust, Cardiology; University of Bath, Health<br>Khavandi, Ali; Royal United Hospitals Bath NHS Foundation Trust, Cardiology<br>Gillison, Fiona; University of Bath, Health<br>Smith, Theresa; University of Bath, Mathematics<br>Murphy, David; Royal United Hospitals Bath NHS Foundation Trust, Cardiology; University of Bath, Health<br>Peacock, Oliver; University of Bath, Department for Health<br>McLeod, Hugh; University of Bristol<br>Dastidar, Amardeep; North Bristol NHS Trust, Cardiology<br>Antoniades, Charalambos; University of Oxford Radcliffe Department of Medicine,<br>Thompson, Dylan; University of Bath, Health<br>Rodrigues, Jonathan; Royal United Hospitals Bath NHS Foundation Trust, Radiology; University of Bath, Health
**Primary Subject Heading**:	Cardiovascular medicine
Secondary Subject Heading:	Cardiovascular medicine, Patient-centred medicine, Rehabilitation medicine, Research methods
Keywords:	Coronary heart disease < CARDIOLOGY, Cardiovascular imaging < RADIOLOGY & IMAGING, Adult cardiology < CARDIOLOGY, Rehabilitation medicine < INTERNAL MEDICINE

SCHOLARONE™
Manuscripts

Title: "Super Rehab": can we achieve coronary artery disease regression? (a feasibility study) – a study protocol

Authors:

John Graby*[1,2], Ali Khavandi*[1], Fiona Gillison[3], Theresa Smith[4], David Murphy[1,2], Oliver Peacock[2], Hugh McLeod[5,6], Amardeep Dastidar[7], Charalambos Antoniades[8], Dylan Thompson[+2], Jonathan CL Rodrigues[+2,9]

*Denotes joint first authors, [+]denotes joint last authors.

1. Department of Cardiology, Royal United Hospitals Bath NHS Foundation Trust, Combe Park, Bath, Avon, BA1 3NG, UK.
2. Centre for Nutrition, Exercise and Metabolism (CNEM), Department for Health, University of Bath, Claverton Down, Bath, BA2 7AY, UK.
3. Centre for Motivation and Health Behaviour Change, Department for Health, University of Bath, Claverton Down, Bath, BA2 7AY, UK.
4. Department of Mathematical Sciences, University of Bath, Claverton Down, Bath, BA2 7AY, UK.
5. Population Health Sciences, Bristol Medical School, University of Bristol, BS8 1UD, UK.
6. The National Institute for Health Research Applied Research Collaboration West (NIHR ARC West) at University Hospitals Bristol and Weston NHS Foundation Trust, Bristol BS1 2NT, UK.
7. Department of Cardiology, North Bristol NHS Trust, Southmead Road, Bristol, BS10 5NB, UK.
8. Acute Multidisciplinary Imaging & Interventional Centre, University of Oxford, John Radcliffe Hospital, Oxford, OX3 9DU, UK.
9. Department of Radiology, Royal United Hospitals Bath NHS Foundation Trust, Combe Park, Bath, Avon, BA1 3NG, UK.

For peer review only - http://bmjopen.bmj.com/site/about/guidelines.xhtml

For peer review only

Corresponding authors:

Dr John Graby

Email: john.graby@nhs.net

Address: Department for Health, University of Bath, Claverton Down, Bath, BA2 7AY, UK.

ORCID iDs

John Graby: 0000-0002-8753-1343

Ali Khavandi: 0000-0001-6374-3782

Fiona Gillison: 0000-0002-6461-7638

Theresa Smith: 0000-0002-7085-3864

David Murphy: 0000-0003-0670-2567

Oliver Peacock: 0000-0002-2435-2903

Hugh McLeod: 0000-0002-2266-7303

Amardeep Dastidar: 0000-0002-8688-5326

Charalambos Antoniades: 0000-0002-6983-5423

Dylan Thompson: 0000-0002-6312-1518

Jonathan CL Rodrigues: 0000-0003-2744-3509

For peer review only

Abstract:

Introduction:

Patients diagnosed with coronary artery disease (CAD) are currently treated with medications and lifestyle advice to reduce the likelihood of disease progression and risk of future major adverse cardiovascular events (MACE). Where obstructive disease is diagnosed, revascularisation may be considered to treat refractory symptoms. However, many patients with co-existent cardiovascular risk factors, particularly those with metabolic syndrome (MetS), remain at heightened risk of future MACE despite current management.

Cardiac Rehabilitation is offered to patients post-revascularisation, however, there is no definitive evidence demonstrating its benefit in a primary prevention setting. We propose that an intensive lifestyle intervention (Super Rehab, SR) incorporating high-intensity exercise, diet and behavioural change techniques may improve symptoms, outcomes, and enable CAD regression.

This study aims to examine the feasibility of delivering a multi-centre randomised controlled trial (RCT) testing SR for patients with CAD, in a primary prevention setting.

Methods and analysis:

This is a multi-centre randomised controlled feasibility study of SR vs usual care in patients with CAD. The study aims to recruit 50 participants aged 18-75 across two centres. Feasibility will be assessed against rates of recruitment, retention and, in the intervention arm, attendance and adherence to SR. Qualitative interviews will explore trial experiences of study participants and practitioners. Variance of change in CAD across both arms of the study (assessed with serial CT coronary angiography) will inform the design and power of a future, multi-centre RCT.

Ethics and dissemination:

Ethics approval was granted by South West – Frenchay Research Ethics Committee (reference: 21/SW/0153, 18/01/2022). Study findings will be disseminated via presentations to relevant

For peer review only - http://bmjopen.bmj.com/site/about/guidelines.xhtml

stakeholders, national and international conferences and open-access peer-reviewed research publications.

Trial registration number ISRCTN14603929

Strengths and limitations of the study:

1. This is a multi-centre randomised controlled feasibility study.
2. This study will use a mixed methods approach, collecting quantitative and qualitative data, assessing a novel rehab programme (Super Rehab).
3. This study is not designed to determine efficacy or cost-effectiveness and is limited to informing the feasibility of a larger trial of Super Rehab.
4. Contemporary CT measurements of coronary artery inflammation and plaque will be acquired.
5. If it is determined that a larger, multi-centre trial is feasible, this study will provide key data to improve its design.

For peer review only

Manuscript

Introduction:

Coronary artery disease (CAD) remains the most common cause of premature death in the UK and its treatment consumes substantial health-care resources. Overall plaque burden has recently been shown to be a more sensitive marker for risk of future adverse cardiovascular event than luminal stenosis, which has historically been the focus in CAD treatment[1]. Plaque regression, not just stabilisation, is a novel end-point in the treatment of CAD and has been used in pharmaceutical trials[2-3]. Disease regression is the target in several other disease modalities where previously it was not thought possible, e.g. type II diabetes[4].

The role of poor diet and physical inactivity is well recognised in the pathogenesis of CAD[5]. Lifestyle intervention studies focusing on physical activity and diet improve cardiovascular fitness (which reduces cardiovascular risk), metabolic parameters, and psychological and self-perceived health[6-8]. Exercise training increases angina-free activity threshold, attenuates disease progression and improves event-free survival[9]. Lifestyle intervention reduces systemic inflammation[10].

A limited number of small studies combining a lifestyle intervention with routine care have shown the potential for CAD regression[11-13], indicating both a preventative and therapeutic impact[14]. Plaque regression has been observed at 12-months and complete plaque regression has been demonstrated in case reports of patients with significant disease without coronary intervention (e.g. stents)[15]. However, the current evidence-base is of insufficient quality to justify a shift to healthcare delivered preventative programmes in a chronic coronary syndrome (CCS) population (i.e. established, stable CAD). Such a shift in practice requires evidence from rigorous, adequately powered, randomised controlled trials (RCT) with suitable endpoints to persuade both commissioners and healthcare professionals that this clinical pathway is cost-effective.

For peer review only - http://bmjopen.bmj.com/site/about/guidelines.xhtml

A healthcare delivered lifestyle intervention, cardiac rehabilitation (CR), exists for patients further down the CAD pathway who have suffered a MI or required coronary revascularisation with stents or bypass surgery. A Cochrane Review reported that whilst exercise-based CR appears to improve cardiovascular mortality, further research is required in higher-risk CCS groups in a primary prevention setting[9, 16]. This is despite >50% of MI resulting from non-obstructive plaque, highlighting the importance of robust treatments for this patient population[17].

Super Rehab (SR) has been designed to reflect the evolution in lifestyle intervention practices since the advent of traditional CR, incorporating the balance of recent evidence, the likelihood of patient adherence and cost benefits. Specifically SR is a cardiology-led intervention incorporating high-intensity interval training (HIIT), updated best-practice dietary advice and current behavioural change techniques.

Exercise: HIIT, which comprises alternating periods of high intensity aerobic exercise with light intensity recovery periods, has additional benefits over conventional moderate-intensity exercise incorporated into CR[18-20]. Aerobic capacity (VO_2 max), is an independent predictor of all-cause mortality and cardiovascular prognosis[6]. HIIT achieves a greater improvement in VO_2 max, improves outcomes in CAD patients[18] and is supported by robust safety data[21]. SR incorporates a modified version of the Norwegian 4x4 minute HIIT model, which has been received well by CAD patients[22].

Diet: SR integrates best-practice dietary advice for atherosclerotic cardiovascular disease and CAD patients, which has evolved to incorporate reduced refined carbohydrate and sugar content, and healthier fats and proteins. Evidence has demonstrated additional improvements in cardiovascular risk, atherosclerotic burden, systemic inflammation, glucose and blood pressure control[23-26].

For peer review only - http://bmjopen.bmj.com/site/about/guidelines.xhtml

Delivery: Traditional CR pathways have well-documented low attendance rates, consistently <50%[27]. To improve compliance and adherence, SR adopts evidence-based strategic behavioural intervention techniques to enhance the potential for patients to adopt and sustain changes promoted during SR[28-29]. This includes techniques that promote motivation, self-regulation and support[30] and an autonomy supportive (patient-centred) delivery style[31].

CT coronary angiography (CTCA) is now the mainstay investigation in new onset stable chest pain of suspected cardiac origin. CTCA provides an excellent, non-invasive tool for tracking change in coronary plaque[32-33], whilst radiation doses are low and reducing[34]. Imaging techniques now enable the assessment of additional measures that are highly predictive of future risk. This includes: (1) *Pericoronary fat attenuation index* (FAI) – a sensitive CT-derived biomarker of coronary inflammation that out-performs all other cardiovascular disease risk-stratifications[35-36]; (2) *High-risk plaque features* – morphological markers of vulnerability based on the composition and location, which predict MI risk[37]; and (3) *Fractional flow reserve* (FFR_{CT}) – a computational fluid dynamics algorithm that non-invasively estimates flow change across a plaque to determine likelihood of obstructive disease, with adverse haemodynamics denoting risk of future MI[38].

We hypothesise that SR will improve symptoms and cause CAD regression, reducing the risk of MI and death. To change current clinical practice and integrate SR into standard care, a suitably-powered RCT is required to establish efficacy using CAD-specific prognostic clinical outcomes, tracked via changes on CTCA. The present study aims to examine the feasibility of delivering a subsequent multi-centre RCT.

For peer review only - http://bmjopen.bmj.com/site/about/guidelines.xhtml

Methods and analysis:

Study design

A multi-centre two parallel-arm feasibility study randomising 50 patients (1:1) to Usual Care or SR plus Usual Care to establish the feasibility of conducting a subsequent, larger and powered multi-centre RCT. Figure 1 provides a study flow-chart and the protocol follows the Standard Protocol Items: Recommendations for Interventional Trials reporting guidelines[39].

Study aim

To establish the feasibility of delivering and evaluating a definitive RCT of SR with Usual Care vs Usual Care only in the target population.

Study objectives

As a feasibility study, the primary objective is to assess the feasibility of delivering the proposed study design testing the efficacy of SR. Feasibility criteria assessed include:

1. Recruitment rates: the proportion of eligible patients who accept the invitation to participate.
2. Retention and adherence: the proportion of participants who complete the study and, for those in the intervention group, the proportion of offered sessions completed.
3. Acceptability of the intervention, study design and outcome measures as well as participants' and practitioners' experiences of SR.

Additional secondary objectives include:

4. Evaluate and refine data-collection procedures and outcomes measures.

For peer review only - http://bmjopen.bmj.com/site/about/guidelines.xhtml

5. Pilot the use of routine clinical data (body mass index [BMI] and HbA1c) in identifying CAD patients with MetS.

6. Obtain preliminary data for CAD regression to inform power calculations for a subsequent RCT.

Study population and setting

Participants will be prospectively identified via routine clinically indicated CTCA lists at Royal United Hospitals Bath NHS Foundation Trust and North Bristol Trust. In line with the National Institute for Health and Care Excellence (NICE) guidelines on the assessment of patients with stable chest pain[40], patients with new-onset chest pain and no prior history of CAD are referred for a CTCA to assess for the presence and severity of CAD. This scan will be incorporated into screening and outcome assessments.

Patients with CAD who also have metabolic syndrome (MetS) are a particularly high-risk CAD group, representing up to half of all CAD patients[41] and have a 3-fold increased risk of death[42],[43]. MetS is a cluster of conditions including abdominal obesity, raised triglycerides, high-density lipoprotein (HDL-cholesterol) or fasting glucose, and hypertension[44]. MetS not only increases the risk of CAD, but predicts more severe CAD and worse prognosis[45-46]. Inflammation is a potent driver of atherosclerosis[47], risk of myocardial infarction (MI)[48-49] as well as disease progression in MetS[50]. Thus, CAD-MetS patients with evidence of inflammation are an important cohort with notable residual risk despite current treatments. A targeted rehabilitation programme may offer a significant benefit to this sizeable patient group.

For peer review only

Inclusion criteria

1. Aged 18–75 years at consent;
2. CTCA demonstrates CAD with plaque causing a stenosis of ≥25% in ≥1 coronary artery;
3. Evidence of coronary inflammation (FAI > -70.1HU or FAI-score [relative to age- and sex-matched patients] ≥75th percentile in the left anterior descending or right coronary artery or ≥90th in the circumflex[35]);
4. MetS, defined as ≥3 of the following within 6 months of CTCA[44]:
   - High abdominal waist circumference (≥94cm males, ≥80cm females);
   - Hypertension (≥130/85mmHg or on treatment);
   - Diabetes mellitus or raised fasting glucose or HbA1c (glucose ≥5.6mmol/L, HbA1c ≥42mmol/L);
   - Reduced high-density lipoprotein (≤1mmol/L males, <1.3mmol/L females);
   - Raised triglycerides (>1.7mmol/L).

Exclusion criteria

Participants will not be eligible if they have conditions that preclude high-intensity exercise, including:

1. CAD requiring revascularisation;
2. Unstable angina;
3. New York Heart Association class III/IV heart failure or severe left ventricular impairment;
4. Severe valve disease;
5. Significant cardiomyopathy (as assessed by a Cardiologist);
6. Severe hypertension (defined as blood pressure >180/120mmHg);
7. Uncontrolled cardiac arrhythmia;

For peer review only - http://bmjopen.bmj.com/site/about/guidelines.xhtml

8. Prior aortic dissection;
9. Recent acute pulmonary embolus, deep vein thrombosis, stroke or transient ischaemic attack;
10. Severe autonomic or peripheral neuropathy;
11. Acute systemic illness or fever;
12. Significant acute or chronic renal failure;
13. Pulmonary fibrosis or interstitial lung disease;
14. Physically unable to participate in exercise;
15. Clinically significant ECG abnormality at the screening visit, which, in the opinion of a Cardiologist, exposes the subject to risk by enrolling in the trial.

Additionally, patients with a history of prior MI or coronary re-vascularisation, severe coronary calcification that precludes assessment of the coronary lumen on CTCA, current participation in another intervention-based study, and pregnant or breastfeeding individuals will be excluded.

Amendments to eligibility criteria

During the initial recruitment period, it became clear that the original eligibility criteria (high BMI [>28kg/m^2] and abnormal glucose control [HbA1c >42mmol/mol or >6%]) was incompletely capturing patients with MetS. Thus, a substantial amendment was submitted to the ethics committee to allow recruitment of participants who met the international consensus criteria for MetS (outlined in the inclusion criteria above)[44]. The FAI criterion was also adjusted to ensure younger patients with high inflammation relative to age- and sex-matched controls (FAI-score) were captured, as well as patients recruited based on their absolute FAI value (approved 9/5/2022).

A further substantial amendment was submitted to the ethics committee to ensure participants with a raised HbA1c could participate, as fasting glucose levels were incompletely

For peer review only - http://bmjopen.bmj.com/site/about/guidelines.xhtml

capturing patients with poor longer-term glucose control (approved 12/7/2022). A non-substantial amendment clarified that participants needed to have met eligibility criteria within 6-months of their CTCA (approved 20/9/2022).

Sample size

As a feasibility study there is no primary outcome measure to inform a power calculation. However, a sample size of 25 in each arm is sufficient to estimate feasibility outcome proportions for acceptability rates of 64% (with 90% confidence interval [CI] 46-80%) and adherence rates of 84% (90% CI 67-93%). The corresponding intervals for recruitment rate will be more precise as the number of potential participants will be >50.

Screening and consent

Initial pre-screening identifies potential participants who meet CAD inclusion criteria on their clinically indicated CTCA. Potential participants will be informed of the experimental protocol in writing and invited for a clinic assessment where they will complete screening with a clinician. Eligible and willing participants will be asked for written informed consent obtained according to Good Clinical Practice guidelines, and Usual Care will be instituted.

Randomisation

Following completion of all baseline assessments, participants will be block randomised (1:1) to SR and Usual Care or Usual Care only. Randomisation will be performed using a web-based platform (Sealed Envelope: https://www.sealedenvelope.com/) in permuted blocks of four with stratification by sex using an allocation sequence generated at random.

For peer review only - http://bmjopen.bmj.com/site/about/guidelines.xhtml

Assessments (see Figure 2)

Participant self-reported data will be collected at baseline, 6-months, 12-months and 15-months (post-randomisation):

1. Demographics (baseline only).

2. The EQ-5D-5L (EuroQol-5 dimensions-5 levels) questionnaire, a validated generic patient-reported outcome measure[51].

3. Symptom assessment with the Seattle Angina Questionnaire.

4. Psychological well-being assessment with the Hospital Anxiety and Depression Scale (HADS).

5. Holistic capability assessment with the ICEpop CAPability measure for Adults (ICECAP-A)[52].

6. NHS resource use via a study specific questionnaire.

The following clinical data will be collected baseline, 6, 12 and 15-month time-points:

7. Anthropometrics, including height, weight, BMI, abdominal waist circumference.

8. Body composition, assessed with dual-energy X-ray absorptiometry (DEXA).

9. 7-day blood pressure diary.

10. Cardiopulmonary fitness assessed by cardiopulmonary exercise testing (CPET).

The following clinical data will be collected at 0, 6 and 12-months only:

11. CT coronary angiography, enabling analysis of luminal stenosis, plaque volume (sub-divided by composition), FAI and coronary flow change (assessed by CT-derived fractional flow reserve [FFR_{CT}]).

12. Fasting blood tests measuring full lipid profile, glucose, glycated hemoglobin (HbA1c) and inflammatory markers (e.g. high-sensitivity C-reactive protein).

13. Cardiac structural and functional assessment with echocardiography.

For peer review only - http://bmjopen.bmj.com/site/about/guidelines.xhtml

The following free-living data will be collected at baseline, 6, 12 and 15-month time points:

14. 7-day photographic and/or written diet diary, to track change in dietary patterns over time.

15. 7-day physical activity monitor (GENEActiv) to characterise free-living physical activity.

In line with the objectives of a feasibility study, we will also gather information on:

16. Recruitment and dropout rates.

17. Adherence to SR, assessed via attendance, maximum rating of perceived exertion (RPE) during HIIT, and proportion of participants opting for virtual dietary sessions.

18. A qualitative assessment of trial procedures and SR acceptability via interim and end-of study interviews with participants and practitioners.

Cardiopulmonary exercise testing (CPET)

Participants in both groups will undergo a symptom-limited CPET to determine their VO_2peak and maximum heart-rate, which will guide exercise intensity parameters for SR HIIT sessions. The CPET will be conducted on a cycle ergometer, beginning with a 5-minute warm-up against zero resistance. A subsequent ramp progression is used with resistance increasing by 10-20 watts-per-minute (individualised to participant) aiming for a total duration of 8-12 minutes[53]. The test discontinues at volitional exhaustion targeting a Borg rating of perceived exertion (RPE) of ≥17. A subsequent recovery phase lasts ≥3 minutes against zero resistance. Achievement of VO_2peak will be assumed if the following criteria are met[53]:

1. Respiratory exchange ratio ≥1.10;

   or

2. Increment in $VO_2 \leq 5$ mL/kg^{-1}.min^{-1} in response to increasing gradient.

   and

For peer review only - http://bmjopen.bmj.com/site/about/guidelines.xhtml

3. RPE ≥17;

Participants unable to complete the CPET due to a health problem raised during the exercise test or concerning discomfort exercising will discontinue involvement in the study on safety grounds. Participants who achieve an RPE <19 and a maximum heart-rate <80% of their age-predicted will be invited for a repeat test to confirm the accuracy of results.

Control Arm: Usual Care

All patients will be offered guideline-based recommended doses of aspirin and statin, unless contra-indicated[54-55]. Participants' General Practitioners will be provided with a study information sheet and encouraged to adjust medications as needed according to national guidelines (e.g. for hypertension) if conditions change whilst participating in the study. Participants will also be provided routine, verbal one-off lifestyle advice in line with NICE guidance, recommending 150 minutes of moderate intensity exercise per week, encouraging a healthy, balanced diet, weight loss, reducing alcohol consumption and smoking cessation.

Intervention Arm: Super Rehab (plus Usual Care)

A detailed description of the SR protocol is provided in the supplementary material. In short, SR is designed to enable and support successful behavioural change with a predominant focus on exercise and diet, with all sessions delivered 1-to-1. The programme will be explicitly introduced by a Cardiologist, ensuring the programme is presented as a meaningful intervention to improve health outcomes. The Cardiologist will introduce the concepts involved in the nutritional advice, provide advice and oversight to both the dietitian and exercise trainers, and conduct a further clinical review at the end of each intervention phase.

For peer review only

For peer review only - http://bmjopen.bmj.com/site/about/guidelines.xhtml

SR has three phases: (1) *introduction*, (2) *developing* and (3) *maintaining* (outlined in Figure 3). Practitioners will use autonomy-supportive behavioural techniques, including action planning, goal setting and self-monitoring[28, 30]. This will include frequent measurement of body metrics (blood pressure, heart-rate, BMI) to enable goal-setting and bio-feedback to encourage adherence. The exercise component will be delivered in local community-based exercise facilities, with the last phase offered virtually if preferred. The dietary component will either be face-to-face or virtually according to individual participant preference.

Supervised exercise session will be split between HIIT, using the Norwegian 4x4 model for CAD patients[22], and resistance training. Participants will be set weekly homework exercise to do by their exercise trainer. This will comprise moderate-intensity aerobic exercise for 45-minutes in *introduction* and *developing* phases, and increase to higher-intensity exercise in the *maintaining* phase. A once weekly resistance exercise session using workouts learnt in the supervised session during *introduction* and *developing* will be added in the *maintaining* phase.

Dietary support will consist of 1:1 sessions with a dietitian who will work with patients using pre-session combined photographic/written diet diaries to identify incremental areas of improvement in a straightforward manner. A SR introductory educational booklet incorporating diet structure will be provided. Recognising that there is no “one-size-fits-all” pattern, the dietitian will work with the patient to identify residual barriers to dietary change and potential solutions and, where indicated, continue to highlight the importance of smoking cessation. Super Rehab manuals will be provided to exercise trainers and dietitians to support the delivery of a consistent programme across centres and practitioners.

For peer review only - http://bmjopen.bmj.com/site/about/guidelines.xhtml

Data analysis plan

Data collection

Qualitative:

SR participants will be contacted by phone for short interim acceptability assessments at the end of "introduction" and "developing" phases. Upon completion of the study, a full interview will capture all ranges of experiences. This will seek feedback on trial participation, including barriers and facilitators to trial procedures, adherence, and explore any unanticipated effects of taking part. Usual Care participants will be invited for a short phone call to discuss their experience of trial participation. Encrypted interviews will be recorded, transcribed verbatim (by an external transcription company) and analysed using both narrative and thematic analysis[56]. Anonymised patients who decline the interview will be asked a very brief set of questions on reasons for refusal.

In-depth interviews will be conducted with all practitioners involved in delivering SR to identify areas that need to be adapted or changed to implement the intervention in a future trial. Specifically, this will include their experience of delivering and supporting SR, the quality of the training resources, and how participation affected their other work and relationships with patients.

Health Economics Assessment:

This study will consider the feasibility of collecting the data needed for a full health economic analysis in a subsequent RCT. The primary perspective will be the NHS and personal social services[57], but data will also be collected on costs incurred by patients. Training-related costs for participating staff and intervention activity data will be recorded by staff delivering SR using a study-specific form. At each assessment, patients will be asked to supply data on resource use via a questionnaire based on the Client Service Receipt Inventory[58]. Resource use data will be costed using national sources (e.g. National Tariffs and Formulary). The EQ-5D-5L data provides the basis for

For peer review only - http://bmjopen.bmj.com/site/about/guidelines.xhtml

calculating quality-adjusted life-years (QALYs) specified by NICE for use in the subsequent cost utility analysis[51, 57]. The ICECAP-A capability measure provides a complementary assessment of quality-of-life that could provide additional insight into the impact of SR on patients' outcomes in subsequent economic evaluation[52].

Data Management

Once collated, all data will be stored and analysed pseudonymously using a unique identifier code. An electronic cross-referencing list will be stored on an NHS password-protected computer database at each study site. Only pseudonymised data using the unique identifier code will be used on datasets shared with external collaborators. All research records will be destroyed after 10 years.

CTCA artificial intelligence imaging techniques used in the study require image transfer off-site for analysis. For FAI analysis, images will be transferred to the Oxford Translational Cardiovascular Research Group core lab at the University of Oxford. We will use the existing pathway for pseudonymised image transfer via the study sponsor's collaboration with the University of Oxford on The Oxford Risk Factors And Non Invasive Imaging Study (ORFAN), which has separately been approved by the South Central - Oxford C - Research Ethics Committee (Reference 15/SC/0545). For participants recruited at North Bristol NHS Trust, all acquired CT imaging will also be pseudononymised and transferred via PACS to the sponsor for analysis, and to Caristo (company providing FAI analysis for non-ORFAN sites). For FFR_{CT} analysis, images will be sent to HeartFlow, who's core lab (California, US) provide this analysis for NHS hospitals in standard clinical care. Existing secure information sharing protocols for standard clinically indicated scans will be used for image transfer.

For peer review only - http://bmjopen.bmj.com/site/about/guidelines.xhtml

Blinding

The nature of the intervention (SR) prevents blinding of the participants and clinicians to their trial arm. However, statistical, health economic and clinical outcome analyses (including CTCA [FAI, FFR_{CT}, stenosis] and blood results) will be completed blinded to study allocation.

Statistical Analysis

Feasibility measures will be reported with 90% confidence intervals calculated using the Exact Binomial Method. 90% intervals are chosen (rather than 95%) because the upper limits of these measures are not of direct interest for evaluating feasibility.

For the SR group, the proportion of participants adequately completing the intervention and the average number of supervised exercise and nutritional sessions completed will be reported (by intervention phase). The number and proportion of virtual sessions opted for will also be reported. A quantitative analysis of exercise intensity and nutritional change achieved with SR will be undertaken.

The distributional properties of the continuous variables will be examined by plots. Participant characteristics and outcomes will be summarised using descriptive measures: mean (standard deviation) or median (interquartile range) for symmetric or skewed continuous variables, respectively; number (percent) for categorical variables; and mean (standard deviation) absolute and percent change for longitudinal data will be reported separately by treatment allocation. Linear regression analysis of the preliminary data for CAD regression will support power calculations for subsequent RCT. Summary statistics, including missing data, for baseline and follow-up cost data will be reported.

For peer review only - http://bmjopen.bmj.com/site/about/guidelines.xhtml

Success criteria

Feasibility will be assessed against the following success criteria:

1. Recruitment rate: ≥40% of invited eligible participants consent to participate;

2. Retention: ≥70% of participants complete the study;

3. Intervention adherence: ≥70% of SR participants complete ≥75% of sessions;

4. Intervention acceptability: ≥70% of participants consider SR acceptable;

5. Streamlined criteria correctly captures >90% of patients with MetS. If this is <90%, we will examine whether any other combination of measures would be more predictive;

6. Quantitative data collection methods are feasible and acceptable to participants and no substantive issues identified within patient-reported/qualitative data that contraindicate progression to RCT;

7. Successful testing of the proposed cost-effectiveness framework and data collection facilities;

8. Preliminary data for CAD regression to guide power calculations for subsequent RCT.

Criteria 1-5 will be based on the lower 90% confidence limit (CL). For example, success criteria 1 will be met if the lower limit of the 90% exact binomial confidence interval for the recruitment rate is ≥40%. Progression to a full trial will be considered if these criteria are met. If any of the progression targets are not met but fall between 60% and 100% of the target (e.g., having a lower CL for recruitment rate between 24% and 40%) then reasons for this would be considered by the trial management and advisory groups, which may result in changes being made to the trial design for the full trial. If any of the criteria fell below 60% of the target (for example a lower CL for recruitment rate of less than 24%), then progression would not occur without significant and evidence-based changes to design.

For peer review only - http://bmjopen.bmj.com/site/about/guidelines.xhtml

Trial management

Study Governance

The trial management group (TMG) will be led by the co-chief investigators and include the full research team. The TMG will be responsible for overseeing the day-to-day running and management of the trial.

One independent trial advisory group (TAG) will fulfil the roles traditionally undertaken by the trial steering committee and data monitoring committee. It will comprise of an independent cardiologist, health economist, cardiac rehabilitation nurse and a patient representative. TAG will be consulted for advice (e.g., regarding decisions for specific study procedures and processes, including adverse events), and will meet at the beginning, mid-point and end of the study, helping prepare for a subsequent multi-centre RCT.

Adverse events (AEs) and serious AEs (SAEs) will be recorded throughout the study, up to and including final follow-up. Review of AEs will take place during TMG meetings, discussed with the TAG and reported to the sponsor and research ethics committee in line with their guidelines.

Patient and public involvement

The trial will incorporate a Patient Advisory Group (PAG), who aided in development of and adaptations to SR in advance of study initiation. They will hold meetings with the research team during the study to advise on study processes, and will support activities to encourage participant engagement and connectedness to the study. PAG will contribute to study dissemination events, including the lay summary of study findings, and contribute to the development of the grant application bid for the definitive trial.

For peer review only - http://bmjopen.bmj.com/site/about/guidelines.xhtml

Ethics and dissemination:

Ethics approval was granted by South West – Frenchay Research Ethics Committee on 18th January 2022 (reference: 21/SW/0153) and participants will provide written informed consent prior to any study-related activities.

Study findings will be discussed with participants and other relevant stakeholders (commissioners, charities, clinicians and members of the public), including at a planned dissemination event that will be supported by our PAG. Results will be presented at national and international conferences as well as submitted for publication in peer-reviewed journals.

For peer review only

For peer review only - http://bmjopen.bmj.com/site/about/guidelines.xhtml

Acknowledgements:

The authors would like to thank Jane Carter, Sarah Stratton and Jamie Ford, key members of the sponsor's Research & Development department who were instrumental in study set-up; all the personal trainers and dietitians at the participating sites; all members of the cardiac imaging and physiology teams at the participating sites, in particular James Willis and Professor Dan Augustine at the Royal United Hospitals Bath; our lead PPI lead Dr Lisa Austin and Lead Representative Andy Shield, as well as all the members of the PAG and TAG.

Contributors:

JG, AK, DT, JCLR, and FG led on the conception, design and writing of the study. JCLR and DT led the grant application and, as co-chief investigators, take overall responsibility for the trial. JG led on the writing of the study protocol with substantial contributions to the design, writing, critical review of intellectual content and final manuscript approval from DT, JCLR, AK, FG, TS, HM, DM, AD, OP and CA. TS and DT provided statistical expertise in the study design and development stages of the project and the protocol. AK, JG and DT led on Super Rehab conception and design. JG, DT, AK, FG, CA and JCLR made substantial contributions to the trial design and management. FG led on the qualitative study design. HM was responsible for the health economic aspects of the study design and AK, AD, JG and DM were responsible for providing expert clinical support. All authors agree to be accountable for their work.

Funding:

Royal United Hospitals Bath NHS Foundation Trust are the sponsors on behalf of the funder, who is the NIHR as stated below. This paper presents independent research funded by the National Institute for Health Research (NIHR) under its Research for Patient Benefit (RfPB) Programme (Grant

For peer review only - http://bmjopen.bmj.com/site/about/guidelines.xhtml

Reference Number NIHR202811). Further funding was provided by the sponsor's hospital charity, RUHX.

Disclaimer:

The views expressed are those of the author(s) and not necessarily those of the NHS, the NIHR, or the Department of Health and Social Care.

Competing interests:

JCLR reports consulting fees from NHSX, speakers fees from Sanofi, physician services from HeartFlow and is co-founder and share-holder of Heart & Lung Imaging LTD, all outside the submitted work. JG reports speaker fees from Sanofi outside the submitted work. CA is a founder, shareholder, and director of Caristo Diagnostics, a CT image analysis company that provides the perivascular fat attenuation index (FAI) analysis used in the study. FAI is subject to patent US10,695,023B2 and patent applications PCT/GB2017/053262, GB2018/1818049.7, GR20180100490, and GR20180100510, licensed through exclusive license to Caristo Diagnostics.

Patient consent for publication:

Not required.

Ethics approval:

Ethics approval was granted by South West – Frenchay Research Ethics Committee on 18th January 2022 (reference: 21/SW/0153).

For peer review only - http://bmjopen.bmj.com/site/about/guidelines.xhtml

Provenance and peer review:

Not commissioned; peer reviewed for ethical and funding approval prior to submission.

Word Count:

4,103

References:

[1] N. B. Mortensen MB, Dzaye O, Steffensen FH, Bøtker HE, Jensen JM, Rønnow Sand NP, Kragholm KH, Sørensen HT, Leipsic J, Mæng M, Blaha MJ, "Impact of Plaque Burden Versus Stenosis on Ischemic Events in Patients With Coronary Atherosclerosis," *J Am Coll Cardiol*, vol. 76, no. 24, pp. 2803–2813, 2020, doi: 10.1016/j.jacc.2020.10.021.

[2] R. Bhindi, M. Guan, Y. Zhao, K. H. Humphries, and G. B. J. Mancini, "Coronary atheroma regression and adverse cardiac events: A systematic review and meta-regression analysis," *Atherosclerosis*, vol. 284, no. March, pp. 194–201, 2019, doi: 10.1016/j.atherosclerosis.2019.03.005.

[3] M. J. Budoff *et al.*, "Effect of icosapent ethyl on progression of coronary atherosclerosis in patients with elevated triglycerides on statin therapy: final results of the EVAPORATE trial," *Eur Heart J*, pp. 1–8, 2020, doi: 10.1093/eurheartj/ehaa652.

[4] S. J. Hallberg, V. M. Gershuni, and S. J. Athinarayanan, "Reversing type 2 diabetes: A narrative review of the evidence," *Nutrients*, vol. 11, no. 4, pp. 1–16, 2019, doi: 10.3390/nu11040766.

[5] B. Spring *et al.*, "Healthy Lifestyle Change and Subclinical Atherosclerosis in Young Adults," *Circulation*, vol. 130, no. 1, pp. 10–17, 2014, doi: 10.1161/circulationaha.113.005445.

[6] P. K. Jonathan Myers, Paul McAuley, Carl J Lavie, Jean-Pierre Despres, Ross Arena, "Physical activity and cardiorespiratory fitness as major markers of cardiovascular risk: their independent and interwoven importance to health status," *Prog Cardiovasc Dis .*, vol. 57, no. 4, pp. 306–14, 2015.

[7] K. Dale, H, Brassington, L and King, "The impact of healthy lifestyle interventions on mental health and wellbeing: a systematic review," *Mental Health Review Journal*, vol. 19, no. 1, pp. 1–26, 2014.

[8] B. Rauch *et al.*, "The prognostic effect of cardiac rehabilitation in the era of acute revascularisation and statin therapy: A systematic review and meta-analysis of randomized and non-randomized studies - The Cardiac Rehabilitation Outcome Study (CROS)," *Eur J Prev Cardiol*, vol. 23, no. 18, pp. 1914–1939, 2016, doi: 10.1177/2047487316671181.

For peer review only - http://bmjopen.bmj.com/site/about/guidelines.xhtml

[9] L. Anderson *et al.*, “Exercise-Based Cardiac Rehabilitation for Coronary Heart Disease Cochrane Systematic Review and Meta-Analysis,” *J Am Coll Cardiol*, vol. 67, no. 1, pp. 1–12, 2016, doi: 10.1016/j.jacc.2015.10.044.

[10] and M. M. M. Michael G. Flynn, Brian K. McFarlin, “The Anti-Inflammatory Actions of Exercise Training,” *Am J Lifestyle Med*, vol. 1, no. 3, pp. 220–235, 2007.

[11] D. Ornish *et al.*, “Intensive lifestyle changes for reversal of coronary heart disease,” *J Am Med Assoc*, vol. 280, no. 23, pp. 2001–2007, 1998, doi: 10.1001/jama.280.23.2001.

[12] S. Tani *et al.*, “Coronary plaque regression and lifestyle modification in patients treated with pravastatin - Assessment mainly by daily aerobic exercise and an increase in the serum level of high-density lipoprotein cholesterol,” *Circulation Journal*, vol. 74, no. 5, pp. 954–961, 2010, doi: 10.1253/circj.CJ-09-0705.

[13] J. Henzel *et al.*, “High-Risk Coronary Plaque Regression After Intensive Lifestyle Intervention in Nonbstructive Coronary Disease: A Randomized Study,” *JACC Cardiovasc Imaging*, 2021, doi: 10.1016/j.jcmg.2020.10.019.

[14] E. B. Winzer, F. Woitek, and A. Linke, “Physical activity in the prevention and treatment of coronary artery disease,” *J Am Heart Assoc*, vol. 7, no. 4, pp. 1–15, 2018, doi: 10.1161/JAHA.117.007725.

[15] H. S. Lee, M. Chang, M. Piccinelli, and B. K. Koo, “Complete regression of coronary atherosclerosis,” *Eur Heart J*, vol. 41, no. 2, p. 230, 2020, doi: 10.1093/eurheartj/ehz792.

[16] L. Long *et al.*, “Exercise-based cardiac rehabilitation for adults with stable angina,” no. 2, 2018, doi: 10.1002/14651858.CD012786.pub2.

[17] M. C. Fishbein and R. J. Siegel, “How Big Are Coronary Atherosclerotic Plaques That Rupture?,” *Circulation*, vol. 94, no. 10, pp. 2662–2666, Nov. 1996, doi: 10.1161/01.CIR.94.10.2662.

[18] S. SA. Rognmo Ø, Hetland E, Helgerud J, Hoff J, “High intensity aerobic interval exercise is superior to moderate intensity exercise for increasing aerobic capacity in patients with coronary artery disease,” *Eur J Cardiovasc Prev Rehabil*, vol. 11, no. 3, pp. 216–222, 2004.

[19] J. J. Kraal, T. Vromen, R. Spee, H. M. C. Kemps, and N. Peek, “The influence of training characteristics on the effect of exercise training in patients with coronary artery disease : Systematic review and meta-regression analysis,” *Int J Cardiol*, vol. 245, pp. 52–58, 2017, doi: 10.1016/j.ijcard.2017.07.051.

[20] A. E. Tjønna *et al.*, “Aerobic interval training versus continuous moderate exercise as a treatment for the metabolic syndrome: A pilot study,” *Circulation*, vol. 118, no. 4, pp. 346–354, 2008, doi: 10.1161/CIRCULATIONAHA.108.772822.

[21] M. A. Wewege, D. Ahn, J. Yu, K. Liou, and A. Keech, “High-intensity interval training for patients with cardiovascular disease-is it safe? A systematic review,” *J Am Heart Assoc*, vol. 7, no. 21, pp. 1–19, 2018, doi: 10.1161/JAHA.118.009305.

[22] J. L. Taylor *et al.*, “Guidelines for the delivery and monitoring of high intensity interval training in clinical populations,” *Prog Cardiovasc Dis*, vol. 62, no. 2, pp. 140–146, 2019, doi: 10.1016/j.pcad.2019.01.004.

For peer review only

[23] S. Brandhorst and V. D. Longo, "Dietary Restrictions and Nutrition in the Prevention and Treatment of Cardiovascular Disease," *Circ Res*, vol. 124, no. 6, pp. 952–965, 2019, doi: 10.1161/CIRCRESAHA.118.313352.

[24] B. Malinowski *et al.*, "Intermittent fasting in cardiovascular disorders—an overview," *Nutrients*, vol. 11, no. 3, pp. 1–18, 2019, doi: 10.3390/nu11030673.

[25] D. J. Unwin, S. D. Tobin, S. W. Murray, C. Delon, and A. J. Brady, "Substantial and sustained improvements in blood pressure, weight and lipid profiles from a carbohydrate restricted diet: An observational study of insulin resistant patients in primary care," *Int J Environ Res Public Health*, vol. 16, no. 15, 2019, doi: 10.3390/ijerph16152680.

[26] T. Dong, M. Guo, P. Zhang, G. Sun, and B. Chen, "The effects of low-carbohydrate diets on cardiovascular risk factors: A meta-analysis," *PLoS One*, vol. 15, no. 1, pp. 1–16, 2020, doi: 10.1371/journal.pone.0225348.

[27] NACR, "The national audit of cardiac rehabilitation: Annual statistical report," *National Audit of Cardiac Rehabilitation*, 2019, [Online]. Available: https://www.bhf.org.uk/informationsupport/publications/statistics/national-audit-of-cardiac-rehabilitation-quality-and-outcomes-report-2019 [Acessed 20 Dec 2019]

[28] G. B. Samdal, G. E. Eide, T. Barth, G. Williams, and E. Meland, "Effective behaviour change techniques for physical activity and healthy eating in overweight and obese adults; systematic review and meta-regression analyses," *International Journal of Behavioral Nutrition and Physical Activity*, vol. 14, no. 1, pp. 1–14, 2017, doi: 10.1186/s12966-017-0494-y.

[29] & K. W. D. Jerry Suls, Jazmin N. Mogavero, Louise Falzon, Linda S. Pescatello, Emily A. Hennessy, "Health behaviour change in cardiovascular disease prevention and management: meta-review of behaviour change techniques to affect self-regulation," *Health Psychol Rev*, vol. 14, no. 1, pp. 43–65, 2020.

[30] C. J. Greaves *et al.*, "Systematic review of reviews of intervention components associated with increased effectiveness in dietary and physical activity interventions," *BMC Public Health*, vol. 11, no. 1, p. 119, 2011, doi: 10.1186/1471-2458-11-119.

[31] D. Michie, S, Ashford, S, Sniehotta, FF, Dombrowski, SU, Bishop, A, French, "A refined taxonomy of behaviour change techniques to help people change their physical activity and healthy eating behaviours: the CALO-RE taxonomy," *Psychol Health*, vol. 26, no. 11, pp. 1479–1498, 2011.

[32] J. M. Tarkin, M. R. Dweck, and J. H. F. Rudd, "Imaging as a surrogate marker of drug efficacy in cardiovascular disease," *Heart*, vol. 105, no. 7, pp. 567–578, 2019, doi: 10.1136/heartjnl-2017-311213.

[33] K. M. Abdelrahman *et al.*, "Coronary Computed Tomography Angiography From Clinical Uses to Emerging Technologies: JACC State-of-the-Art Review," *J Am Coll Cardiol*, vol. 76, no. 10, pp. 1226–1243, 2020, doi: 10.1016/j.jacc.2020.06.076.

[34] T. J. Stocker *et al.*, "Reduction in radiation exposure in cardiovascular computed tomography imaging: Results from the PROspective multicenter registry on radiaTion dose Estimates of cardiac CT angIOgraphy in daily practice in 2017 (PROTECTION VI)," *Eur Heart J*, vol. 39, no. 41, pp. 3715–3723, 2018, doi: 10.1093/eurheartj/ehy546.

For peer review only - http://bmjopen.bmj.com/site/about/guidelines.xhtml

[35] E. K. Oikonomou *et al.*, "Non-invasive detection of coronary inflammation using computed tomography and prediction of residual cardiovascular risk (the CRISP CT study): a post-hoc analysis of prospective outcome data," *The Lancet*, vol. 392, no. 10151, pp. 929–939, 2018, doi: 10.1016/S0140-6736(18)31114-0.

[36] C. Antoniades, A. S. Antonopoulos, and J. Deanfield, "Imaging residual inflammatory cardiovascular risk," *Eur Heart J*, vol. 41, no. 6, pp. 748–758, 2020, doi: 10.1093/eurheartj/ehz474.

[37] U. Hoffmann *et al.*, "Noninvasive Assessment of Plaque Morphology and Composition in Culprit and Stable Lesions in Acute Coronary Syndrome and Stable Lesions in Stable Angina by Multidetector Computed Tomography," *J Am Coll Cardiol*, vol. 47, no. 8, pp. 1655–1662, 2006, doi: 10.1016/j.jacc.2006.01.041.

[38] J. M. Lee *et al.*, "Identification of High-Risk Plaques Destined to Cause Acute Coronary Syndrome Using Coronary Computed Tomographic Angiography and Computational Fluid Dynamics," *JACC Cardiovasc Imaging*, vol. 12, no. 6, pp. 1032–1043, 2019, doi: 10.1016/j.jcmg.2018.01.023.

[39] A. Chan, J. M. Tetzlaff, and D. G. Altman, "2013 Statement : Defining Standard Protocol Items for Clinical Trials," *Ann Intern Med*, vol. 158, no. 3, pp. 200–207, 2016, doi: 10.7326/0003-4819-158-3-201302050-00583.Requests.

[40] NICE, "Recent-onset chest pain of suspected cardiac origin: assessment and diagnosis," *Clinical guideline [CG95]*, no. November 2016, 2016.

[41] F. Montazerifar, A. Bolouri, M. M. Mozaffar, and M. Karajibani, "The prevalence of metabolic syndrome in coronary artery disease patients," *Cardiol Res*, vol. 7, no. 6, pp. 202–208, 2016, doi: 10.14740/cr507w.

[42] C. Espinola-Klein *et al.*, "Impact of Metabolic Syndrome on Atherosclerotic Burden and Cardiovascular Prognosis," *American Journal of Cardiology*, vol. 99, no. 12, pp. 1623–1628, 2007, doi: 10.1016/j.amjcard.2007.01.049.

[43] H. M. Lakka *et al.*, "The metabolic syndrome and total and cardiovascular disease mortality in middle-aged men," *J Am Med Assoc*, vol. 288, no. 21, pp. 2709–2716, 2002, doi: 10.1001/jama.288.21.2709.

[44] K. G. M. M. Alberti *et al.*, "Harmonizing the metabolic syndrome: A joint interim statement of the international diabetes federation task force on epidemiology and prevention; National heart, lung, and blood institute; American heart association; World heart federation; International ," *Circulation*, vol. 120, no. 16, pp. 1640–1645, 2009, doi: 10.1161/CIRCULATIONAHA.109.192644.

[45] N. Mahalle, M. K. Garg, S. S. Naik, and M. V Kulkarni, "Association of metabolic syndrome with severity of coronary artery disease.," *Indian journal of endocrinology and metabolism*, vol. 18, no. 5. pp. 708–70814, 2014. doi: 10.4103/2230-8210.139238.

[46] E. A. Hulten *et al.*, "Obesity, metabolic syndrome and cardiovascular prognosis: From the Partners coronary computed tomography angiography registry," *Cardiovasc Diabetol*, vol. 16, no. 1, pp. 1–11, 2017, doi: 10.1186/s12933-017-0496-8.

[47] Peter Libby, "Inflammation in atherosclerosis," *Nature*, vol. 420, no. 6917, pp. 868–74, 2002.

For peer review only - http://bmjopen.bmj.com/site/about/guidelines.xhtml

[48] P. Libby, “Mechanisms of Acute Coronary Syndromes and Their Implications for Therapy,” *New England Journal of Medicine*, vol. 368, no. 21, pp. 2004–2013, 2013, doi: 10.1056/nejmra1216063.

[49] D. Silva and A. Pais de Lacerda, “High-sensitivity C-reactive protein as a biomarker of risk in coronary artery disease,” *Revista Portuguesa de Cardiologia (English Edition)*, vol. 31, no. 11, pp. 733–745, 2012, doi: 10.1016/j.repce.2012.09.006.

[50] G. S. Hotamisligil, “Inflammation and metabolic disorders,” *Nature*, vol. 14, no. 444 (7121), pp. 860–7, 2006.

[51] M. H. C. G. A. Lloyd, “Development and preliminary testing of the new five-level version of EQ-5D (EQ-5D-5L),” pp. 1727–1736, 2011, doi: 10.1007/s11136-011-9903-x.

[52] J. Flynn, T, Huynh, E, Peters, T, Al-Janabi, H, Clemens, S, Moody, A, Coast, “Scoring the ICECAP-A capability instrument: estimation of a UK general population tariff,” *Health Econ*, vol. 24, pp. 258–269, 2015.

[53] D. Thompson, D. Markovitch, J. A. Betts, D. Mazzatti, J. Turner, and R. M. Tyrrell, “Time course of changes in inflammatory markers during a 6-mo exercise intervention in sedentary middle-aged men: A randomized-controlled trial,” *J Appl Physiol*, vol. 108, no. 4, pp. 769–779, 2010, doi: 10.1152/japplphysiol.00822.2009.

[54] J. Knuuti *et al.*, “2019 ESC guidelines for the diagnosis and management of chronic coronary syndromes,” *Eur Heart J*, vol. 41, no. 3, pp. 407–477, 2020, doi: 10.1093/eurheartj/ehz425.

[55] NICE, “Surveillance report 2016 – Stable angina: management (2011) NICE guideline CG126,” no. April, 2016.

[56] V. Braun and V. Clarke, “What can ‘thematic analysis’ offer health and wellbeing researchers?,” *Int J Qual Stud Health Well-being*, vol. 9, no. 1, p. 26152, Jan. 2014, doi: 10.3402/qhw.v9.26152.

[57] The National Institute for Health and Care, “Guide to the methods of technology appraisal,” *Nice*, no. April 2013, pp. 1–93, 2018, [Online]. Available: http://doi.org/10.1183/13993003.01815-2018

[58] PSSRU, “Personal Social Services Research Unit Client Service Receipt Inventory (CSRI).” [Online]. Available: https://www.pssru.ac.uk/csri/client-service-receipt-inventory/

For peer review only

Figure legends

Figure 1: Study flow-chart.

Figure 2. Study assessment and intervention timeline. (CTCA = computed tomography coronary angiohgraphy, CPET = cardiopulmonary exercise test, DEXA = Dual-energy X-ray absorptiometry, BP = blood pressure)

For peer review only - http://bmjopen.bmj.com/site/about/guidelines.xhtml

Figure 3. The Super Rehab programme.

For peer review only

For peer review only - http://bmjopen.bmj.com/site/about/guidelines.xhtml

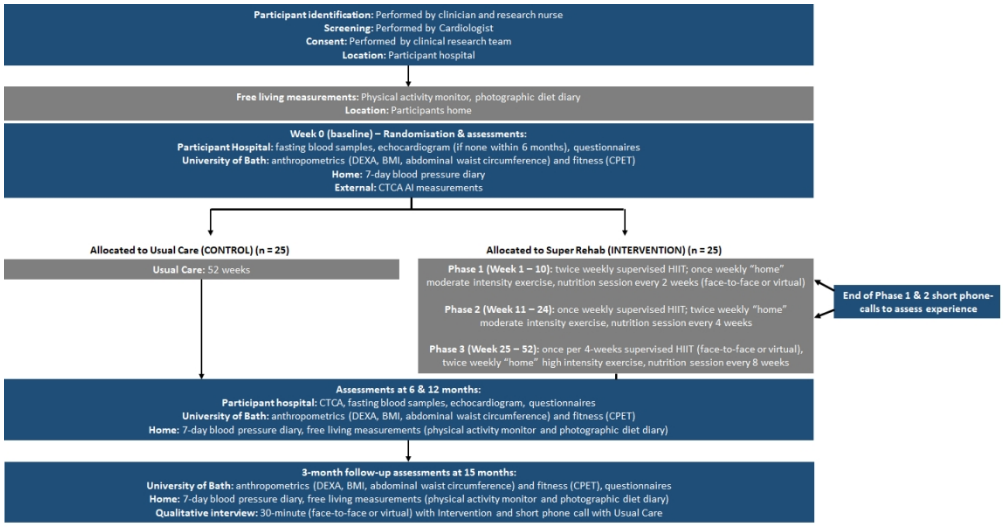

Figure 1: Study flow-chart.

303x159mm (120 x 120 DPI)

For peer review only - http://bmjopen.bmj.com/site/about/guidelines.xhtml

	Usual Care				Suer Rehab + Usual Care						
	Week 0	*Week 26*	*Week 52*	*Week 65*	*Week 0*	*Week 1 - 10*	*Week 11 - 24*	*Week 25 - 52*	*Week 26*	*Week 52*	*Week 65*
**Procedure**											
Screening for eligibility	▪				▪						
Consent	▪				▪						
Baseline data collection	▪				▪						
Randomisation	▪				▪						
Usual Care instituted	▪				▪						
Super Rehab - Introducing phase						▬					
Super Rehab - Developing phase							▬				
Super Rehab - Maintaining phase								▬			
Cardiology clinical review							▪	▪			
CTCA, blood tests, echo	▪	▪	▪		▪				▪	▪	
Questionnaires, CPET, DEXA, anthropometrics, 7-day BP diary	▪	▪	▪	▪	▪				▪	▪	▪
7-day diet diary & physical activity monitor	▪	▪	▪	▪	▪				▪	▪	▪
Interim interview							▪	▪			
End of study interview				▪							▪

Figure 2. Study assessment and intervention timeline. (CTCA = computed tomography coronary angiohgraphy, CPET = cardiopulmonary exercise test, DEXA = Dual-energy X-ray absorptiometry, BP = blood pressure)

273x101mm (120 x 120 DPI)

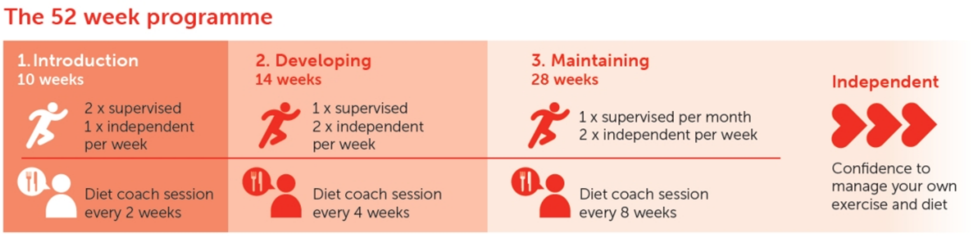

Figure 3. The Super Rehab programme.

115x28mm (300 x 300 DPI)

"Super Rehab": can we achieve coronary artery disease regression? (a feasibility study) – a study protocol

Supplementary material

Super Rehab – Intervention Protocol:

Contents

1. Overview 1
2. Exercise Component - supervised Sessions: 2
3. Exercise component – homework Sessions: 3
4. Dietary component: 3
5. Behavioural Support: 4
6. References: 6

1. Overview

Super Rehab has been designed to enable and support successful behavioural change in patients with cardiovascular disease. The predominant focus is on exercise and diet, and all sessions are delivered 1-to-1. The programme will be explicitly introduced by a Cardiologist, ensuring the programme is presented as a meaningful intervention to improve health outcomes as clinician-led services have been shown to enhance participant engagement[1]. The Cardiologist will introduce the concepts involved in the nutritional advice along with the Super Rehab Booklet, provide advice and oversight to both the dietitian and exercise trainers, and conduct further patient clinical reviews at the end of each intervention phase to review cardiovascular risk factors and medications.

Super Rehab has three phases: (1) *introduction*, (2) *developing* and (3) *maintaining* (see Figure 1). *Introduction* lasts 10-weeks, targeting rapid physiological and behavioural change as a key predictor of long-term success[2]. *Developing* and *maintaining* phases constitute a tapering of direct support to engender longer-term behavioural adherence alongside residual physiological gains. This draws on the principle of enabling participant learning of lifestyle modifications in differentiated blocks[3], and studies demonstrate a 'dose-response' with frequent and sustained interventions achieving more clinically meaningful results[4,5]. Practitioners will use autonomy-supportive behavioural techniques, including action planning, goal setting and self-monitoring[6,7].

The exercise component will be delivered in local community-based exercise facilities, with the last phase offered virtually. The dietary component will either be face-to-face or virtually, depending on individual participant preference.

Phase 1 – Introduction (10 weeks); Exercise: Twice-weekly 1-hour supervised exercise sessions (described below), with once-weekly sessions of prescribed moderate-intensity 'homework'. Weekly body metrics (blood pressure, heart-rate, body mass index [BMI]) will enable goal-setting and biofeedback to encourage adherence. Diet: 30-minute educational sessions every 2-weeks. These will be delivered virtually or built into exercise sessions, minimising patient travel.

Phase 2 – Developing (14 weeks); Exercise: Reduced supervised session frequency to once-weekly, with two sessions of prescribed moderate-intensity homework per week. Body metrics measured

For peer review only - http://bmjopen.bmj.com/site/about/guidelines.xhtml

and fed back 2-weekly. Diet: A 30-minute "touch-base" session once/month, providing ongoing behavioural support of self-monitoring and goals.

Phase 3 - Maintaining (28 Weeks); Exercise: Supervised sessions reduced to one 4-weekly, with twice weekly prescribed sessions of homework (now higher-intensity aerobic exercise) and once weekly resistance exercise session. Body metrics assessed monthly. Participants will be offered the option of having the supervised sessions in this phase delivered virtually or continuing face-to-face. Diet: A 30-minute session 2-monthly, responding to individual barriers and changes in behaviour, where necessary, to support ongoing engagement with nutritional strategy.

Figure 1. The Super Rehab programme.

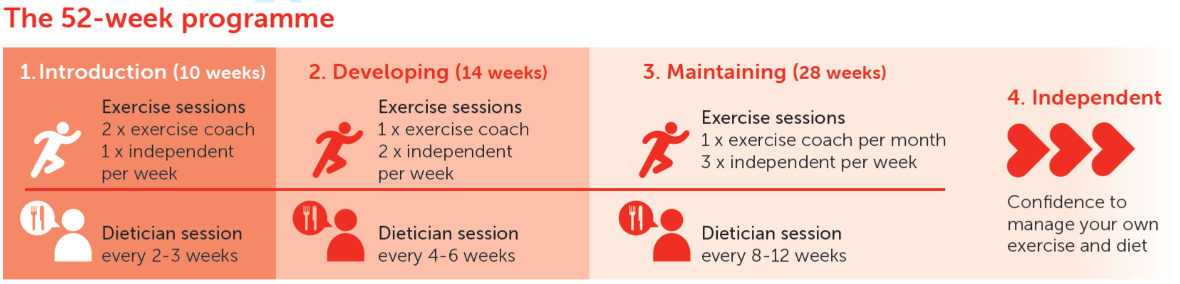

2. Exercise Component - supervised Sessions:

HIIT: Experienced trainers will lead supervised 1:1 exercise sessions incorporating high-intensity interval training (HIIT) based on the Norwegian 4x4 model[8]. This has been repeatedly used in patients with both coronary artery disease (CAD) and metabolic syndrome (MetS), and the evidence-base supports that HIIT is safe in appropriately screened patients[8–10]. At the start of each session, participants will be asked about any change in symptoms or medications and have blood pressure and heart rate measured.

The initial session will consist of a graduated, lead-in of moderate-intensity exercise prior to commencement of HIIT in subsequent sessions. Participants will be taught the Borg Rating of Perceived Exertion[11] (RPE; 6–20) to guide exercise intensity. HIIT will be performed on a static bike (or alternative cardiovascular equipment if unable to cycle) and will comprise a 3-minute warm-up; four 4-minute high-intensity intervals at RPE 15 (hard), finishing at RPE 17-18 (very hard); 3 minutes of active recovery at RPE 11-13 (somewhat hard) between each interval; and conclude with 3–5 minute recovery. Heart rate targets for HIIT (85 – 95% of heart-rate max) will be estimated with a baseline cardiopulmonary exercise test (CPET) to help gauge exercise intensity alongside RPE.

Trainers will help maintain target intensity and monitor participants throughout for concerning symptoms. Heart rate data, final RPE and duration for each high-intensity interval will be recorded for subsequent analysis. The exercise trainer will feedback data (e.g. total virtual distance cycled or power used) to participants as they work through the programme to highlight visible progress being achieved.

The goal as participants progress through the programme will be to gradually increase their overall workload (via watts or speed) on a weekly basis as they become fitter and stronger. To achieve this,

For peer review only - http://bmjopen.bmj.com/site/about/guidelines.xhtml

trainers will gradually increase the bike's resistance or participant's cycle speed, using heart rate response to ensure they remain in their target zone as workload increases.

Resistance Exercises: This has additional benefits, especially for blood pressure[12–14]. Sessions will therefore conclude with twenty minutes of resistance training.

This will be based on a short series of circuit training and will rotate muscle groups between sessions to allow recovery and maximise benefit. During the *introduction* phase trainers will utilise gym resources (free weights and weight machines) to build strength and confidence. In the subsequent developing phase this will switch to resistance against own body weight and bands to ensure patients learn routines that can then be undertaken in their own home in the maintaining phase and beyond.

The specific exercises are not directly prescribed to allow trainers to identify and respond to the individual needs of each participant. They will though be asked to follow this guiding framework and to record the exercises performed:

> *Introducing:* Many patients will be deconditioned, therefore initial sessions will begin with 10 – 15 repetitions at 40 – 50% of the 1 rep-max using free weights or weight machines. When ready (typically after 4 – 5 weeks), progress to 60 – 80% of the individual's 1 repetition maximum.
>
> *Developing & Maintaining:* Move to exercises against gravity / own body weight and use of exercise bands so patients should learn exercises they can start to use on their own. Circuits of 10 – 15 reps at 60 – 80% of the individual's 1 repetition maximum.

3. Exercise component – homework Sessions:

To enhance the weekly workload achieved and support long-term behaviour change, participants will be prescribed 'homework' exercise sessions. This will comprise moderate-intensity aerobic exercise (60 – 75% of heart-rate max) for 45-minutes in induction and consolidation phases (e.g., brisk walking), and increase to higher-intensity exercise (e.g., hill walking or jogging) in the maintenance phase. A once weekly resistance exercise session using workouts learnt in the supervised session during phases 1 and 2 will be added in phase 3. Participants will be provided with written guidance on effort levels, how to monitor their symptoms, and a heart-rate monitor (MyZone) to support self-directed exercise intensity at the appropriate level. These data will be reviewed at supervised exercise sessions to evaluate progress and provide support and kept for subsequent analysis.

4. Dietary component:

Nutrition therapy, as part of a comprehensive lifestyle intervention, improves and can reverse the components of MetS. The dietary intervention component comprises dietitian-led 1:1 consultations using evidence-based dietary advice for CAD-MetS patients[15–17]. This approach has been shown to improve blood pressure, insulin resistance and glycaemic control, lipid profile, systemic vascular inflammation and BMI[15,16,18–23].

For peer review only - http://bmjopen.bmj.com/site/about/guidelines.xhtml

Patients will complete pre-session combined photographic/written diet diaries and will be provided with an educational booklet incorporating the key dietary messages and structure. Recognising that there is no 'one-size-fits-all' pattern, the dietitian will work with participants to identify residual barriers to dietary change and potential solutions. The photographic diet diary will help highlight incremental areas for improvement in a straightforward manner. The dietitian will take a patient-centred approach to setting relevant short-term goals specific to the individual they are working with, which will be reviewed and built upon in each session.

The principles and guiding framework for the dietary advice will be:

- A focus on dietary patterns, including eating at regular mealtimes with portion control rather than "calorie counting", snack reduction and identifying healthy alternatives;
- Reduced refined carbohydrate intake, avoiding starch-based vegetables and refined grains;
- Reducing added sugars, making use of diet diaries to highlight 'hidden sugars' as well encouraging low glycaemic index foods in diabetic participants;
- Sensible drinking choices, recommending water over smoothies, diet drinks and juices, unsweetened tea/coffee and alcohol in moderation;
- Avoidance of ultra-processed foods, such as highly-processed meat and take-away meals;
- Choosing healthy sources of protein, encouraging plant-based foods such as pulses and nuts, regular oily fish, low-fat dairy products, eggs and lean unprocessed cuts of meat (if desired);
- Avoidance of 'low-fat' products, in favour of natural, whole foods with unsaturated and/or mono-saturated fats;
- Educate patients on the importance of swapping saturated fats for mono- and poly-unsaturated fats. Examples include identifying approaches to increasing natural, whole foods containing unsaturated fats, encouraging nuts, seeds, oily fish, avocado and extra-virgin olive oil;
- Increased dietary fibre, focusing on achieving this via diversifying vegetable intake, which encourages satiety, is good for gut and cardio-metabolic health and is good for blood pressure;
- Salt restriction.

Where indicated, dietitians will also continue to highlight the importance of smoking cessation.

5. Behavioural Support:

All practitioners will be required to use the following behavioural support tools in the delivery of Super Rehab.

➢ *Education*: Practitioners will reinforce the positive health outcomes that can be achieved with the suggested exercise and dietary changes the programme aims to deliver (i.e., helping patients to understand what to do, and why they are being asked to do it). This will build on their Super Rehab Booklet. Prescribed 'homework' exercises will be personalised to each individual participant, establishing how best they can incorporate this into their lifestyle.

➢ *Problem-solving:* Using the principles of a motivational interviewing, or person-centred approach, practitioners will support lifestyle changes by helping participants identify their own barriers or logistical issues to achieving their goals (e.g. money, family or work life, intolerances, time), and how they could overcome these. Example questions and techniques will be provided to trainers in their Super Rehab Manual.

For peer review only - http://bmjopen.bmj.com/site/about/guidelines.xhtml

- *Social Support*: Sustaining long term change is more likely when people have support from their own networks. Participants will be asked to identify their key social support (e.g. partner, friend, child), and how they could help as part of problem solving. Dietitians will be encouraged to recommend participants bring their key social support with them to dietary review sessions, and it will also be suggested that participants perform their homework exercise with them. This will help encourage and support participants, increasing the potential to maintain this lifestyle change in the longer term.

- *Goal-setting & Biofeedback:* All participants will also be set a series of longer-term goals for their involvement in the programme, typically:

  a. ≥10% weight loss with a target BMI of 18.5 – 24.9 kg/m^2
  b. A normal abdominal waist circumference (<80cm women / <94cm men)
  c. Improvement in cardiovascular fitness.

However, during the in-programme review sessions practitioners will focus on the shorter-term, achievable, relevant goals related to the actions they can take and focus on between sessions that will help them achieve these longer-term goals. E.g., a specific change in food type in their diet rather than the broader outcome of weight loss. There will be follow-up on whether these goals have been met in subsequent sessions. Super Rehab practitioners will follow the *SMART* goal-setting principles:

Specific – set a clear target/change for them to make (what, when & where)

Measurable – ensure it can be assessed at future visits

Achievable – ensure the goal is something you (and they) believe they can do

Relevant – select goals that ensure a step-wise improvement

Time-bound – set a realistic time-frame for them to achieve the goal(s) by

Changes in body metrics such as weight and abdominal waist circumference will be used within sessions to feedback on performance – both for positive re-enforcement and to supplement goal-setting. The 1:1 nature of the sessions will be used to develop rapport, engender a working relationship and a level of accountability on the participant to deliver the changes asked of them.

For peer review only

For peer review only - http://bmjopen.bmj.com/site/about/guidelines.xhtml

6. References:

1. Chauhan BF, Jeyaraman M, Mann AS, Lys J, Skidmore B, Sibley KM, Abou-Setta A, Zarychanksi R. Behavior change interventions and policies influencing primary healthcare professionals' practice-an overview of reviews. *Implementation Science*. 2017;12(1).

2. Garber AJ, Abrahamson MJ, Barzilay JI, Blonde L, Bloomgarden ZT, Bush MA, Dagogo-Jack S, DeFronzo RA, Einhorn D, Fonseca VA, Garber JR, Garvey WT, Grunberger G, Handelsman Y, Hirsch IB, et al. Consensus statement by the American association of clinical endocrinologists and American college of endocrinology on the comprehensive type 2 diabetes management algorithm - 2018 executive summary. *Endocrine Practice*. 2018;24(1):91–120.

3. Pérez EA, González MP, Martínez-Espinosa RM, Vila MDM, García-Galbis MR. Practical guidance for interventions in adults with metabolic syndrome: Diet and exercise vs. changes in body composition. *International Journal of Environmental Research and Public Health*. 2019;16(18).

4. Singh N, Stewart RAH, Benatar JR. Intensity and duration of lifestyle interventions for long-term weight loss and association with mortality: A meta-analysis of randomised trials. *BMJ Open*. 2019;9(8):1–10.

5. Dutton, GR, Laitner, MH, Perri M. Lifestyle Interventions for Cardiovascular Disease Risk Reduction: A Systematic Review of the Effects of Diet Composition, Food Provision, and Treatment Modality on Weight Loss. *Curr Atheroscler Rep*. 2014;16(10):442.

6. Samdal GB, Eide GE, Barth T, Williams G, Meland E. Effective behaviour change techniques for physical activity and healthy eating in overweight and obese adults; systematic review and meta-regression analyses. *International Journal of Behavioral Nutrition and Physical Activity*. 2017;14(1):1–14.

7. Greaves CJ, Sheppard KE, Abraham C, Hardeman W, Roden M, Evans PH, Schwarz P. Systematic review of reviews of intervention components associated with increased effectiveness in dietary and physical activity interventions. *BMC Public Health*. 2011;11(1):119.

8. Taylor JL, Holland DJ, Spathis JG, Beetham KS, Wisløff U, Keating SE, Coombes JS. Guidelines for the delivery and monitoring of high intensity interval training in clinical populations. *Progress in Cardiovascular Diseases*. 2019;62(2):140–146.

9. Wewege MA, Ahn D, Yu J, Liou K, Keech A. High-intensity interval training for patients with cardiovascular disease-is it safe? A systematic review. *Journal of the American Heart Association*. 2018;7(21):1–19.

10. Swain D. Vigorous intensity exercise is essentially safe for coronary heart disease patients. *Evidence based medicine*. 2013;18(4):159–160.

11. Borg GA. Psychophysical bases of perceived exertion. *Medicine and science in sports and exercise*. 1982;14(5):377–81.

12. Ihalainen JK, Inglis A, Mäkinen T, Newton RU, Kainulainen H, Kyröläinen H, Walker S. Strength training improves metabolic health markers in older individual regardless of training frequency. *Frontiers in Physiology*. 2019;10(FEB):1–12.

13. Braith RW, Stewart KJ. Resistance exercise training: Its role in the prevention of cardiovascular disease. *Circulation*. 2006;113(22):2642–2650.

For peer review only - http://bmjopen.bmj.com/site/about/guidelines.xhtml

14. Schroeder EC, Franke WD, Sharp RL, Lee D chul. Comparative effectiveness of aerobic, resistance, and combined training on cardiovascular disease risk factors: A randomized controlled trial. *PLoS ONE*. 2019;14(1):1–14.

15. Unwin DJ, Tobin SD, Murray SW, Delon C, Brady AJ. Substantial and sustained improvements in blood pressure, weight and lipid profiles from a carbohydrate restricted diet: An observational study of insulin resistant patients in primary care. *International Journal of Environmental Research and Public Health*. 2019;16(15).

16. Hoyas I, Leon-Sanz M. Nutritional Challenges in Metabolic Syndrome. *Journal of Clinical Medicine*. 2019;8(9):1301.

17. Mach F, Baigent C, Catapano AL, Koskinas KC, Casula M, Badimon L, Chapman MJ, De Backer GG, Delgado V, Ference BA, Graham IM, Halliday A, Landmesser U, Mihaylova B, Pedersen TR, et al. 2019 ESC/EAS Guidelines for the management of dyslipidaemias: Lipid modification to reduce cardiovascular risk. *European Heart Journal*. 2020;41(1):111–188.

18. Dong T, Guo M, Zhang P, Sun G, Chen B. The effects of low-carbohydrate diets on cardiovascular risk factors: A meta-analysis. *PLoS ONE*. 2020;15(1):1–16.

19. DiNicolantonio JJ, OKeefe JH. Added sugars drive coronary heart disease via insulin resistance and hyperinsulinaemia: a new paradigm. *Open Heart*. 2017;4(2):e000729.

20. Threapleton DE, Greenwood DC, Evans CEL, Cleghorn CL, Nykjaer C, Woodhead C, Cade JE, Gale CP, Burley VJ. Dietary fibre intake and risk of cardiovascular disease: Systematic review and meta-analysis. *BMJ (Online)*. 2013;347(December):1–12.

21. Forouhi NG, Krauss RM, Taubes G, Willett W. Dietary fat and cardiometabolic health: Evidence, controversies, and consensus for guidance. *BMJ (Online)*. 2018;361(June):1–8.

22. Brandhorst S, Longo VD. Dietary Restrictions and Nutrition in the Prevention and Treatment of Cardiovascular Disease. *Circulation Research*. 2019;124(6):952–965.

23. Korakas E, Dimitriadis G, Raptis A, Lambadiari V. Dietary composition and cardiovascular risk: A mediator or a Bystander? *Nutrients*. 2018;10(12).

For peer review only

Reporting checklist for protocol of a clinical trial.

Based on the SPIRIT guidelines.

		Reporting Item	Page Number
**Administrative information**			
Title	#1	Descriptive title identifying the study design, population, interventions, and, if applicable, trial acronym	1
Trial registration	#2a	Trial identifier and registry name. If not yet registered, name of intended registry	4
Trial registration: data set	#2b	All items from the World Health Organization Trial Registration Data Set	N/a
Protocol version	#3	Date and version identifier	N/a
Funding	#4	Sources and types of financial, material, and other support	27-28
Roles and responsibilities: contributorship	#5a	Names, affiliations, and roles of protocol contributors	1, 27
Roles and responsibilities:	#5b	Name and contact information for the trial sponsor	27

For peer review only

sponsor contact information			
Roles and responsibilities: sponsor and funder	#5c	Role of study sponsor and funders, if any, in study design; collection, management, analysis, and interpretation of data; writing of the report; and the decision to submit the report for publication, including whether they will have ultimate authority over any of these activities	N/a
Roles and responsibilities: committees	#5d	Composition, roles, and responsibilities of the coordinating centre, steering committee, endpoint adjudication committee, data management team, and other individuals or groups overseeing the trial, if applicable (see Item 21a for data monitoring committee)	24-25

Introduction

Background and rationale	#6a	Description of research question and justification for undertaking the trial, including summary of relevant studies (published and unpublished) examining benefits and harms for each intervention	5-7
Background and rationale: choice of comparators	#6b	Explanation for choice of comparators	7
Objectives	#7	Specific objectives or hypotheses	8-9

For peer review only

Trial design	#8	Description of trial design including type of trial (eg, parallel group, crossover, factorial, single group), allocation ratio, and framework (eg, superiority, equivalence, non-inferiority, exploratory)	8
**Methods: Participants, interventions, and outcomes**			
Study setting	#9	Description of study settings (eg, community clinic, academic hospital) and list of countries where data will be collected. Reference to where list of study sites can be obtained	8 & 11
Eligibility criteria	#10	Inclusion and exclusion criteria for participants. If applicable, eligibility criteria for study centres and individuals who will perform the interventions (eg, surgeons, psychotherapists)	11-13
Interventions: description	#11a	Interventions for each group with sufficient detail to allow replication, including how and when they will be administered	18-20
Interventions: modifications	#11b	Criteria for discontinuing or modifying allocated interventions for a given trial participant (eg, drug dose change in response to harms, participant request, or improving / worsening disease)	N/a

For peer review only

Interventions: adherance	#11c	Strategies to improve adherence to intervention protocols, and any procedures for monitoring adherence (eg, drug tablet return; laboratory tests)	N/a
Interventions: concomitant care	#11d	Relevant concomitant care and interventions that are permitted or prohibited during the trial	N/a
Outcomes	#12	Primary, secondary, and other outcomes, including the specific measurement variable (eg, systolic blood pressure), analysis metric (eg, change from baseline, final value, time to event), method of aggregation (eg, median, proportion), and time point for each outcome. Explanation of the clinical relevance of chosen efficacy and harm outcomes is strongly recommended	8-9 & 14-16
Participant timeline	#13	Time schedule of enrolment, interventions (including any run-ins and washouts), assessments, and visits for participants. A schematic diagram is highly recommended (see Figure)	10
Sample size	#14	Estimated number of participants needed to achieve study objectives and how it was determined, including clinical and statistical assumptions supporting any sample size calculations	13-14
Recruitment	#15	Strategies for achieving adequate participant enrolment to reach target sample size	13-14

For peer review only

Methods: Assignment of interventions (for controlled trials)

Allocation: sequence generation	#16a	Method of generating the allocation sequence (eg, computer-generated random numbers), and list of any factors for stratification. To reduce predictability of a random sequence, details of any planned restriction (eg, blocking) should be provided in a separate document that is unavailable to those who enrol participants or assign interventions	14
Allocation concealment mechanism	#16b	Mechanism of implementing the allocation sequence (eg, central telephone; sequentially numbered, opaque, sealed envelopes), describing any steps to conceal the sequence until interventions are assigned	14
Allocation: implementation	#16c	Who will generate the allocation sequence, who will enrol participants, and who will assign participants to interventions	N/a
Blinding (masking)	#17a	Who will be blinded after assignment to interventions (eg, trial participants, care providers, outcome assessors, data analysts), and how	22
Blinding (masking): emergency unblinding	#17b	If blinded, circumstances under which unblinding is permissible, and procedure for revealing a participant's allocated intervention during the trial	22

For peer review only

**Methods: Data collection, management, and analysis**			
Data collection plan	#18a	Plans for assessment and collection of outcome, baseline, and other trial data, including any related processes to promote data quality (eg, duplicate measurements, training of assessors) and a description of study instruments (eg, questionnaires, laboratory tests) along with their reliability and validity, if known. Reference to where data collection forms can be found, if not in the protocol	14-16, 18, & 20-22
Data collection plan: retention	#18b	Plans to promote participant retention and complete follow-up, including list of any outcome data to be collected for participants who discontinue or deviate from intervention protocols	21, 25
Data management	#19	Plans for data entry, coding, security, and storage, including any related processes to promote data quality (eg, double data entry; range checks for data values). Reference to where details of data management procedures can be found, if not in the protocol	21-22
Statistics: outcomes	#20a	Statistical methods for analysing primary and secondary outcomes. Reference to where other details of the	23

For peer review only

		statistical analysis plan can be found, if not in the protocol	
Statistics: additional analyses	#20b	Methods for any additional analyses (eg, subgroup and adjusted analyses)	N/a
Statistics: analysis population and missing data	#20c	Definition of analysis population relating to protocol non-adherence (eg, as randomised analysis), and any statistical methods to handle missing data (eg, multiple imputation)	N/a

Methods: Monitoring

Data monitoring: formal committee	#21a	Composition of data monitoring committee (DMC); summary of its role and reporting structure; statement of whether it is independent from the sponsor and competing interests; and reference to where further details about its charter can be found, if not in the protocol. Alternatively, an explanation of why a DMC is not needed	24-25
Data monitoring: interim analysis	#21b	Description of any interim analyses and stopping guidelines, including who will have access to these interim results and make the final decision to terminate the trial	N/a
Harms	#22	Plans for collecting, assessing, reporting, and managing solicited and spontaneously reported adverse events and other unintended effects of trial interventions or trial conduct	25

For peer review only

Auditing	#23	Frequency and procedures for auditing trial conduct, if any, and whether the process will be independent from investigators and the sponsor	N/a
**Ethics and dissemination**			
Research ethics approval	#24	Plans for seeking research ethics committee / institutional review board (REC / IRB) approval	3 & 25-26
Protocol amendments	#25	Plans for communicating important protocol modifications (eg, changes to eligibility criteria, outcomes, analyses) to relevant parties (eg, investigators, REC / IRBs, trial participants, trial registries, journals, regulators)	13
Consent or assent	#26a	Who will obtain informed consent or assent from potential trial participants or authorised surrogates, and how (see Item 32)	14
Consent or assent: ancillary studies	#26b	Additional consent provisions for collection and use of participant data and biological specimens in ancillary studies, if applicable	N/a
Confidentiality	#27	How personal information about potential and enrolled participants will be collected, shared, and maintained in order to protect confidentiality before, during, and after the trial	21-22

For peer review only

Declaration of interests	#28	Financial and other competing interests for principal investigators for the overall trial and each study site	27
Data access	#29	Statement of who will have access to the final trial dataset, and disclosure of contractual agreements that limit such access for investigators	N/a
Ancillary and post trial care	#30	Provisions, if any, for ancillary and post-trial care, and for compensation to those who suffer harm from trial participation	N/a
Dissemination policy: trial results	#31a	Plans for investigators and sponsor to communicate trial results to participants, healthcare professionals, the public, and other relevant groups (eg, via publication, reporting in results databases, or other data sharing arrangements), including any publication restrictions	25
Dissemination policy: authorship	#31b	Authorship eligibility guidelines and any intended use of professional writers	N/a
Dissemination policy: reproducible research	#31c	Plans, if any, for granting public access to the full protocol, participant-level dataset, and statistical code	N/a

Appendices

Informed consent materials	#32	Model consent form and other related documentation given to participants and authorised surrogates	N/a
Biological specimens	#33	Plans for collection, laboratory evaluation, and storage of biological specimens for genetic or molecular analysis in	N/a

For peer review only

the current trial and for future use in ancillary studies, if applicable

None The SPIRIT Explanation and Elaboration paper is distributed under the terms of the Creative Commons Attribution License CC-BY-NC. This checklist can be completed online using https://www.goodreports.org/, a tool made by the EQUATOR Network in collaboration with Penelope.ai

For peer review only
