# Supplementary material for: ‘Super Rehab’: can we achieve coronary artery disease regression? A feasibility study protocol
Source: BMJ Open. 2023 Dec 12;13(12):e080735. doi: 10.1136/bmjopen-2023-080735 (PMC10729239; doi:10.1136/bmjopen-2023-080735)
Supplement: Reviewer comments [file bmjopen-2023-080735.reviewer_comments.pdf]

PEER REVIEW HISTORY

BMJ Open publishes all reviews undertaken for accepted manuscripts. Reviewers are asked to complete a checklist review form (**http://bmjopen.bmj.com/site/about/resources/checklist.pdf**) and are provided with free text boxes to elaborate on their assessment. These free text comments are reproduced below.

ARTICLE DETAILS

**TITLE (PROVISIONAL)**	"Super Rehab": can we achieve coronary artery disease regression? (a feasibility study) – a study protocol
**AUTHORS**	Graby, John; Khavandi, Ali; Gillison, Fiona; Smith, Theresa; Murphy, David; Peacock, Oliver; McLeod, Hugh; Dastidar, Amardeep; Antoniades, Charalambos; Thompson, Dylan; Rodrigues, Jonathan

This article was not externally reviewed at BMJ Open. Protocols that have been independently assessed prior to submission to BMJ Open are usually fast-tracked to publication on the grounds that further substantial changes will not be possible. This independent assessment will usually be external, independent review for both a substantial grant award from a non-commercial or government funder and ethics approval.
